# Cell cycle oscillations in a polarity network facilitate state switching by morphogenetic cues

**DOI:** 10.1126/sciadv.aec3379

**Published:** 2026-05-13

**Authors:** KangBo Ng, Hadjar Sebaa, Nisha Hirani, Alex Chizh, Zeno Messi, Tom Bland, Kenji Sugioka, Nathan W. Goehring

**Affiliations:** ^1^ The Francis Crick Institute, 1 Midland Road, NW1 1AT London, UK.; ^2^ Institute for the Physics of Living Systems, University College London, Gower St, WC1E 6BT London, UK.; ^3^ Centre for Developmental Neurobiology, King’s College London, London, UK.; ^4^ Life Sciences Institute, The University of British Columbia, 2350 Health Sciences Mall, BC V6T1Z3, Vancouver, Canada.; ^5^ Department of Zoology, The University of British Columbia, 2350 Health Sciences Mall, BC V6T1Z3 Vancouver, Canada.; ^6^ Department of Biochemistry, University of Oxford, South Parks Road, Oxford OX1 3QU, UK.

## Abstract

The establishment of cell form, fate, and function during morphogenesis requires coordination between cell polarity and developmental cues. To achieve this, cells must establish polarity domains that are stable yet sensitive to guiding cues. Here, we show that *Caenorhabditis elegans* germline blastomeres use a time-varying polarization landscape to resolve this trade-off. Specifically, coupling the PAR polarity network to the oscillatory activity of cell cycle kinase CDK-1 ensures that newborn cells operate in a low feedback regime that lowers barriers to state switching, allowing spatial cues to induce and orient PAR protein asymmetries. As CDK-1 activity rises during mitosis, molecular feedback increases, reinforcing cue-induced asymmetries to yield robust and stable patterning of PAR polarity domains. Consistent with this model, we show that low CDK/feedback regimes destabilize PAR domains but are required for de novo polarization and polarity reorientation by cues. We propose that oscillatory networks represent a general mechanism for dynamically optimizing cellular decision-making landscapes, ensuring robust, signal-induced state switching during development.

## Teaser

Regulation of PAR network feedback by CDK-1 dynamically optimizes a sensitivity-stability trade-off during development.

## INTRODUCTION

A key challenge for developmental signaling networks is to balance signal sensitivity with output stability. Robust signal responses are often attributed to feedback circuits that introduce bistable switch-like behavior or irreversibility into signaling networks (*1*, *2*). However, the same feedback that stabilizes outcomes against perturbations or stochastic variation can also reduce sensitivity, hindering the cells’ ability to respond to changes.

One context where this trade-off is particularly evident is during cell polarization. Cell polarity describes the ability of cells to orient in space and typically involves self-organizing molecular networks, which generate asymmetric protein patterns guided by spatiotemporal cues. One such network is the conserved PAR (*par*-titioning defective) network, which underlies a broad range of developmental processes in metazoans (*3*–*5*), including asymmetric cell division, cell migration, and organization of tissue architecture. As the PAR network is continuously redeployed during development, it must remain sensitive to changing signals and cellular contexts so that polarity is properly oriented with respect to neighboring cells or environmental cues. At the same time, once established, polarity must be sufficiently stable to reliably coordinate downstream processes in the face of perturbations. This dual requirement poses a paradox: Cells that are too sensitive to signals will fail to maintain stable directionality, but introducing excessive stability into the network will hinder the ability of cells to respond and reorient with respect to spatial cues. Resolving this paradox is central to understanding how polarity is integrated into developmental programs.

To address this question, we turned to the highly tractable *Caenorhabditis elegans* germline P lineage as a model. Beginning from the zygote (P0), P lineage blastomeres undergo four iterative rounds of PAR-dependent asymmetric divisions, each giving rise to a P blastomere (i.e., P1, P2, P3, and P4) and another somatic cell that forms a major lineage (i.e., AB, EMS, C, and D) (*6*). These asymmetric divisions must be properly oriented by spatial cues so that each cell is correctly positioned within the embryo. This coordination requires that the polarity of P blastomeres adapt to marked shifts in cellular context, discriminate among competing cues, and respond to changing signals to polarize in the correct orientation. For instance, compared to the zygote, both P1 and P2 polarize from differing initial states in response to distinct developmental cues (*6*–*10*). Thus, development requires polarity to be highly sensitive to various inductive signals yet sufficiently stable to robustly inform downstream pathways, such as spindle position and segregation of fate determinants (*6*, *11*).

**Fig. 1.**
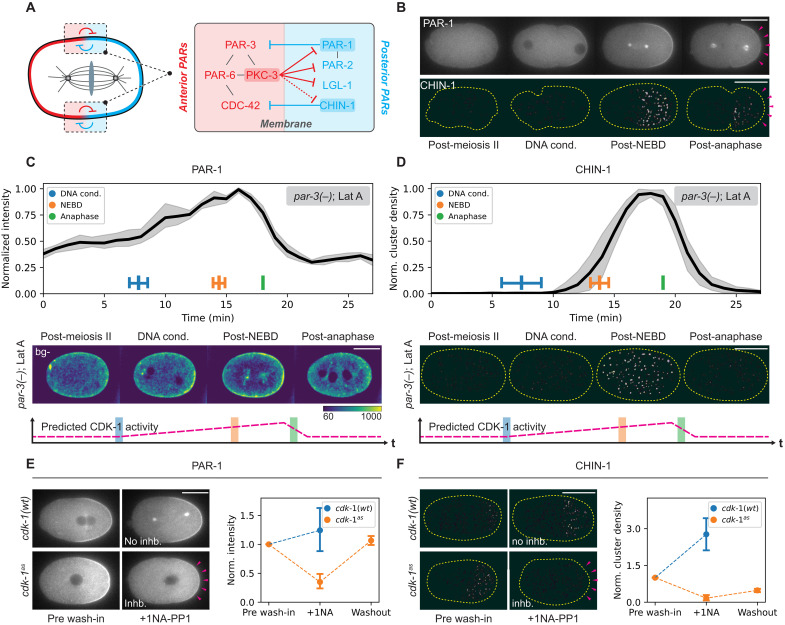
CDK-1 coupled oscillations in PAR behavior. (**A**) Mutually antagonistic feedback mediates segregation of aPARs (red) and pPARs (blue) within membrane-associated domains at opposing poles of the cell. The molecular mechanism linking PKC-3 activity to CHIN-1 antagonism remains unclear (dotted line). (**B**) pPAR effectors PAR-1 and CHIN-1 exhibit oscillations in membrane levels over the cell cycle. Top: Time series of midplane confocal images of embryos expressing PAR-1::GFP (NWG0434, *n* = 4). Bottom: Time series of background-subtracted cortical images of embryos expressing mNG::CHIN-1(NWG0528, *n* = 8). Arrowheads indicate the sharp decrease in membrane levels following anaphase onset. See fig. S1 for quantification. (**C** and **D**) Quantifications and representative background-subtracted images of *par-3(−)*, LatA-treated embryos expressing either PAR-1::GFP (*n* = 5) or mNG::CHIN-1 (*n* = 5). The cell cycle stage was judged by coexpression of H2B::mCherry (not shown). Note that *par-3(−)*, LatA-treated conditions were used to isolate intrinsic pPAR behavior. Bottom: Expected CDK-1 activity from the literature. Times shown are aligned to anaphase onset. (**E**) PAR-1 membrane levels respond to changes in CDK-1 activity. Left: Midsection confocal images of PAR-1::GFP in *cdk-1(wt)* (NWG0042) (*n* = 5) or *cdk-1^as^* embryos (NWG0520) (*n* = 7), acutely treated with 20 μM 1NA-PP1 after pronuclear meeting (PNM). Note the reduction in PAR-1 membrane levels after CDK-1 inhibition (magenta arrowheads). Right: Corresponding quantification showing the reduction in PAR-1 membrane levels and recovery when CDK-1 inhibition is relieved. (**F**) Same as (E) but for cortical images of mNG::CHIN-1 in *cdk-1(wt)* (NWG0451, *n* = 4) or *cdk-1^as^* (NWG0518, *n* = 4) embryos. Quantitations show the mean PAR-1 and CHIN-1 membrane signals across the whole embryo. Mean and 95% confidence interval (bootstrapped) indicated. Scale bars, 20 μm.

Despite blastomere-specific differences, the core principles of polarization are conserved. In each case, polarization involves the cue-induced formation of two opposing, membrane-associated PAR domains, each harboring a distinct subset of PAR proteins termed anterior PARs (aPARs) (*12*–*16*) and posterior PARs (pPARs) (*17*–*20*), respectively (Fig. 1A). Mutual antagonistic feedback subsequently enforces segregation of these two PAR domains: The aPAR effector PKC-3 phosphorylates pPARs to exclude them from aPAR domains (*21*, *22*), whereas the pPAR effectors PAR-1 and CHIN-1 locally suppress aPARs within pPAR domains (*14*, *17*, *22*–*24*). Thus, comparative analysis of P blastomeres provides an ideal framework for dissecting how a single polarity network navigates the dual requirements for sensitivity and stability during development.

Here, we reveal a central role for cell cycle–entrained oscillations in the PAR network in resolving this sensitivity-stability trade-off. Examination of pPAR effectors reveals dynamic oscillation of their membrane localization in phase with cyclin-dependent kinase 1 (CDK-1) activity. The resulting changes in network feedback shift blastomeres from a rheostat-like, low feedback state early in the cell cycle, during which network behavior is highly responsive to instructive cues, to a high feedback, switch-like state that enforces stable patterns as the blastomere enters mitosis. We suggest that the pervasive mitotic oscillations in PAR protein behavior observed across metazoa (*25*, *26*) may reflect an ancestral design principle that allows cells to achieve both the signal sensitivity and output stability required for robust coordination of cell polarity with morphogenesis.

## RESULTS

### CDK-1 drives oscillations in pPAR effector membrane association

The two key pPAR effectors, PAR-1 and CHIN-1, have been reported to exhibit changes in membrane concentration throughout the zygotic cell cycle (*14*, *19*). Quantifications of PAR-1 and CHIN-1 membrane levels with respect to a cell cycle marker [histone 2B (H2B)] revealed tight coupling to mitotic transitions: Membrane concentrations were initially low, first increased following chromosome condensation, peaked around nuclear envelope breakdown (NEBD), and then declined at anaphase onset (Fig. 1B and fig. S1). This pattern of oscillation was repeated in the P1 blastomere, suggesting that P lineage blastomere divisions are characterized by repeated cycles of membrane association and dissociation of pPAR effectors (fig. S1).

Previous works showed that both the aPAR protein PAR-3 (*27*, *28*) and the actomyosin cortex (*22*, *29*) undergo marked changes in behavior during the first cell cycle, both of which could influence cycles of pPAR effector membrane binding. To test this, we repeated our analysis in *par-3(−)* embryos with and without latrunculin A (LatA) (Fig. 1, C and D, and fig. S2). In both cases, although PAR-1 and CHIN-1 membrane localization became uniform due to the loss of aPAR activity in the anterior, the oscillations persisted. Because of the uniform localization and failure to undergo cytokinesis in *par-3(−)* embryos treated with LatA, the cycles of membrane association were particularly clear (Fig. 1, C and D). Last, we used RNA interference (RNAi) to deplete PAR-2, PAR-4, PAR-5, or the centrosomal component SPD-5, all of which have been reported to either stabilize, regulate, or sequester PAR-1 (figs. S2 and S3) (*16*, *24*, *30*–*33*). Again, oscillations persisted following RNAi, suggesting that the coupling of membrane association of pPAR effectors with the cell cycle is independent of other pathways in the PAR network known to affect their behavior.

The pattern of pPAR effector oscillations—low early in the cell cycle, increasing rapidly after mitotic entry and declining at anaphase onset—roughly mirrored the expected activity of the mitotic kinase CDK-1 (*34*–*36*). To explicitly test whether pPAR membrane concentrations were controlled by CDK-1, we generated an analog-sensitive *cdk-1* (*cdk-1^as^*) allele (*37*–*40*). *cdk-1^as^* embryos treated with 1NA-PP1, but not dimethyl sulfoxide (DMSO), showed efficient and reversible cell cycle arrest in prophase. Confirming specific inhibition of CDK-1^AS^ by 1NA-PP1, arrest was not seen when *cdk-1(wt)* lines were treated with 1NA-PP1 (fig. S4A). Acute inhibition of CDK-1^AS^ reduced PAR-1 and CHIN-1 membrane signals, which were partially restored upon drug washout (Fig. 1, E and F, and movies S1 and S2). We further confirmed that these changes in PAR-1 and CHIN-1 membrane levels were not due to potential indirect effects of CDK inhibition on aPAR activity (fig. S4B), as we observed similar results in *par-3(−)* embryos.

Together, these results suggest that pPAR effector oscillations are entrained by CDK-1 activity.

### CDK-1 drives oscillations between low and high feedback states

**Fig. 2.**
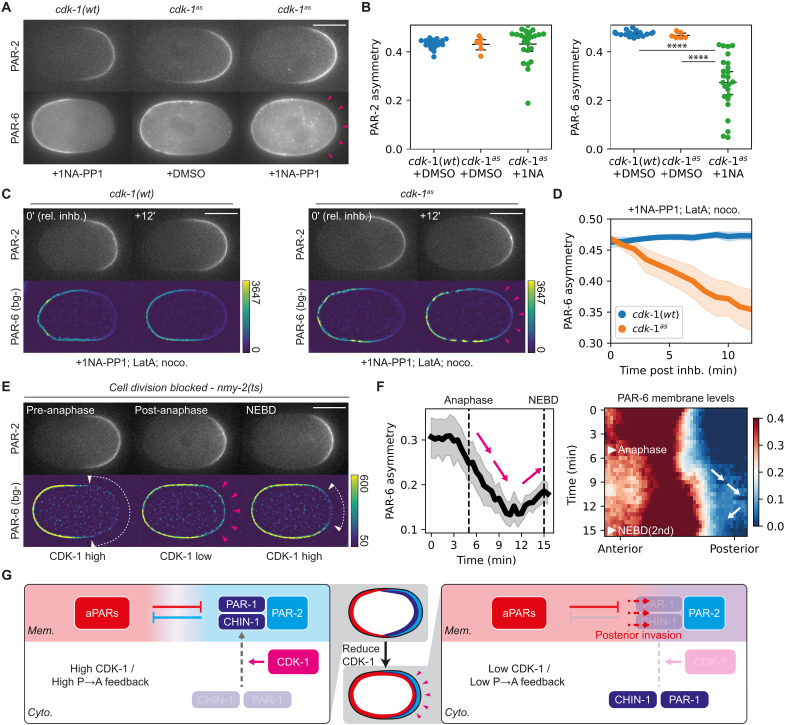
CDK-1 couples PAR feedback strength to the cell cycle. (**A**) CDK-1 inhibition compromises PAR-6 asymmetry. Midsection confocal images of embryos expressing mCherry::PAR-2 and PAR-6::mNG in *cdk-1(wt)* (NWG0268) or *cdk-1^as^* (NWG0443) background, treated with either DMSO or 50 μM 1NA-PP1. Sample sizes: *cdk-1(wt)* + 1NA-PP1 (*n* = 18), *cdk-1^as^* + DMSO (*n* = 8), *cdk-1^as^* + 1NA-PP1 (*n* = 25). Magenta arrowheads highlight PAR-6 in PAR-2 occupied domains. (**B**) Quantification of asymmetry for conditions shown in (A). Each point represents data from a single embryo. (**C**) PAR-6 invasion in CDK-1–inhibited embryos is independent of the cytoskeleton. Midplane confocal images, before and after treatment with 1NA-PP1, nocodazole, and LatA, for embryos expressing mCherry::PAR-2 and PAR-6::mNG in *cdk-1(wt)* (NWG0268, *n* = 6) or *cdk-1^as^* (NWG0443, *n* = 7) backgrounds. Magenta arrowheads highlight PAR-6 invasion into posterior PAR-2 domains. PAR-6 images were background subtracted (bg-) to improve visibility. (**D**) Quantification of PAR-6 asymmetry over time for conditions in (C). (**E**) PAR-6 invasion into PAR-2 domains correlates with the cell cycle stage. Midsection confocal images of an embryo expressing mCherry::PAR-2 and PAR-6::mNG in an *nmy-2(ts)* background (NWG0509) (*n* = 8). Acute temperature upshift was performed after zygote polarization to block cytokinesis. White arrowheads/lines indicate PAR-6 exclusion from PAR-2 domains. Magenta arrowheads highlight PAR-6 in PAR-2 occupied domains. (**F**) Quantification of PAR-6 asymmetry and membrane distribution over time for conditions corresponding to (E). Magenta and white arrows highlight transient loss of PAR-6 asymmetry following anaphase, corresponding to the expected drop in CDK-1 activity. (**G**) Schematic for how CDK-1–dependent membrane binding of PAR-1 and CHIN-1 promotes P→A feedback and aPAR exclusion. Mean and 95% confidence interval (bootstrapped) indicated. Scale bars, 20 μm. Student’s *t* test, unpaired, two-tailed. Scale bars, 20 μm. ****P value < 0.00005.

To assess whether these CDK-1–dependent changes in pPAR effector membrane association correlated with changes in the ability of pPARs to antagonize aPARs (*22*, *24*, *41*–*43*), i.e., pPAR to aPAR (P→A) feedback, we examined the response of the aPAR protein PAR-6 to CDK-1 inhibition. Normally, PAR-6 is excluded by PAR-1 and CHIN-1 from the pPAR domain following polarization (*22*). However, upon inhibition of CDK-1, PAR-6 invaded the posterior domain as marked by PAR-2, leading to overlap of the two proteins at the posterior (Fig. 2, A and B, and fig. S5A). This suggests that CDK-1 inhibition reduces P→A feedback. This phenotype did not appear to stem from off-target effects on the cytoskeleton or regulators downstream of CDK-1 such as AIR-1 and PLK-1, which have also been implicated in PAR polarization (Fig. 2, C and D; fig. S5; and movie S3) (*27*, *44*–*48*).

We next looked at the response of PAR-6 to oscillations in CDK-1 activity through the cell cycle. In wild-type zygotes, we observed posterior spreading of PAR-6 following anaphase onset, when CDK-1 activity and P→A feedback are expected to be low (fig. S6, A and B). However, we are unable to accurately quantify the full extent of the spreading as formation of the cleavage furrow can alter the positioning of PAR proteins (*8*, *49*). To circumvent this, we inhibited cytokinesis via acute inactivation of NMY-2 using a temperature-sensitive *nmy-2(ts)* allele (*50*) or by depleting the formin CYK-1 (*51*, *52*). In both cases, PAR-6 transiently invaded the posterior PAR-2 domain following anaphase onset and was then cleared from the posterior as the embryos reentered mitosis (~NEBD) of the second cell cycle (Fig. 2, E and F, and fig. S6C).

Consistent with this result, we observed cycles of aPAR-pPAR overlap and clearance in later P blastomeres P1 to P3 as previously described (*7*, *8*). Specifically, PAR-6 accumulated steadily throughout the membrane early in the cell cycle, despite these cells being born with uniformly high membrane levels of PAR-2 (fig. S6, D and E). PAR-6 only cleared from PAR-2 domains as the cells entered mitosis, resulting in mutually exclusive polarity domains.

Detecting reduced pPAR to aPAR feedback early in the zygotic (P0) cell cycle is complicated by the fact that pPARs are initially absent from the membrane following meiosis II due to the high initial levels of aPARs at the plasma membrane (*27*). However, by partially depleting PAR-6, we were able to generate zygotes in which pPARs were uniformly enriched on the plasma membrane (fig. S6F). In these zygotes, the residual PAR-6 on the plasma membrane initially overlapped with PAR-2 early in the cell cycle but was rapidly cleared at mitotic entry (NEBD). Together, our data indicate that P→A feedback oscillates in phase with CDK-1 activity, shifting from low feedback early in the cell cycle to high feedback late (Fig. 2G).

### Oscillatory feedback allows robust response to polarizing cues from diverse initial states

We next investigated the potential impact of this oscillation between low and high feedback regimes by turning to a simplified two-component reaction-diffusion model for polarity (see Materials and Methods) (*53*–*56*). As described previously, this system relies on interconversion between membrane-associated and cytoplasmic states, limiting pools, mass conservation, and mutually antagonistic feedback. Previous works have shown that, for high, balanced levels of feedback, the system is multistable, capable of supporting both uniform and polarized states (*53*, *54*).

**Fig. 3.**
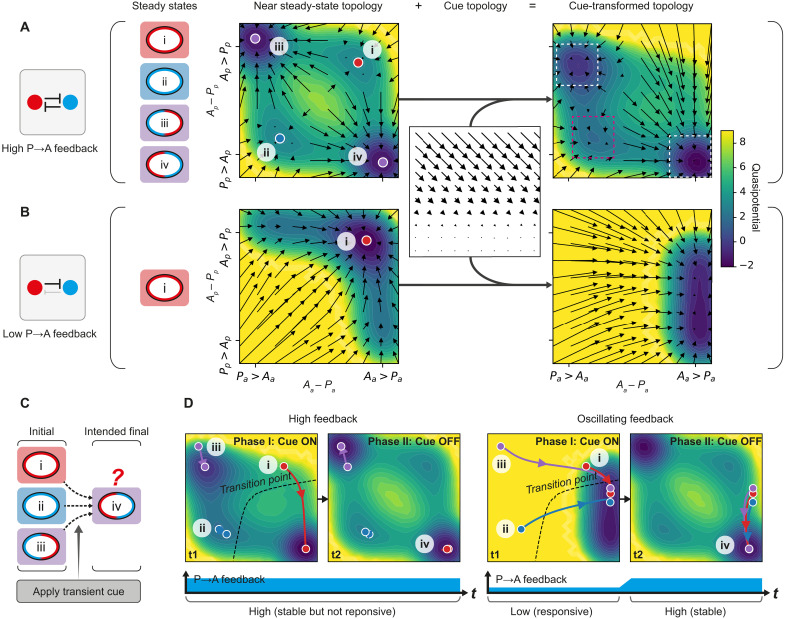
Oscillating feedback facilitates robust and cue-responsive polarization from diverse initial states. For a simplified two-component PAR model, polarity can be mapped to a concentration-difference landscape, defined by the difference between aPARs (*A*) and pPARs (*P*) at the anterior (*A_a_*-*P_a_*) and the posterior (*A_p_*-*P_p_*). System behavior is captured both by how initial states evolve (quivers) or via quasipotential (see Materials and Methods). Note that two-dimensional (2D) projection from four variables results in degeneracy such that system behavior is necessarily an approximation. (**A**) Balanced high feedback (left) yields a single central unstable steady state (not shown) plus four stable steady states (*53*, *54*): uniform *A* high (i; top right), uniform *P* high (ii; bottom left), polarized with *P* high in the anterior (iii; top left), and polarized with *A* high in the anterior (iv; bottom right). Addition of a cue that converts *A_p_* to *A_a_* shifts this landscape and destabilizes the uniform *A* state (i). However, polarized states (white boxes) remain stable and initial uniform P states (magenta box) evolve away from this state at slow rates that are irrelevant on this system’s timescales. (**B**) Low *P→A* feedback supports only a single stable uniform *A* high state (i) and cannot maintain polarity. In the cue-transformed landscape, this stable state shifts to a single polarized state (iv). Thus, the system is cue sensitive but cannot support stable polarity upon cue withdrawal. (**C** and **D**) Whereas a transient cue acting on a high feedback regime can only support transitions from (i) to (iv) (left), an oscillatory regime allows cue-induced transitions from any initial steady state (i to iii). Note that the same behaviors were observed in a mathematically tractable one-component polarity model as well as a full PDE model, confirming that neither coarse graining of space nor the 2D projection of polarity space qualitatively affects our conclusions (figs. S9 and S10).

To comprehensively capture system behavior during polarization, we constructed a landscape of different polarity states, described by the concentration difference between aPARs (*A*) and pPARs (*P*) in the anterior (*A_a_-P_a_*) and in the posterior (*A_p_-P_p_*) (Fig. 3) (*57*). Consistent with the multistable behavior of the system, for any given initial state, the system tends to evolve toward one of four steady states that defines the four quadrants in the landscape: (i) uniform *A* high, (ii) uniform *P* high, (iii) PA polarized (*P* high in anterior, *A* high in posterior), and (iv) AP polarized (*A* high in anterior, *P* high in posterior) (Fig. 3A and fig. S7). Thus, high feedback creates well-defined wells or attractors, with polarization effectively represented by a transition between alternative polarity states. In the case of the zygote, polarization would reflect a transition between states (i) and (iv). Note that, although we do not observe other potential transitions in the zygote [i.e., polarization from a uniform *P* high state (ii to iv) or polarity reversal (iii to iv)], such transitions are observed in later P blastomeres (*7*–*9*) (see below).

In early *C. elegans* embryos, cues are thought to operate via local depletion or inhibition of aPAR activity (*7*, *8*, *21*, *24*, *29*, *53*, *58*, *59*). To represent these cues generically, we implemented a continuous process of fractional redistribution of *A* from posterior to anterior. When transformed with this cue, we found that the uniform *A* high state (i) vanishes and initial states in this quadrant evolve toward the AP polarized state (iv). Thus, consistent with prior analysis, this high feedback regime effectively captures polarization of the zygote. By contrast, initial uniform *P* (ii) and reversed PA polarity states (iii) were more resistant to the cue, with the system remaining trapped near the initial state. Thus, for fixed high feedback and an aPAR-acting cue, proper cue-oriented polarization is only observed for systems beginning in an initially uniform *A* state.

We next considered a low feedback regime in which P→A feedback is reduced (Fig. 3B). For sufficiently low P→A feedback, attractors states reflecting uniform *P* high (ii) and polarized states (iii and iv) vanish, leaving only a single uniform A high attractor (fig. S7). Thus, no stable polarized states are possible. Transforming this low feedback landscape with the cue shifted the single attractor toward the AP polarized quadrant. Thus, in this regime, all initial states converge on a single state exhibiting properly oriented AP polarity (fig. S7). However, when the cue is removed, the attractor reverts to a uniform *A* state and asymmetry is lost. Thus, although a low feedback regime is optimized for ensuring proper cue-induced asymmetry from divergent initial states, the resulting asymmetry is unstable.

Last, we considered an oscillatory feedback regime in which cue induction is accompanied by a transient reduction in P→A feedback, similar to what is observed in embryos, where the low feedback state coincides with the time at which symmetry-breaking cues are active (Fig. 3, C to E, and movies S4 and S5). In this regime, all initial states converge toward a single attractor during the low feedback, cue “on” phase, before evolving toward the AP polarized state (iv) as the system returns to the initial high feedback, cue “off” regime. Thus, by transiently destabilizing the stable attractor states, oscillatory feedback drives convergence of the system from divergent initial states toward a single, stable polarity outcome. For completeness, we examined the behavior of the system when both P→A and A→P feedback were reduced simultaneously (fig. S8). The results were qualitatively similar, confirming the general benefit feedback oscillations have for promoting responsiveness of patterning systems to spatial cues. Specifically, by destabilizing potential attractors that might otherwise trap the system, oscillatory excursions into low feedback regimes provide transient windows of responsiveness to signal-induced changes in state.

Because of the selective nature of the polarity cue in early *C. elegans* embryos, polarization from a uniform *A* state (e.g., the zygote) is not dependent on oscillation in P→A feedback. However, these results predict that oscillations through a transient low feedback state become critical in two situations: (i) when blastomeres must polarize from a uniform *P* state or (ii) when blastomeres must reverse or reorient a preexisting polarized state. As we show below, the P blastomeres P1 and P2, respectively, provide clear examples of these two cases and thus provide an ideal opportunity to test these predictions (*7*–*10*).

### Oscillatory feedback facilitates correct response to polarity cues

**Fig. 4.**
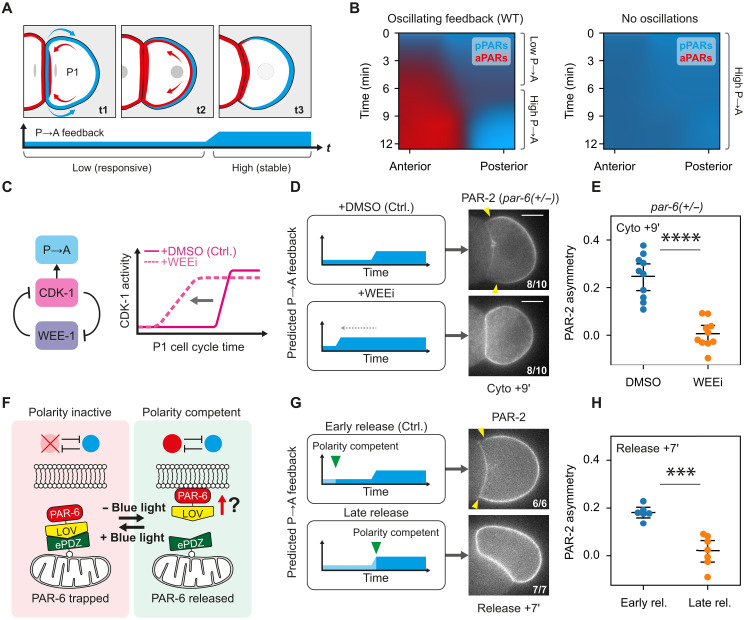
Transient reduction in feedback is required for robust polarization of P1 blastomeres. (**A**) Stages of P1 polarization. t1: PAR-2 (cyan) is enriched throughout the membrane but biased toward the posterior by furrow enrichment of aPARs (red). t2: aPARs load throughout the membrane overlapping with PAR-2. Secondary cues reinforce initial asymmetry. t3: Increasing P→A feedback drives mutual exclusion and emergence of stable, nonoverlapping polarity domains. (**B**) Simulations of a modified PAR model representing P1 polarization suggests a requirement for oscillatory feedback (see figs. S11 and S12). (**C**) Schematic illustrating regulation between WEE-1, CDK-1, and P→A feedback. (**D**) Left: Schematic indicates shortening of high P→A feedback period in WEE-1–inhibited embryos (WEEi; PD0166825). Right: Representative midplane confocal images of GFP::PAR-2 in a *par-6(+/−)* background (NWG0323), acutely treated with either DMSO (*n* = 10) or 20 μM WEEi (PD0166825, *n* = 10). Yellow arrowheads indicate the extent of the polarized PAR-2 domain. (**E**) Corresponding quantification of PAR-2 asymmetry (ASI) 9 min after cell birth. (**F**) Schematic illustrating the optogenetic sequestration of PAR-6. Blue light sequesters PAR-6 at the mitochondria, preventing membrane binding and polarity competence. Without blue light, PAR-6 is free to bind to the membrane, restoring competence for polarization. (**G**) Left: Schematic showing the polarity competence relative to dynamic P→A feedback cycles, comparing early release, where the system becomes polarity competent during the period of low P→A feedback, or late release, where the system becomes polarity competent after P→A feedback is high. Right: Representative midplane confocal images of embryos expressing PAR-6::GFP::LOV, TOMM-20::ePDZ::Halo, and mScarlet-I::PAR-2 (NWG0597), with mitochondria-trapped PAR-6 released early (~3 min) (*n* = 6) or late (~7.5 min) (*n* = 7) after cell birth. Yellow arrowheads indicate the extent of the polarized PAR-2 domain. (**H**) Corresponding quantification of PAR-2 asymmetry (ASI) 7 min after PAR-6 release. ***P < 0.0005 and ****P < 0.00005.

We first focused on the P1 blastomere as a model for polarization from a uniform pPAR initial state (Fig. 4, A and B, and figs. S11 and S12) as theory predicts that a period of low feedback is critical in this context. P1 is born with PAR-2 enriched throughout the membrane and aPARs largely cytoplasmic (*6*). An initial PAR-2 asymmetry is induced by a pool of aPARs that accumulates at the newly formed cell-cell contact site due to advection of aPARs into the cleavage furrow during the previous cytokinesis [Fig. 4A, (t1)] (*8*). Consistent with P→A feedback being low early in the cell cycle, aPARs begin to load throughout the membrane, overlapping with PAR-2. A later wave of actin flows (distinct from the cytokinetic flows) further biases aPARs toward the anterior, whereas the posterior bias of pPARs is enhanced by cooperative membrane binding of PAR-2 [Fig. 4A, (t2), and fig. S12] (*7*, *8*, *24*, *60*, *61*). As P→A feedback increases later in the cell cycle, aPAR and pPAR segregate into two mutually exclusive PAR domains [Fig. 4A, (t3)].

To test our hypothesis, we examined polarization upon perturbation of the initial low feedback period. Although we were unable to completely eliminate the low feedback state early in the P1 cell cycle, we could shorten it through inhibition of the CDK-1 inhibitor WEE-1 (Fig. 4C) (*62*–*65*). Consistent with premature CDK-1 activation, when we inhibited WEE-1, embryos exhibited accelerated loading of PAR-1 and CHIN-1 onto the membrane as well as precocious removal of PAR-6 from PAR-2–enriched membranes (fig. S13). Both effects were blocked by concomitant inhibition of CDK-1, confirming specificity of the effects on WEE-1 inhibition. Note that this premature PAR-6 clearance also occurred when we coadministered LatA with WEE-1 inhibitor, indicating that early clearance was due to increased P→A feedback and not the acceleration or enhancement of late cortical flows. In otherwise wild-type embryos, we found that WEE-1 inhibition did not prevent polarization, suggesting that the initial period of low feedback, albeit shortened, was sufficient in these cases (fig. S13). However, WEE-1 inhibition rendered embryos sensitive to even modest reductions in aPAR levels. Specifically, when we inhibited WEE-1 in heterozygote *par-6(+/−)* embryos, which otherwise polarize normally, polarization was strongly compromised as scored either by qualitative or quantitative measures of PAR-2 asymmetry (Fig. 4, D and E).

As an alternative to truncating the low feedback period, we implemented an optogenetic knock-sideways approach (Fig. 4F, fig. S14, and movie S6) (*66*, *67*). By transiently sequestering PAR-6 at the mitochondrial surface, we could delay when the PAR network becomes competent for polarization and thereby explicitly test whether polarization of P1 must be initiated during the low feedback state. We found that early release during the low feedback period resulted in rapid PAR-6 loading and eventual PAR-2 polarization (Fig. 4, G and H, and fig. S14). By contrast, when we delayed release to a time that corresponded to onset of the high feedback state, PAR-6 was effectively locked out of the membrane and PAR-2 polarization failed.

Together, these results indicate that a period of low P→A feedback is critical to destabilize the initial uniform pPAR high state by facilitating loading of aPARs onto the membrane. In the absence of a low feedback period, the pPAR state becomes locked in, reducing responsiveness to polarity cues.

**Fig. 5.**
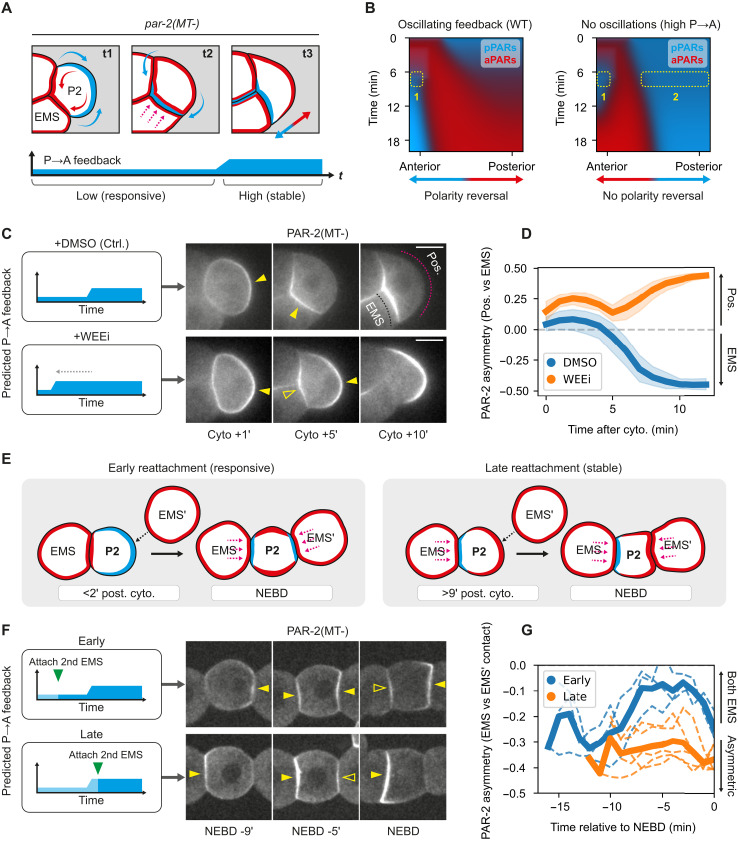
Cue-induced polarity reversal in P2 blastomeres requires a low feedback state. (**A**) Stages of P2 polarization. t1: PAR-2 (cyan) is enriched throughout the membrane but biased toward the posterior by furrow enrichment of aPARs (red). t2: aPARs load throughout the membrane overlapping with PAR-2. Secondary cues reverse the initial asymmetry. t3: Increasing P→A feedback drives mutual exclusion and emergence of stable, nonoverlapping polarity domains. (**B**) Simulations of P2 polarization suggests a requirement for oscillatory feedback for polarity reorientation. Constant high feedback leads to transient appearance of two pPAR domains at both anterior and posterior (dotted rectangles), which is not present in the oscillating regime. (**C**) Left: Predicted feedback dynamics for control/WEE-1 inhibition. Right: Midsection confocal images of GFP::PAR-2(MT-) (NWG0639; PAR-6::mScarlet in fig. S15), treated with DMSO (*n* = 9) or 20 μM WEEi (*n* = 8). Closed arrowheads indicate polarized PAR-2(MT-) domains; open arrowheads indicate the appearance of a second smaller PAR-2(MT-) domain. Magenta and black dotted lines indicate regions used for quantification in (D). (**D**) PAR-2 membrane asymmetry (EMS-contact versus embryo posterior) over time after cytokinesis completion (cell birth) for DMSO/WEEi-treated embryos. Mean and 95% confidence interval (bootstrapped) indicated. (**E**) Schematic of embryo attachment experiments. Dissected EMS-P2 cell pairs were attached with second ectopic EMS, EMS′, either early or late in the cell cycle. (**F**) Left: Predicted feedback levels in P2, when ectopic EMS′ is attached early/late. Right: Midsection confocal images of GFP::PAR-2(MT-) (NWG0192) attached with ectopic EMS′ early (*n* = 3) or late (*n* = 5). Closed arrowheads indicate presence of PAR-2(MT-) domains; open arrowheads indicate second smaller PAR-2(MT-) domains. (**G**) Quantifications for conditions in (F). Mean and individual results shown due to variable cell cycle stages following completion of EMS′ attachment. Scale bars, 20 μm.

Next, we used P2 as a model to investigate the requirement for feedback reduction during cue-induced polarity reversal (Fig. 5, A and B). Similar to P1 blastomeres, PAR-2 is first biased away from the nascent contact due to accumulation of aPARs at the contact by cytokinetic flows [Fig. 5A, (t1)]. However, unlike P1, a MES-1/SRC-1 signaling cue originating from its sister cell EMS then overrides this initial polarity cue, biasing PAR-2 toward the contact site (embryo anterior) instead [Fig. 5A, (t2)]. Last, similar to P1, subsequent increases in feedback later in the cell cycle then promote segregation of aPARs and pPARs into two mutually exclusive PAR domains [Fig. 5A, (t3)].

As above, we first examined the effects of shortening the initial period of low feedback via WEE-1 inhibition. We performed these experiments in embryos harboring a *par-2(R183-5A)* mutation, which affects a putative microtubule and membrane binding region, hereafter *par-2(MT-)* (*24*). Although P2 polarization is normal in these embryos, our previous work showed that the polarity reversal phenotype is more obvious in this genetic background, rendering the system more amenable to analysis (Fig. 5, C and D) (*8*). This is likely due to an increased sensitivity of PAR-2(MT-) to aPAR activity (*24*, *32*), which enhances its response to the initial aPAR asymmetry.

Consistent with prior descriptions, control DMSO-treated *par-2(MT-)* embryos exhibited initial PAR-2 polarization away from the cell contact toward the posterior (Fig. 5, C and D) (*8*, *9*). This posterior enrichment then rapidly dissipated as PAR-2(MT-) was recruited to the EMS:P2 cell contact. Ultimately, PAR-2(MT-) was consolidated to a single anterior domain in the anterior of P2. WEE-1–inhibited embryos also showed initial posterior enrichment; however, in contrast to control embryos, this posterior PAR-2(MT-) domain persisted even as PAR-2 was recruited to the anterior EMS-P2 contact, leading to the transient coexistence of two PAR-2 domains (Fig. 5, C and D, and movie S7). Eventually, the anterior PAR-2(MT-) domain dissipated and was consolidated into the initial posterior domain. In other words, although the effects of the cue are clearly visible in recruiting PAR-2 to the EMS-P2 contact, it cannot outcompete the initial furrow-induced posterior domain, which appears to be locked in by the premature increase in feedback. Thus, polarity reversal fails.

To corroborate the WEE-1 inhibition experiments in P2, we turned to isolated blastomeres, which allowed us to control precisely when P2 received signals from EMS (Fig. 5E). Specifically, isolated EMS-P2 cell pairs were brought into contact with a second, ectopic EMS (EMS′), positioned opposite the native EMS, either early in the P2 cell cycle (0 to 2 min, when feedback is low) or late (9 to 11 min, when feedback is high) (Fig. 5, F and G). In early P2 cells, PAR-2(MT-) exhibited a bias away from the native EMS contact, presumably due to furrow-induced asymmetry of aPARs. Attachment of an ectopic EMS′ to these early P2 cells typically produced PAR-2(MT-) domains of roughly equal size at both EMS contacts (ASI shifts toward 0), consistent with a robust polarization response to both cues (Fig. 5, F and G, and fig. S15). In late P2 cells, PAR-2(MT-) already showed a slight bias toward the native EMS contact. In this case, ectopic EMS′ attachment also generated an additional PAR-2(MT-) domain, consistent with P2 still receiving a signal (fig. S15). However, this secondary domain was unable to compete with the native domain: The domain was markedly weaker than the original and eventually dissipated, with PAR-2(MT-) ultimately consolidating at the native contact by NEBD. These findings are consistent with cue sensitivity being limited to the early phase of the P2 cell cycle, which may explain previous reports that the ability of EMS to reorient pPAR domains was restricted to a narrow time window after birth of P2 (*9*).

These behaviors were well captured by our model when modified to account for the particular features of P1 and P2, suggesting that changes in feedback dynamics alone are sufficient to account for the observed phenotypes (Figs. 4B and 5B and figs. S16 to S18).

Together, our data demonstrate that the cycling of the PAR network into a low feedback regime early in the cell cycle creates a critical cue-sensitive window that allows cells to properly polarize from diverse initial states and reorient polarity in response to directional cues.

## DISCUSSION

**Fig. 6.**
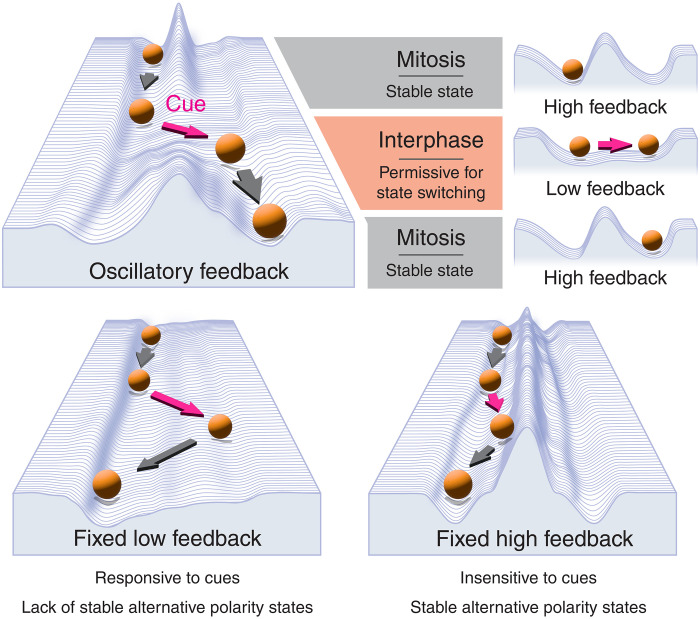
Cell cycle oscillations dynamically tune the polarity landscape to achieve robust state switching. Low and high feedback states optimize for distinct features: Low feedback lowers barriers for polarity state switching but does not stably support distinct states. In this regime, cues (magenta) effectively induce shifts in polarity, but the induced state is not stable and can decay upon cue removal. High feedback not only supports distinct polarity states but also creates steep barriers that hinder state switching. Hence, the systems tend to resist cues and remain in the initial state. Oscillations in feedback strength create transient windows of low feedback in which barriers to polarity state switching are low and cues can induce changes in the polarity. These changes are then effectively locked in as feedback rises, and the barriers to polarity state switching are restored.

Coupling between cell polarity and the cell cycle has been observed in a broad range of biological systems from bacteria to humans (*25*–*27*, *39*, *68*–*76*). However, the role for such coupling is not clear. Mitotic destabilization of polarity has been reported to sensitize proliferative epithelia to oncogenic transformation due the associated disruption of tissue integrity (*76*), raising the question of why transient destabilization is so conserved. Our data indicate that coupling of feedback in the PAR network to CDK-1 activity cycles the system between low and high feedback states, allowing temporal optimization of network activity. The resulting dynamic polarity landscape facilitates cellular responses to guiding cues by transiently reducing barriers to polarity state switching (Fig. 6).

Combining experiments and theory, we show that high antagonistic feedback has opposing effects on cue-driven polarization. On one hand, strong feedback imposes a switch-like behavior in the system, locking in patterns and committing the cell to a specific polarity configuration. However, this comes at the cost of rendering embryos refractory to new cues. Oscillation of PAR activity resolves this conflict by transiently lowering feedback, allowing cells to enter a rheostat-like regime in which the network is highly sensitive to signals and asymmetries largely reflect spatial inputs. These asymmetries can then be reinforced and stabilized as feedback increases again. By temporally ordering periods of network sensitivity and stability, the introduction of cell cycle oscillations in network activity allows cells to sustain robust polarization while providing for transient periods of cue sensitivity that allow tight coordination between polarity and morphogenesis.

Mechanistically, coupling between PAR feedback and CDK-1 appears to occur via gating of the membrane association of pPAR effectors. This is thought to improve P→A feedback by placing the proteins in close proximity to their aPAR substrates and promoting catalytic activation of the effectors themselves (*22*, *24*, *41*–*43*). Both PAR-1– and CHIN-1–related proteins are known to adopt autoinhibited states, arising from allosteric inhibition of their catalytic domains by their own membrane-binding domains (i.e., the kinase domain by KA1 and the GAP domain by C1, respectively) (*41*, *43*). In this model, membrane binding would displace the KA1/C1 domains to relieve autoinhibition. Our data suggest that CDK-1 activity either directly or indirectly mediates this transition into their active, membrane-associated states.

Although we have focused on changes in CDK-1–dependent regulation of pPAR to aPAR feedback, it is important to note that most PAR proteins exhibit some form of cell cycle–dependent behavior (*14*, *22*, *25*, *28*, *39*, *77*–*79*), and we cannot rule out similar effects of feedback oscillations in aPARs. Both PAR-3 and CDC-42 behavior vary in a cell cycle–dependent manner, although direct evidence for changes in aPAR to pPAR (A→P) feedback is currently lacking. Our theoretical analysis indicates that such coincident oscillation of both P→A and A→P feedback has similar effects on balancing stability and sensitivity (fig. S8) and tends to further enhance and generalize the sensitivity of the PAR network to spatial cues, particularly to those that may not act via aPARs as we consider here.

The paradigm of resolving conflict between cue sensitivity and pattern stability via oscillations has roots in theoretical work on reaction-diffusion based models of cell polarity (*80*–*84*). Like the PAR system, these models often rely on stabilizing feedback to support stable polarization in response to weak, transient cues. However, self-stabilizing feedback similarly renders these polarity models resistant to reorientation by secondary cues, posing a problem for systems that must adapt to changing signals (*82*, *83*, *85*). One solution is to incorporate delayed negative feedback into the network, which renders polarity fronts self-limiting. Conceptually similar to what we describe in this study, the resulting periodic destabilization of polarity in these so-called excitable systems provides an opportunity to repolarize in a new direction. In chemotaxing cells, it is thought that such oscillatory behavior balances exploration (sensitivity) and persistence (stability), enabling them to accurately track time-varying signals (*80*, *82*, *83*, *86*). Although these free-running oscillations are ideal for exploratory chemotaxing cells, it is less compatible for systems that need to strictly coordinate polarity behavior with developmental events. In these cases, such as the PAR system, entrainment of oscillations to a developmental controlled clock such as the cell cycle provides an attractive solution as it allows coupling of the sensitivity of the system to guiding cues with developmental events, e.g., cell division.

The widespread prevalence of links between the cell cycle and cell polarity networks suggests that this paradigm of cell cycle–dependent feedback oscillations will be a common solution for balancing the stability of polarized states with their sensitivity to spatial cues, even if the precise timing and mechanisms are not strictly conserved. This includes stem cell systems such as neuroblasts that must coordinate polarity with niche signals (*39*, *72*, *79*, *87*–*89*), dividing epithelial cells, which must ensure proper coordination of both apical basal and planar cell polarity with the surrounding tissue (*70*, *71*, *75*, *76*, *90*–*94*), and budding yeast, which must integrate spatial cues to ensure proper bud site selection (*95*–*97*). In each case, we speculate that low feedback regimes provide critical windows for priming cells to respond and adapt to polarity guiding cues—in some cases effectively resetting polarity—before cells commit to a stable polarized state that is robust to perturbation.

Overall, we suggest that oscillatory signaling dynamics offer systems a way to temporally modulate network dynamics and thus satisfy the often paradoxical requirement for cells to exhibit both sensitivity to signals and stability in outputs. This dynamical systems view of signaling network behavior (*98*–*102*), in this case entrained by the cell cycle, is particularly attractive for understanding how cells optimize their ability to sense, respond, and adapt to the continuously changing demands of developmental programs.

## MATERIALS AND METHODS

### Experimental model and subject details

#### C. elegans: *Strains and culture conditions*

*C. elegans* strains were maintained on OP50 bacterial lawns seeded on nematode growth media (NGM) at 20° or 15°C (for experiments involving temperature-sensitive mutants) under standard laboratory conditions (*103*). Strains harboring optogenetic constructs were grown in a dark box to minimize light exposure. Zygotes were obtained from hermaphrodites unless otherwise noted. Analysis of embryos precludes determination of animal sex.

#### C. elegans: *Transgenic animals*

Point mutations were generated using CRISPR-Cas9, based on the protocol published by Arribere *et al.* (*104*). Briefly, tracrRNA (IDT DNA, 0.5 μl at 100 μM) and crRNA(s) for the target (IDT DNA, 2.7 μl at 100 μM) with duplex buffer (IDT DNA, 2.8 μl) were annealed together (5 min, 95°C) and then stored at room temperature until required. An injection mix containing Cas9 (IDT DNA, 0.5 μl at 10 mg/ml), annealed crRNA, tracrRNA, and the repair template (IDT Ultramer) was incubated at 37°C for 15 min and centrifuged to remove debris (10 min, 13,000 rpm). Young gravid adults were injected along with either a *dpy-10* or *unc-58* co-CRISPR injection marker, and mutants were verified by polymerase chain reaction (PCR) and sequencing (*104*).

Insertion of mNeonGreen was achieved by first generating two PCR products as before (*104*, *105*): one containing the insert DNA sequence and another containing an insert with an additional ~100– to 200–base pair (bp) homology to the insertion site. The products were then column purified (Qiagen, QIAquick PCR purification kit), mixed in equimolar amounts, denatured by heating to 95°C, and annealed through slow cooling to room temperature to generate a pool of products with long single-stranded DNA overhangs that act as the repair template. Similar to the above, an injection mix containing Cas9 (IDT DNA, 0.5 μl at 10 mg/ml), annealed crRNA, tracrRNA, and the repair template was incubated at 37°C for 15 min and centrifuged to remove debris (15 min, 14,100*g*). Young gravid N2 adults were injected along with a *dpy-10* co-CRISPR injection marker, and mutants were identified by PCR and sequence verified. Resulting lines were backcrossed with N2s twice before use.

For designing an analog-sensitive *cdk-1* allele, we first predicted the position of the gatekeeper site of *C. elegans* CDK-1 by aligning an AlphaFold prediction of *C. elegans* CDK-1 structure (AF-P34556-F1-model_v4) with a crystal structure of human CDK-1–cyclin B–Cks2 bound to an adenosine triphosphate (ATP)–competitive inhibitor (5hq0). Combining this with sequence alignment of CDK-1 across various species, we identified the phenylalanine at position 98 (F98) to be the putative gatekeeper site. Mutating CDK-1(F98) to glycine (i.e. Phe^98^→Gly, F98G) as was done in other species led to homozygous embryonic lethality, suggesting that CDK-1 kinase activity was strongly perturbed. It was previously reported that an additional mutation of CDK-1(Met^50^→Val, M50V) partially rescued CDK-1(F98G) kinase activity in mice. However, *cdk-1(M50V, F98G)* was also homozygous lethal. Following advice by J. Janushke (*39*), we introduced a *cdk-1(F98A)* mutation, which yielded homozygous viable worms with embryos that were readily inhibited by the ATP analog 1NA-PP1 as judged by robust cell cycle arrest. This allele was designated *cdk-1^as^.*

#### Bacterial strains

OP50 bacteria and HT115(DE3) were obtained from the Caenorhabditis Genome Center (CGC). Feeding by RNAi used HT115(DE3) bacteria strains carrying the indicated RNAi feeding plasmid.

#### Reagents and resources

Details of reagents and resources in this paper are provided in table S1.

### Method details

#### C. elegans: *RNAi*

RNAi by feeding was performed according to previously described methods (*106*). Briefly, HT115(DE3) bacterial feeding clones were inoculated from LB agar plates to LB liquid cultures and grown overnight at 37°C in the presence of ampicillin (50 μg/ml; until a fairly turbid culture is obtained). To induce high double-stranded RNA (dsRNA) expression, bacterial cultures were then treated with 1 mM isopropyl-β-d-thiogalactopyranoside (IPTG) before spotting 150 μl of cultures onto 60-mm NGM agar plates [supplemented with carbenicillin (10 μg/ml) and 1 mM IPTG] and incubated for 24 hours at 20°C. L3/L4 larvae were then added to RNAi feeding plates and incubated for 16 to 32 hours at either 20° or 25°C. Note that, for characterization of potential roles of the actin cortex (LatA), PAR2, PAR-4, PAR-5, and SPD-5 (RNAi), we used the *par-3(−)* background to ensure that any effects we observed were not due to defects in polarization in these treatments. The uniform distribution of pPARs in *par-3(−)* embryos also made quantification more straightforward.

#### Imaging: Dissection, drug treatment, and mounting for microscopy

Embryos were obtained by dissecting adult worms in 8 to 10 μl of egg buffer [118 mM NaCl, 48 mM KCl, 2 mM CaCl_2_, 2 mM MgCl_2_, and 25 mM Hepes (pH 7.3)] and mounted with 18.8-μm (cortex imaging) or 20-μm (midplane imaging) polystyrene beads (Polysciences Inc.) between a slide and coverslip as in (*107*) and sealed using VALAP (1:1:1, vaseline:lanolin:paraffin wax).

For acute drug treatment experiments, embryos were first permeabilized using either *ptr-2* or *perm-1* fRNAi. For experiments that do not require acute drug addition, embryos were obtained by dissecting adult worms in 8 to 10 μl of Shelton’s Growth Medium (with or without drugs) and mounted with 20.0-μm polystyrene beads between a slide and coverslip and sealed with VALAP as above. For experiments that require acute drug addition, embryos were obtained by dissecting adult worms in 8 to 10 μl of Shelton’s Growth Medium (with or without drugs) and mounted with 18.8-μm polystyrene beads between a large and small coverslip sealed on two parallel edges with VALAP as in Goehring *et al.* (*107*). Buffer exchange was achieved through capillary action by placing a drop of solution at one side of the sample and touching a piece of filter paper at the opposite side. The timings and details of drug treatment experiments are listed in table S2.

#### Imaging: Isolation and reattachment of P2 blastomeres

Ectopic attachment of EMS to P2 blastomeres at different cell cycle stages was achieved by first isolating P1 blastomeres from two-cell-stage embryos as described before (*9*, *108*, *109*). Briefly, adult worms were dissected in an egg salt buffer and the released zygotes were placed into freshly prepared hypochlorite solution [75% Clorox (Clorox) and 2.5 M KOH] for 40 s. Following two washes with Shelton’s growth medium (*110*), embryos were transferred onto the imaging chamber. The eggshell and permeability barrier were removed by repeated mouth pipetting with hand-drawn glass microcapillary tubes (10 μl, Kimble Glass Inc.), yielding two-cell-stage embryos devoid of eggshell and permeability barriers. We then separated P1 from AB by further mouth pipetting and waited for P1 division. Subsequently, an isolated EMS cell (second EMS) was attached to the P2 blastomere at distinct cell cycle stages. The early cell cycle stage of P2 was defined as 0 to 2 min after P1 cytokinesis, whereas late P2 was defined as 9 to 11 min after P1 cytokinesis.

#### Imaging: Acute temperature upshift

Rapid temperature upshift for the *nmy-2(ts)* alleles was achieved by preheating a 100X objective lens to 25.5°C while maintaining the room temperature at 18.5°C (*8*, *50*). Embryos were initially mounted onto an objective lens without a temperature collar at ~18.5°C, and zygotes were tracked and imaged until pronuclear migration (PNM). At this point, the objective lens was swapped with the preheated one, a process that took ~30 to 60 s, and imaging was continued. The disruption of *nmy-2(ts)* activity was confirmed by scoring cytokinesis failure following the temperature upshift.

#### Imaging: Setup for optogenetic knock-sideways experiments

For the optogenetic experiments, worms were only removed from the dark box immediately before experimentation to minimize light exposure. Dissections were performed under minimal light conditions in a dark room with lowlight setting on the dissecting microscope. Optogenetic PAR-6 trapping was induced by exposing embryos to 488-nm blue light in 15-s intervals with 1-s exposure times. The trapping could be reversed by relieving blue light illumination. The release of PAR-6 from mitochondrial-like structures appeared to occur ~2 min after the blue light was turned off.

#### Imaging: Live imaging

Midsection confocal images were captured on a Nikon TiE with a 100x/1.40–numerical aperture (NA) oil objective, further equipped with a custom X-Light V1 spinning disk system (CrestOptics, Rome, Italy) with 50-μm slits, Obis 488/561 fiber-coupled diode lasers (Coherent, Santa Clara, CA), and an Evolve Delta EMCCD camera (Photometrics, Tucson, AZ). Imaging systems were run using Metamorph (Molecular Devices, San Jose, CA) and configured by Cairn Research (Kent, UK). Filter sets were from Chroma (Bellows Falls, VT): ZT488/561rpc, ZET405/488/561/640X, ET535/50m, and ET630/75m. For the optogenetic experiments, an additional blue light filter (Chroma) was added in the differential interference contrast (DIC) light path, on top of the condenser. Imaging of CHIN-1 clusters in acute wash in experiments was achieved as above but by acquiring a stack from the cortex to the midplane of the embryo, enabling us to select the plane best in focus for quantification. This is because WEE-1 inhibition causes cell shape changes to alter significantly throughout the course of imaging, changing the position of the cortical plane.

Cortical imaging of all other experiments were carried out with a 100x/1.40-NA oil objective on a Nikon TiE microscope equipped with an iLas2 TIRF unit (Roper), a custom-made field stop, 488 or 561 fiber-coupled diode lasers (Obis), and an Evolve 512 Delta EMCCD camera (Photometrics), controlled by Metamorph software (Molecular Devices) and configured by Cairn Research. Filter sets were from Chroma: ZT488/561rpc, ZET488/561x, ZET488/561m, ET525/50m, ET630/75m, and ET655LP. Images were captured in bright field, GFP/mNG (ex488/ZET488/561m), and RFP/mKate/mCherry (ex561/ZET488/561m). Simultaneous imaging of CHIN-1 clusters (on the cortex; mNG fluorophore) and histone (deeper into the embryo; mCherry fluorophore) was achieved by using total internal reflection fluorescence (TIRF) illumination on the 488-nm laser, and wide-field illumination on the 561-nm laser, the latter of which is captured as a stack. We also tagged these embryos with NMY-2::mKate, allowing us to find the cortical focal plane of the embryo during interphase, before CHIN-1 clusters emerge as a reference point for the cortical plane.

Blastomere reattachment experiments were imaged using a microscope Olympus IX83 (Olympus), equipped with a spinning disk confocal unit CSU-W1 (Yokogawa), a scientific CMOS camera Prime 95B (Photometrics), a piezoelectric stage NANO-Z (Mad City Labs), a silicon immersion objective UPLSAPO60XS2 (1.3 NA, 60X; Olympus), and a beam splitter Optosplit II (Cairn Research), which is controlled by Cellsense Dimension (Olympus). A silicone immersion oil (Z81114; refractive index: 1.406 at 23°C; Olympus) was used as an immersion medium. Samples were illuminated by a diode-pumped laser with a 488-nm wavelength, and imaging was performed with a 300-ms camera exposure time and 5-s intervals.

All embryos were imaged with a 20°C temperature collar, except for the temperature-sensitive experiments, to which the temperature collar was set at 25.5°C, and for the blastomere dissection experiments, where the temperature is set at 22.5°C.

### Quantification and statistical analysis

#### Image analysis: Segmentation

Different segmentation strategies were used depending on the cell type. P blastomeres were segmented manually using a custom graphical user interface built in Python. In contrast, most zygotes were segmented using a fully automated pipeline based on a convolutional neural network (CNN) built on the U-net architecture. This approach offers advantages over previous methods by achieving high accuracy with DIC images, freeing up a fluorescent channel as no fluorescent markers are required, and enhances segmentation consistency across different lines with varying fluorescent signals. The MobileNetV2 network was chosen as the base model due to its low computational requirements and fast segmentation speed (*111*). Transfer learning was applied, training only the upsampling step of the U-net model with our data. Binary masks that demarcate the embryo perimeter, excluding the eggshell, were provided as the training data. Data augmentation was also used, leading to substantial improvements in validation loss. Notably, the results from automatically segmented images closely match those from manual segmentation when processed with a membrane quantification package developed by T.B., which further refines the regions of interest (ROIs). In addition, the model demonstrated robustness against image artifacts, such as spacer beads and worm debris.

#### Image analysis: Defining anterior and posterior poles in the zygote

The overall geometry of the zygote was first defined by fitting the shape of the ROI to an ellipsoid. The anterior and posterior poles were defined as the ROI coordinates nearest to the tip of each side of the major axis, which can be defined using a custom-built Python graphical user interface.

#### Image analysis: Quantification of membrane profiles

Raw or SAIBR processed images were used for quantification (*112*). To measure cortical concentrations, a 100-pixel-wide (15.5 μm) line following the membrane around the embryo was computationally straightened, and a 20-pixel-wide (3.1 μm) rolling average filter was applied to the straightened image. Intensity profiles perpendicular to the membrane at each position were fit to the sum of a Gaussian component, representing membrane signals, and an error function component, representing cytoplasmic signals, and a constant, representing background signals. Membrane concentrations at each position were calculated as the amplitude of the Gaussian component. This protocol is similar to previously published methods and identical to Ng *et al.* (*8*, *27*, *55*, *113*).

#### Image analysis: Alignment of time series data in P1, P2, and P3

Because PAR domains are more variable in position during polarization in P blastomeres, membrane profiles were aligned throughout the cell cycle for each embryo, followed by alignment between embryos to ensure accurate representation of PAR polarization dynamics. To align membrane concentration profiles throughout the cell cycle of an embryo, membrane profiles that were adjacent in time were averaged; individual profiles within that time span were aligned to the mean, and this process was iterated until a lowest mean squared error was obtained. To align membrane profiles between embryos, an averaged membrane profile for time points around NEBD was used as a reference for each embryo and aligned in a manner identical to above, i.e., the membrane profiles of individual embryos were aligned to the mean of all embryos, and the process was iterated until the lowest mean squared error was achieved. Membrane profiles were also geometrically corrected by automated tracing of the ROIs in both clockwise and anticlockwise directions, and individual embryo profiles were inverted where necessary to minimise the mean squared error of the aligned average profile across embryos.

#### Image analysis: Segmentation and quantification of cortical clusters

To segment CHIN-1 cortical clusters, we first subtracted background from the image using a difference of Gaussians approach and then detected the position and size of each cluster using the Laplacian of Gaussian method. Cluster intensity can then be inferred from the background-subtracted image. Cluster density is defined as the total cluster intensity divided by the area of the cell visible.

#### Image analysis: Background subtraction of PAR-6

Because of the low signal-to-noise ratio of PAR-6, we performed background subtraction on some of the images to enhance visibility on the membrane using a difference of Gaussians approach as before. This was done only for visualization, and none of the quantifications were performed on these background-subtracted images (bg-).

#### Image analysis: ASI

For calculating ASI, the following equation was used: ASI = (*A* − *P*)/[2 ∗ (*A* + *P*)], where *A* and *P* denote the sum of fluorescence signals in the anterior or posterior of the cell, respectively.

#### Image analysis: Quantifying turnover rate of optogenetic experiments

To quantify the rate of PAR-6 release from the mitochondria, we first enhanced the structured PAR-6 signal in the mitochondria by performing a difference of Gaussians background subtraction. This process allowed the pixel intensities of cytoplasmic PAR-6 to approximate a normal distribution centered around zero, whereas the structured PAR-6 trapped in the mitochondria exhibited a tailed distribution toward higher pixel intensities in the histogram. We observed that the tailed distribution of PAR-6 collapsed to approximately a normal distribution around zero 2 min after blue-light release, which suggests that PAR-6 sequestration at the mitochondria can be rapidly reversed.

#### Statistics

All statistical tests were performed in Python and indicated in the figure legends. Data points are shown along with mean values ±95% confidence interval (bootstrapped) unless otherwise noted. Reported *N* are the number of embryos analyzed.

### Model implementation

#### Model: Simplified illustrative PAR system using ODEs

We simplified the PAR system into a set of four ordinary differential equations, describing either aPARs or pPARs at the embryo anterior or posterior. We assumed symmetric reaction rates (table S3) for simplified analysis. The equations were solved using the scipy.odeint function in Python. Briefly, the governing equations are as follows

$$\frac{d\;A_{a}}{dt}=\tilde{D}\left(A_{p}-A_{a}\right)+k_{\text{on}}A_{\text{cyto}}-k_{\text{off}}A_{a}-k_{\mathrm{AP}}P_{a}^{\alpha }A_{a}$$

$$\frac{d\;A_{p}}{dt}=\tilde{D}\left(A_{a}-A_{p}\right)+k_{\text{on}}A_{\text{cyto}}-k_{\text{off}}A_{p}-k_{\mathrm{AP}}P_{p}^{\alpha }A_{p}$$

$$\frac{d\;P_{a}}{dt}=\tilde{D}\left(P_{p}-P_{a}\right)+k_{\text{on}}P_{\text{cyto}}-k_{\text{off}}P_{a}-k_{\mathrm{PA}}A_{a}^{\beta }P_{a}$$

$$\frac{d\;P_{p}}{d\;t}=\tilde{D}\left(P_{a}-P_{p}\right)+k_{\text{on}}P_{\text{cyto}}-k_{\text{off}}P_{p}-k_{\mathrm{PA}}A_{p}^{\beta }P_{p}$$

$$A_{\text{cyto}}=\rho_{A}-\psi \frac{A_{a}+A_{p}}{2}$$

$$P_{\text{cyto}}=\rho_{P}-\psi \frac{P_{a}+P_{p}}{2}$$

where *A_a_*, *A_p_*, *P_a_*, and *P_p_* define aPARs at the anterior, aPARs at the posterior, pPARs at the anterior, and pPARs at the posterior on the membrane, respectively; ρ*_A_* and ρ*_P_* define the total aPAR and pPAR pools; ψ defines the surface area–to–volume ratio; $\tilde{D}$ defines the diffusion-like terms, *k*_on_ and *k*_off_ define the on and off rates; and *k_AP_* and *k_PA_* define *P*→*A* and *A*→*P* feedback, respectively. All parameters for the simplified ODE model are shown in table S3. Simplifying the system allows us to compute the topological landscape more easily, which is achieved by converting the above equation into a stochastic Euler-Maruyama equation (see Supplementary Text). More details on the construction of the phase portrait and introduction of the cue can be found in the Supplementary Text.

#### Model: Mathematically tractable one-species polarity model based on the wave-pinning system

To confirm that oscillatory feedback has the same effects on a mathematically tractable model (i.e., without dimensionality reduction), we constructed a single-species polarity model based on the wave-pinning model (*114*). The governing equations are

d Xad t=D~(Xp−Xa)+konXcyto+koffXa+γXcytoXanKn+Xa n

$$\frac{d\;X_{p}}{d\;t}=\tilde{D}\left(X_{a}-X_{p}\right)+k_{\text{on}}X_{\text{cyto}}+k_{\text{off}}X_{p}+\gamma X_{\text{cyto}}\frac{X_{p}^{n}}{K^{n}+X_{p}^{n}}$$

$$X_{\text{cyto}}=\rho_{X}-\psi \frac{X_{a}+X_{p}}{2}$$

where *X* represents the polarity species. Positive feedback is in the form of a Hill function in which membrane-associated *X* locally recruits *X*_cyto_ additionally. γ defines the feedback strength, and *K* represents the saturation constant. Here, the quasipotential is calculated by solving the Fokker-Planck equation, whereas the quivers were calculated using the instantaneous velocity at each system state. See Supplementary Text for more information. Parameter values can be found in table S4.

#### Model: Representative PAR system using PDEs

A full partial differential equation describing polarization from pPAR dominant (P1-like) and reversed polarized states (P2-like) was simulated using forward Euler’s method with sufficiently small time steps (∂*t* = 0.01) using finite difference discretization, due to dynamic changes in *P*→*A* feedback. The governing equations were written as

$$\partial_{t}A=D_{A}\partial_{x}^{2}A+k_{\text{on},A}A_{\text{cyto}}-k_{\text{off},A}A-k_{\mathrm{AP}}P^{\alpha }A$$

$$\partial_{t}P=D_{P}\partial_{x}^{2}P+k_{\text{on},P}P_{\text{cyto}}-k_{\text{off},P}P-k_{\mathrm{PA}}A^{\beta }P$$

$$A_{\text{cyto}}=\rho_{A}-\psi \bar{A}$$

$$P_{\text{cyto}}=\rho_{P}-\psi \bar{P}$$

where $\bar{A}$ and $\bar{P}$ define membrane averages of aPARs and pPARs. Note that some of the reaction rates here are no longer symmetric.

Here, *k_AP_* and *k_PA_* values were estimated using experimental data through fitting (see table S5). We approximated dynamic changes in *k_AP_* as a function of changes in measured PAR-1 membrane levels during CDK-1 inhibition or throughout the cell cycle. Dynamic effects resulting from the interaction of smooth changes in feedback level with the cue are likely present and will provide additional properties to the network (*115*) but are not further explored as it is outside the scope of this study. Further details on the data fitting and model construction can be found in the Supplementary Text.

## Supplementary Materials

### The PDF file includes:

Supplementary Text Figs. S1 to S18 Tables S1 to S5 Legends for movies S1 to S7 References

## References

[1] H. El-Samad, Biological feedback control—Respect the loops. Cell Syst. 12, 477–487 (2021). 34139160 10.1016/j.cels.2021.05.004

[2] M. Freeman, Feedback control of intercellular signalling in development. Nature 408, 313–319 (2000). 11099031 10.1038/35042500

[3] C. F. Lang, E. Munro, The PAR proteins: From molecular circuits to dynamic self-stabilizing cell polarity. Development 144, 3405–3416 (2017). 28974638 10.1242/dev.139063 PMC5665476

[4] C. E. Buckley, D. St Johnston, Apical–basal polarity and the control of epithelial form and function. Nat. Rev. Mol. Cell Biol. 23, 559–577 (2022). 35440694 10.1038/s41580-022-00465-y

[5] B. Goldstein, I. G. Macara, The PAR proteins: Fundamental players in animal cell polarization. Dev. Cell 13, 609–622 (2007). 17981131 10.1016/j.devcel.2007.10.007 PMC2964935

[6] L. Rose, P. Gönczy, Polarity establishment, asymmetric division and segregation of fate determinants in early *C. elegans* embryos. WormBook 2014, 1–43 (2014). 10.1895/wormbook.1.30.2 25548889

[7] L. A. Koch, L. S. Rose, Multiple pathways for reestablishing PAR polarity in *C. elegans* embryo. Dev. Biol. 500, 40–54 (2023). 37263374 10.1016/j.ydbio.2023.05.005

[8] K. Ng, N. Hirani, T. Bland, J. Borrego-Pinto, S. Wagner, M. Kreysing, N. W. Goehring, Cleavage furrow-directed cortical flows bias PAR polarization pathways to link cell polarity to cell division. Curr. Biol. 33, 4298–4311.e6 (2023). 37729912 10.1016/j.cub.2023.08.076

[9] Y. Arata, J.-Y. Lee, B. Goldstein, H. Sawa, Extracellular control of PAR protein localization during asymmetric cell division in the *C. elegans* embryo. Development 137, 3337–3345 (2010). 20823070 10.1242/dev.054742 PMC2934738

[10] E. Schierenberg, Reversal of cellular polarity and early cell-cell interaction in the embryo of *Caenorhabditis elegans*. Dev. Biol. 122, 452–463 (1987). 3596018 10.1016/0012-1606(87)90309-5

[11] N. T. Rodrigues, T. Bland, K. Ng, N. Hirani, N. W. Goehring, Quantitative perturbation-phenotype maps reveal nonlinear responses underlying robustness of PAR-dependent asymmetric cell division. PLoS Biol. 22, e3002437 (2024). 39652540 10.1371/journal.pbio.3002437 PMC11627365

[12] B. Etemad-Moghadam, S. Guo, K. J. Kemphues, Asymmetrically distributed PAR-3 protein contributes to cell polarity and spindle alignment in early *C. elegans* embryos. Cell 83, 743–752 (1995). 8521491 10.1016/0092-8674(95)90187-6

[13] M. Gotta, M. C. Abraham, J. Ahringer, CDC-42 controls early cell polarity and spindle orientation in *C. elegans*. Curr. Biol. 11, 482–488 (2001). 11412997 10.1016/s0960-9822(01)00142-7

[14] K. T. Kumfer, S. J. Cook, J. M. Squirrell, K. W. Eliceiri, N. Peel, K. F. O’Connell, J. G. White, CGEF-1 and CHIN-1 regulate CDC-42 activity during asymmetric division in the *Caenorhabditis elegans* embryo. Mol. Biol. Cell 21, 266–277 (2010). 19923324 10.1091/mbc.E09-01-0060 PMC2808230

[15] Y. Tabuse, Y. Izumi, F. Piano, K. J. Kemphues, J. Miwa, S. Ohno, Atypical protein kinase C cooperates with PAR-3 to establish embryonic polarity in *Caenorhabditis elegans*. Development 125, 3607–3614 (1998). 9716526 10.1242/dev.125.18.3607

[16] J. L. Watts, D. G. Morton, J. Bestman, K. J. Kemphues, The *C. elegans* par-4 gene encodes a putative serine-threonine kinase required for establishing embryonic asymmetry. Development 127, 1467–1475 (2000). 10704392 10.1242/dev.127.7.1467

[17] A. Beatty, D. Morton, K. Kemphues, The *C. elegans* homolog of *Drosophila* Lethal giant larvae functions redundantly with PAR-2 to maintain polarity in the early embryo. Development 137, 3995–4004 (2010). 21041363 10.1242/dev.056028 PMC2976283

[18] L. Boyd, S. Guo, D. Levitan, D. T. Stinchcomb, K. J. Kemphues, PAR-2 is asymmetrically distributed and promotes association of P granules and PAR-1 with the cortex in *C. elegans* embryos. Development 122, 3075–3084 (1996). 8898221 10.1242/dev.122.10.3075

[19] S. Guo, K. J. Kemphues, par-1, A gene required for establishing polarity in *C. elegans* embryos, encodes a putative Ser/Thr kinase that is asymmetrically distributed. Cell 81, 611–620 (1995). 7758115 10.1016/0092-8674(95)90082-9

[20] C. Hoege, A.-T. Constantinescu, A. Schwager, N. W. Goehring, P. Kumar, A. A. Hyman, LGL can partition the cortex of one-cell *Caenorhabditis elegans* embryos into two domains. Curr. Biol. 20, 1296–1303 (2010). 20579886 10.1016/j.cub.2010.05.061

[21] J. Rodriguez, F. Peglion, J. Martin, L. Hubatsch, J. Reich, N. Hirani, A. G. Gubieda, J. Roffey, A. R. Fernandes, D. S. Johnston, aPKC cycles between functionally distinct PAR protein assemblies to drive cell polarity. Dev. Cell 42, 400–415.e9 (2017). 28781174 10.1016/j.devcel.2017.07.007 PMC5563072

[22] A. Sailer, A. Anneken, Y. Li, S. Lee, E. Munro, Dynamic opposition of clustered proteins stabilizes cortical polarity in the *C. elegans* zygote. Dev. Cell 35, 131–142 (2015). 26460948 10.1016/j.devcel.2015.09.006 PMC5963695

[23] R. Benton, D. St Johnston, Drosophila PAR-1 and 14-3-3 inhibit Bazooka/PAR-3 to establish complementary cortical domains in polarized cells. Cell 115, 691–704 (2003). 14675534 10.1016/s0092-8674(03)00938-3

[24] F. Motegi, S. Zonies, Y. Hao, A. A. Cuenca, E. Griffin, G. Seydoux, Microtubules induce self-organization of polarized PAR domains in *Caenorhabditis elegans* zygotes. Nat. Cell Biol. 13, 1361–1367 (2011). 21983565 10.1038/ncb2354 PMC3208083

[25] A. Noatynska, N. Tavernier, M. Gotta, L. Pintard, Coordinating cell polarity and cell cycle progression: What can we learn from flies and worms? Open Biol. 3, 130083 (2013). 23926048 10.1098/rsob.130083 PMC3758543

[26] S. Doerr, K. Ragkousi, Cell polarity oscillations in mitotic epithelia. Curr. Opin. Genet. Dev. 57, 47–53 (2019). 31465986 10.1016/j.gde.2019.07.007

[27] J. D. Reich, L. Hubatsch, R. Illukkumbura, F. Peglion, T. Bland, N. Hirani, N. W. Goehring, Regulated activation of the PAR polarity network ensures a timely and specific response to spatial cues. Curr. Biol. 29, 1911–1923.e5 (2019). 31155349 10.1016/j.cub.2019.04.058 PMC6584329

[28] D. J. Dickinson, F. Schwager, L. Pintard, M. Gotta, B. Goldstein, A single-cell biochemistry approach reveals PAR complex dynamics during cell polarization. Dev. Cell 42, 416–434.e11 (2017). 28829947 10.1016/j.devcel.2017.07.024 PMC5575849

[29] E. Munro, J. Nance, J. R. Priess, Cortical flows powered by asymmetrical contraction transport PAR proteins to establish and maintain anterior-posterior polarity in the early *C. elegans* embryo. Dev. Cell 7, 413–424 (2004). 15363415 10.1016/j.devcel.2004.08.001

[30] A. W. Folkmann, G. Seydoux, Spatial regulation of the polarity kinase PAR-1 by parallel inhibitory mechanisms. Development 146, dev171116 (2019). 30814118 10.1242/dev.171116 PMC6451319

[31] D. G. Morton, D. C. Shakes, S. Nugent, D. Dichoso, W. Wang, A. Golden, K. J. Kemphues, The *Caenorhabditis elegans* par-5 gene encodes a 14-3-3 protein required for cellular asymmetry in the early embryo. Dev. Biol. 241, 47–58 (2002). 11784094 10.1006/dbio.2001.0489

[32] R. Ramanujam, Z. Han, Z. Zhang, P. Kanchanawong, F. Motegi, Establishment of the PAR-1 cortical gradient by the aPKC-PRBH circuit. Nat. Chem. Biol. 14, 917–927 (2018). 30177850 10.1038/s41589-018-0117-1

[33] Y. Wu, E. E. Griffin, Regulation of cell polarity by PAR-1/MARK kinase. Curr. Top. Dev. Biol. 123, 365–397 (2017). 28236972 10.1016/bs.ctdb.2016.11.001 PMC5943083

[34] W. Bruinsma, M. Aprelia, I. García-Santisteban, J. Kool, Y. J. Xu, R. H. Medema, Inhibition of Polo-like kinase 1 during the DNA damage response is mediated through loss of Aurora A recruitment by Bora. Oncogene 36, 1840–1848 (2017). 27721411 10.1038/onc.2016.347 PMC5378932

[35] O. Gavet, J. Pines, Progressive activation of CyclinB1-Cdk1 coordinates entry to mitosis. Dev. Cell 18, 533–543 (2010). 20412769 10.1016/j.devcel.2010.02.013 PMC3325599

[36] L. Macůrek, A. Lindqvist, D. Lim, M. A. Lampson, R. Klompmaker, R. Freire, C. Clouin, S. S. Taylor, M. B. Yaffe, R. H. Medema, Polo-like kinase-1 is activated by aurora A to promote checkpoint recovery. Nature 455, 119–123 (2008). 18615013 10.1038/nature07185

[37] A. C. Bishop, J. A. Ubersax, D. T. Petsch, D. P. Matheos, N. S. Gray, J. Blethrow, E. Shimizu, J. Z. Tsien, P. G. Schultz, M. D. Rose, A chemical switch for inhibitor-sensitive alleles of any protein kinase. Nature 407, 395–401 (2000). 11014197 10.1038/35030148

[38] M. S. Lopez, J. I. Kliegman, K. M. Shokat, The logic and design of analog-sensitive kinases and their small molecule inhibitors. Methods Enzymol. 548, 189–213 (2014). 25399647 10.1016/B978-0-12-397918-6.00008-2

[39] N. Loyer, E. K. Hogg, H. G. Shaw, A. Pasztor, D. H. Murray, G. M. Findlay, J. Januschke, A CDK1 phosphorylation site on *Drosophila* PAR-3 regulates neuroblast polarisation and sensory organ formation. Elife 13, e97902 (2024). 38869055 10.7554/eLife.97902 PMC11216751

[40] W. Michowski, J. M. Chick, C. Chu, A. Kolodziejczyk, Y. Wang, J. M. Suski, B. Abraham, L. Anders, D. Day, L. M. Dunkl, Cdk1 controls global epigenetic landscape in embryonic stem cells. Mol. Cell 78, 459–476.e13 (2020). 32240602 10.1016/j.molcel.2020.03.010 PMC7214218

[41] R. P. Emptage, M. A. Lemmon, K. M. Ferguson, Molecular determinants of KA1 domain-mediated autoinhibition and phospholipid activation of MARK1 kinase. Biochem. J. 474, 385–398 (2017). 27879374 10.1042/BCJ20160792 PMC5317272

[42] R. P. Emptage, M. A. Lemmon, K. M. Ferguson, R. Marmorstein, Structural basis for MARK1 kinase autoinhibition by its KA1 domain. Structure 26, 1137–1143.e3 (2018). 30099988 10.1016/j.str.2018.05.008 PMC6092042

[43] B. Canagarajah, F. C. Leskow, J. Y. S. Ho, H. Mischak, L. F. Saidi, M. G. Kazanietz, J. H. Hurley, Structural mechanism for lipid activation of the Rac-specific GAP, β2-chimaerin. Cell 119, 407–418 (2004). 15507211 10.1016/j.cell.2004.10.012

[44] K. Klinkert, N. Levernier, P. Gross, C. Gentili, L. von Tobel, M. Pierron, C. Busso, S. Herrman, S. W. Grill, K. Kruse, Aurora A depletion reveals centrosome-independent polarization mechanism in *Caenorhabditis elegans*. Elife 8, e44552 (2019). 30801250 10.7554/eLife.44552 PMC6417861

[45] N. I. Manzi, B. N. de Jesus, Y. Shi, D. J. Dickinson, Temporally distinct roles of Aurora A in polarization of the *C. elegans* zygote. Development 151, dev202479 (2024). 38488018 10.1242/dev.202479 PMC11165718

[46] E. M. Munro, PAR proteins and the cytoskeleton: A marriage of equals. Curr. Opin. Cell Biol. 18, 86–94 (2006). 16364625 10.1016/j.ceb.2005.12.007

[47] J. Nance, J. A. Zallen, Elaborating polarity: PAR proteins and the cytoskeleton. Development 138, 799–809 (2011). 21303844 10.1242/dev.053538 PMC3035085

[48] P. Zhao, X. Teng, S. N. Tantirimudalige, M. Nishikawa, T. Wohland, Y. Toyama, F. Motegi, Aurora-A breaks symmetry in contractile actomyosin networks independently of its role in centrosome maturation. Dev. Cell 48, 631–645.e6 (2019). 30861375 10.1016/j.devcel.2019.02.012

[49] C. Schenk, H. Bringmann, A. A. Hyman, C. R. Cowan, Cortical domain correction repositions the polarity boundary to match the cytokinesis furrow in *C. elegans* embryos. Development 137, 1743–1753 (2010). 20430749 10.1242/dev.040436 PMC3188577

[50] J. Liu, L. L. Maduzia, M. Shirayama, C. C. Mello, NMY-2 maintains cellular asymmetry and cell boundaries, and promotes a SRC-dependent asymmetric cell division. Dev. Biol. 339, 366–373 (2010). 20059995 10.1016/j.ydbio.2009.12.041 PMC2903000

[51] S. R. Naganathan, S. Fürthauer, J. Rodriguez, B. T. Fievet, F. Jülicher, J. Ahringer, C. V. Cannistraci, S. W. Grill, Morphogenetic degeneracies in the actomyosin cortex. Elife 7, e37677 (2018). 30346273 10.7554/eLife.37677 PMC6226289

[52] K. A. Swan, A. F. Severson, J. C. Carter, P. R. Martin, H. Schnabel, R. Schnabel, B. Bowerman, cyk-1: A *C. elegans* FH gene required for a late step in embryonic cytokinesis. J. Cell Sci. 111, 2017–2027 (1998). 9645949 10.1242/jcs.111.14.2017

[53] N. W. Goehring, P. K. Trong, J. S. Bois, D. Chowdhury, E. M. Nicola, A. A. Hyman, S. W. Grill, Polarization of PAR proteins by advective triggering of a pattern-forming system. Science 334, 1137–1141 (2011). 22021673 10.1126/science.1208619

[54] P. K. Trong, E. M. Nicola, N. W. Goehring, K. V. Kumar, S. W. Grill, Parameter-space topology of models for cell polarity. New J. Phys. 16, 065009 (2014).

[55] P. Gross, K. V. Kumar, N. W. Goehring, J. S. Bois, C. Hoege, F. Jülicher, S. W. Grill, Guiding self-organized pattern formation in cell polarity establishment. Nat. Phys. 15, 293–300 (2019). 31327978 10.1038/s41567-018-0358-7 PMC6640039

[56] L. Hubatsch, F. Peglion, J. D. Reich, N. T. Rodrigues, N. Hirani, R. Illukkumbura, N. W. Goehring, A cell-size threshold limits cell polarity and asymmetric division potential. Nat. Phys. 15, 1078–1085 (2019). 10.1038/s41567-019-0601-x PMC6774796 31579399

[57] A. Nandan, A. Koseska, Non-asymptotic transients away from steady states determine cellular responsiveness to dynamic spatial-temporal signals. PLoS Comput. Biol. 19, e1011388 (2023). 37578988 10.1371/journal.pcbi.1011388 PMC10449117

[58] Y. Chang, D. J. Dickinson, A particle size threshold governs diffusion and segregation of PAR-3 during cell polarization. Cell Rep. 39, 110652 (2022). 35417695 10.1016/j.celrep.2022.110652 PMC9093022

[59] R. Illukkumbura, N. Hirani, J. Borrego-Pinto, T. Bland, K. Ng, L. Hubatsch, J. McQuade, R. G. Endres, N. W. Goehring, Design principles for selective polarization of PAR proteins by cortical flows. J. Cell Biol. 222, e202209111 (2023). 37265444 10.1083/jcb.202209111 PMC10238861

[60] Y. Arata, M. Hiroshima, C.-G. Pack, R. Ramanujam, F. Motegi, K. Nakazato, Y. Shindo, P. W. Wiseman, H. Sawa, T. J. Kobayashi, Cortical polarity of the RING protein PAR-2 is maintained by exchange rate kinetics at the cortical-cytoplasmic boundary. Cell Rep. 16, 2156–2168 (2016). 27524610 10.1016/j.celrep.2016.07.047

[61] T. Bland, N. Hirani, D. C. Briggs, R. Rossetto, K. Ng, I. A. Taylor, N. Q. McDonald, D. Zwicker, N. W. Goehring, Optimized PAR-2 RING dimerization mediates cooperative and selective membrane binding for robust cell polarity. EMBO J. 43, 3214–3239 (2024). 38907033 10.1038/s44318-024-00123-3 PMC11294563

[62] C. Featherstone, P. Russell, Fission yeast _p_107^wee1^ mitotic inhibitor is a tyrosine/serine kinase. Nature 349, 808–811 (1991). 1825699 10.1038/349808a0

[63] K. Lundgren, N. Walworth, R. Booher, M. Dembski, M. Kirschner, D. Beach, mik1 and wee1 cooperate in the inhibitory tyrosine phosphorylation of cdc2. Cell 64, 1111–1122 (1991). 1706223 10.1016/0092-8674(91)90266-2

[64] W. M. Michael, Cyclin CYB-3 controls both S-phase and mitosis and is asymmetrically distributed in the early *C. elegans* embryo. Development 143, 3119–3127 (2016). 27578178 10.1242/dev.141226 PMC5047676

[65] P. Russell, P. Nurse, Negative regulation of mitosis by wee1+, a gene encoding a protein kinase homolog. Cell 49, 559–567 (1987). 3032459 10.1016/0092-8674(87)90458-2

[66] A. Milas, M. Jagrić, J. Martinčić, I. M. Tolić, “Optogenetic reversible knocksideways, laser ablation, and photoactivation on the mitotic spindle in human cells” in *Methods in Cell Biology* (Elsevier, 2018), vol. 145, pp. 191–215. 10.1016/bs.mcb.2018.03.024 29957204

[67] M. S. Robinson, D. A. Sahlender, S. D. Foster, Rapid inactivation of proteins by rapamycin-induced rerouting to mitochondria. Dev. Cell 18, 324–331 (2010). 20159602 10.1016/j.devcel.2009.12.015 PMC2845799

[68] A. S. Howell, D. J. Lew, Morphogenesis and the cell cycle. Genetics 190, 51–77 (2012). 22219508 10.1534/genetics.111.128314 PMC3249366

[69] A. Treuner-Lange, L. Søgaard-Andersen, Regulation of cell polarity in bacteria. J. Cell Biol. 206, 7–17 (2014). 25002676 10.1083/jcb.201403136 PMC4085708

[70] D. Devenport, D. Oristian, E. Heller, E. Fuchs, Mitotic internalization of planar cell polarity proteins preserves tissue polarity. Nat. Cell Biol. 13, 893–902 (2011). 21743464 10.1038/ncb2284 PMC3149741

[71] R. Shrestha, K. A. Little, J. V. Tamayo, W. Li, D. H. Perlman, D. Devenport, Mitotic control of planar cell polarity by polo-like kinase 1. Dev. Cell 33, 522–534 (2015). 26004507 10.1016/j.devcel.2015.03.024 PMC4464975

[72] M. Tio, G. Udolph, X. Yang, W. Chia, cdc2 links the *Drosophila* cell cycle and asymmetric division machineries. Nature 409, 1063–1067 (2001). 11234018 10.1038/35059124

[73] M. M. McLellan, B. L. Aerne, J. J. Banerjee Dhoul, M. V. Holder, T. Auchynnikava, N. Tapon, Meru co-ordinates spindle orientation with cell polarity and cell cycle progression. EMBO J. 44, 2949–2975 (2025). 40169811 10.1038/s44318-025-00420-5 PMC12084343

[74] S. Z. Swartz, T. H. Tan, M. Perillo, N. Fakhri, G. M. Wessel, A. H. Wikramanayake, I. M. Cheeseman, Polarized Dishevelled dissolution and reassembly drives embryonic axis specification in sea star oocytes. Curr. Biol. 31, 5633–5641.e4 (2021). 34739818 10.1016/j.cub.2021.10.022 PMC8692449

[75] K. Ragkousi, K. Marr, S. McKinney, L. Ellington, M. C. Gibson, Cell-cycle-coupled oscillations in apical polarity and intercellular contact maintain order in embryonic epithelia. Curr. Biol. 27, 1381–1386 (2017). 28457868 10.1016/j.cub.2017.03.064

[76] G. Jeyanathan, M. M. Cao, M. Pellikka, S. Robinson, V. Ghorayeb, P. Talukder, U. Tepass, Mitotic polarity oscillation promotes epithelial tumor progression. bioRxiv 2025.02.06.636979 [Preprint] (2025). 10.1101/2025.02.06.636979.

[77] L. N. Deutz, S. Sarıkaya, D. J. Dickinson, Membrane extraction in native lipid nanodiscs reveals dynamic regulation of Cdc42 complexes during cell polarization. Biophys. J. 124, 876–890 (2023). 38006206 10.1016/j.bpj.2023.11.021 PMC11947473

[78] J. Packer, A. G. Gubieda, A. Brooks, L. N. Deutz, I. Squires, S. Ellison, C. Schneider, S. R. Naganathan, A. J. Wollman, D. J. Dickinson, Atypical protein kinase C promotes its own asymmetric localisation by phosphorylating Cdc42 in the *C. elegans* zygote. bioRxiv 2023.10.27.563985 [Preprint] (2024). 10.1101/2023.10.27.563985.

[79] F. Wirtz-Peitz, T. Nishimura, J. A. Knoblich, Linking cell cycle to asymmetric division: Aurora-A phosphorylates the Par complex to regulate Numb localization. Cell 135, 161–173 (2008). 18854163 10.1016/j.cell.2008.07.049 PMC2989779

[80] M. Das, T. Drake, D. J. Wiley, P. Buchwald, D. Vavylonis, F. Verde, Oscillatory dynamics of Cdc42 GTPase in the control of polarized growth. Science 337, 239–243 (2012). 22604726 10.1126/science.1218377 PMC3681419

[81] W. R. Holmes, J. Park, A. Levchenko, L. Edelstein-Keshet, A mathematical model coupling polarity signaling to cell adhesion explains diverse cell migration patterns. PLoS Comput. Biol. 13, e1005524 (2017). 28472054 10.1371/journal.pcbi.1005524 PMC5436877

[82] H. Meinhardt, Orientation of chemotactic cells and growth cones: Models and mechanisms. J. Cell Sci. 112, 2867–2874 (1999). 10444381 10.1242/jcs.112.17.2867

[83] H. Meinhardt, A. Gierer, Applications of a theory of biological pattern formation based on lateral inhibition. J. Cell Sci. 15, 321–346 (1974). 4859215 10.1242/jcs.15.2.321

[84] L. Plazen, J. A. Rahbani, C. M. Brown, A. Khadra, Polarity and mixed-mode oscillations may underlie different patterns of cellular migration. Sci. Rep. 13, 4223 (2023). 36918704 10.1038/s41598-023-31042-8 PMC10014943

[85] A. Jilkine, L. Edelstein-Keshet, A comparison of mathematical models for polarization of single eukaryotic cells in response to guided cues. PLoS Comput. Biol. 7, e1001121 (2011). 21552548 10.1371/journal.pcbi.1001121 PMC3084230

[86] J. P. Town, O. D. Weiner, Local negative feedback of Rac activity at the leading edge underlies a pilot pseudopod-like program for amoeboid cell guidance. PLoS Biol. 21, e3002307 (2023). 37747905 10.1371/journal.pbio.3002307 PMC10553818

[87] N. Loyer, J. Januschke, Where does asymmetry come from? Illustrating principles of polarity and asymmetry establishment in Drosophila neuroblasts. Curr. Opin. Cell Biol. 62, 70–77 (2020). 31698250 10.1016/j.ceb.2019.07.018

[88] C. H. Oon, K. E. Prehoda, Asymmetric recruitment and actin-dependent cortical flows drive the neuroblast polarity cycle. Elife 8, e45815 (2019). 31066675 10.7554/eLife.45815 PMC6524966

[89] C. H. Oon, K. E. Prehoda, Phases of cortical actomyosin dynamics coupled to the neuroblast polarity cycle. Elife 10, e66574 (2021). 34779402 10.7554/eLife.66574 PMC8641948

[90] G. P. Bell, G. C. Fletcher, R. Brain, B. J. Thompson, Aurora kinases phosphorylate Lgl to induce mitotic spindle orientation in *Drosophila epithelia*. Curr. Biol. 25, 61–68 (2015). 25484300 10.1016/j.cub.2014.10.052 PMC4291145

[91] C. A. Carvalho, S. Moreira, G. Ventura, C. E. Sunkel, E. Morais-de-Sá, Aurora A triggers Lgl cortical release during symmetric division to control planar spindle orientation. Curr. Biol. 25, 53–60 (2015). 25484294 10.1016/j.cub.2014.10.053

[92] S. Le Bras, R. Le Borgne, Epithelial cell division—Multiplying without losing touch. J. Cell Sci. 127, 5127–5137 (2014). 25344250 10.1242/jcs.151472

[93] S. Moreira, M. Osswald, G. Ventura, M. Gonçalves, C. E. Sunkel, E. Morais-de-Sá, PP1-mediated dephosphorylation of Lgl controls apical-basal polarity. Cell Rep. 26, 293–301.e7 (2019). 30625311 10.1016/j.celrep.2018.12.060

[94] M. Osswald, E. Morais-de-Sa, Dealing with apical–basal polarity and intercellular junctions: A multidimensional challenge for epithelial cell division. Curr. Opin. Cell Biol. 60, 75–83 (2019). 31153057 10.1016/j.ceb.2019.04.006

[95] K. E. Miller, P. J. Kang, H.-O. Park, Regulation of Cdc42 for polarized growth in budding yeast. Microb. Cell 7, 175–189 (2020). 32656257 10.15698/mic2020.07.722 PMC7328677

[96] K. D. Moran, H. Kang, A. V. Araujo, T. R. Zyla, K. Saito, D. Tsygankov, D. J. Lew, Cell-cycle control of cell polarity in yeast. J. Cell Biol. 218, 171–189 (2019). 30459262 10.1083/jcb.201806196 PMC6314536

[97] K. Witte, D. Strickland, M. Glotzer, Cell cycle entry triggers a switch between two modes of Cdc42 activation during yeast polarization. Elife 6, e26722 (2017). 28682236 10.7554/eLife.26722 PMC5536948

[98] U. Kadiyala, D. Sprinzak, N. A. Monk, S. E. Taylor, B. Verd, K. F. Sonnen, L. Moon, A. H. Roeder, R. Perez-Carrasco, P. Formosa-Jordan, From genes to patterns: Five key dynamical systems concepts to decode developmental regulatory mechanisms. Development 152, dev204617 (2025). 40748213 10.1242/dev.204617 PMC12377817

[99] D. J. Cislo, M. J. Delás, J. Briscoe, E. D. Siggia, Reconstructing Waddington’s landscape from data. bioRxiv 2025.08.11.669575 [Preprint] (2025). 10.1101/2025.08.11.669575. PMC12704731 41337485

[100] M. Sáez, J. Briscoe, D. A. Rand, Dynamical landscapes of cell fate decisions. Interface Focus 12, 20220002 (2022). 35860004 10.1098/rsfs.2022.0002 PMC9184965

[101] J. Rombouts, M. L. Zhao, A. Aulehla, A. Erzberger, System size and boundaries determine the patterning dynamics of attracting active particles. arXiv:2509.08533 [nlin.PS] (2025).

[102] F. Corson, E. D. Siggia, Gene-free methodology for cell fate dynamics during development. Elife 6, e30743 (2017). 29235987 10.7554/eLife.30743 PMC5771671

[103] T. Stiernagle, Maintenance of *C. elegans*. WormBook 2006, 1–11 (2006). 10.1895/wormbook.1.101.1 PMC4781397 18050451

[104] J. A. Arribere, R. T. Bell, B. X. Fu, K. L. Artiles, P. S. Hartman, A. Z. Fire, Efficient marker-free recovery of custom genetic modifications with CRISPR/Cas9 in *Caenorhabditis elegans*. Genetics 198, 837–846 (2014). 25161212 10.1534/genetics.114.169730 PMC4224173

[105] G. A. Dokshin, K. S. Ghanta, K. M. Piscopo, C. C. Mello, Robust genome editing with short single-stranded and long, partially single-stranded DNA donors in *Caenorhabditis elegans*. Genetics 210, 781–787 (2018). 30213854 10.1534/genetics.118.301532 PMC6218216

[106] R. S. Kamath, J. Ahringer, Genome-wide RNAi screening in *Caenorhabditis elegans*. Methods 30, 313–321 (2003). 12828945 10.1016/s1046-2023(03)00050-1

[107] N. W. Goehring, C. Hoege, S. W. Grill, A. A. Hyman, PAR proteins diffuse freely across the anterior–posterior boundary in polarized *C. elegans* embryos. J. Cell Biol. 193, 583–594 (2011). 21518794 10.1083/jcb.201011094 PMC3087016

[108] L. G. Edgar, B. Goldstein, “Culture and manipulation of embryonic cells” in *Methods in Cell Biology* (Elsevier, 2012), vol. 107, pp. 151–175. 10.1016/B978-0-12-394620-1.00005-9 PMC3319665 22226523

[109] C. R. Hsu, R. Xiong, K. Sugioka, In vitro reconstitution of spatial cell contact patterns with isolated *Caenorhabditis elegans* embryo blastomeres and adhesive polystyrene beads. J. Vis. Exp. 153, e60422 (2019). 10.3791/60422 31840666

[110] C. A. Shelton, B. Bowerman, Time-dependent responses to glp-1-mediated inductions in early *C. elegans* embryos. Development 122, 2043–2050 (1996). 8681785 10.1242/dev.122.7.2043

[111] M. Sandler, A. Howard, M. Zhu, A. Zhmoginov, L.-C. Chen, “Mobilenetv2: Inverted residuals and linear bottlenecks,” in *Proceedings of the IEEE Conference on Computer Vision and Pattern Recognition* (IEEE, 2018), pp. 4510–4520.

[112] N. T. L. Rodrigues, T. Bland, J. Borrego-Pinto, K. Ng, N. Hirani, Y. Gu, S. Foo, N. W. Goehring, SAIBR: a simple, platform-independent method for spectral autofluorescence correction. Development 149, dev200545 (2022). 35713287 10.1242/dev.200545 PMC9445497

[113] K. Ng, T. Bland, N. Hirani, N. W. Goehring, An analog sensitive allele permits rapid and reversible chemical inhibition of PKC-3 activity in *C. elegans*. MicroPubl. Biol. 2022, 10.17912/micropub.biology.000610 (2022). 10.17912/micropub.biology.000610 PMC9391946 35996692

[114] Y. Mori, A. Jilkine, L. Edelstein-Keshet, Wave-pinning and cell polarity from a bistable reaction-diffusion system. Biophys. J. 94, 3684–3697 (2008). 18212014 10.1529/biophysj.107.120824 PMC2292363

[115] M. P. Dalwadi, P. Pearce, Universal dynamics of biological pattern formation in spatio-temporal morphogen variations. Proc. R. Soc. London Ser. A Math. Phys. Eng. Sci. 479, 20220829 (2023).

[116] S. Blanchoud, C. Busso, F. Naef, P. Goenczy, Quantitative analysis and modeling probe polarity establishment in *C. elegans* embryos. Biophys. J. 108, 799–809 (2015). 25692585 10.1016/j.bpj.2014.12.022 PMC4336357

[117] V. Holubec, K. Kroy, S. Steffenoni, Physically consistent numerical solver for time-dependent Fokker-Planck equations. Phys. Rev. E 99, 032117 (2019). 30999402 10.1103/PhysRevE.99.032117

[118] J. R. Dormand, P. J. Prince, A family of embedded Runge-Kutta formulae. J. Comput. Appl. Math. 6, 19–26 (1980).

[119] M. Cobbaut, N. Q. McDonald, P. J. Parker, Control of atypical PKCι membrane dissociation by tyrosine phosphorylation within a PB1-C1 interdomain interface. J. Biol. Chem. 299, 104847 (2023). 37211093 10.1016/j.jbc.2023.104847 PMC10333572

[120] B. Han, K. R. Antkowiak, X. Fan, M. Rutigliano, S. P. Ryder, E. E. Griffin, Polo-like kinase couples cytoplasmic protein gradients in the *C. elegans* zygote. Curr. Biol. 28, 60–69.e8 (2018). 29276126 10.1016/j.cub.2017.11.048 PMC5763555

[121] T. B. Sells, R. Chau, J. A. Ecsedy, R. E. Gershman, K. Hoar, J. Huck, D. A. Janowick, V. J. Kadambi, P. J. LeRoy, M. Stirling, S. G. Stroud, T. J. Vos, G. S. Weatherhead, D. R. Wysong, M. Zhang, S. K. Balani, J. B. Bolen, M. G. Manfredi, C. F. Claiborne, MLN8054 and Alisertib (MLN8237): Discovery of selective oral Aurora A inhibitors. ACS Med. Chem. Lett. 6, 630–634 (2015). 26101564 10.1021/ml500409n PMC4468408

[122] S. De Henau, M. Pagès-Gallego, W.-J. Pannekoek, T. B. Dansen, Mitochondria-derived H_2_O_2_ promotes symmetry breaking of the *C. elegans* zygote. Dev. Cell 53, 263–271.e6 (2020). 32275886 10.1016/j.devcel.2020.03.008

[123] L.-E. Fielmich, R. Schmidt, D. J. Dickinson, B. Goldstein, A. Akhmanova, S. Van Den Heuvel, Optogenetic dissection of mitotic spindle positioning in vivo. Elife 7, e38198 (2018). 30109984 10.7554/eLife.38198 PMC6214656

[124] M. G. Manfredi, J. A. Ecsedy, A. Chakravarty, L. Silverman, M. Zhang, K. M. Hoar, S. G. Stroud, W. Chen, V. Shinde, J. J. Huck, Characterization of Alisertib (MLN8237), an investigational small-molecule inhibitor of aurora A kinase using novel in vivo pharmacodynamic assays. Clin. Cancer Res. 17, 7614–7624 (2011). 22016509 10.1158/1078-0432.CCR-11-1536

