# Supplementary material for: Cell cycle oscillations in a polarity network facilitate state switching by morphogenetic cues
Source: Sci Adv. 2026 May 13;12(20):eaec3379. doi: 10.1126/sciadv.aec3379 (PMC13170641; doi:10.1126/sciadv.aec3379)
Supplement: Supplementary file 1 — Supplementary Text Figs. S1 to S18 Tables S1 to S5 Legends for movies S1 to S7 References [file sciadv.aec3379_sm.pdf]

Science Advances
AAAS

Supplementary Materials for

Cell cycle oscillations in a polarity network facilitate state switching by morphogenetic cues

KangBo Ng *et al.*

Corresponding author: Nathan W. Goehring, nate.goehring@crick.ac.uk

Sci. Adv. **12**, eaec3379 (2026)
DOI: 10.1126/sciadv.aec3379

The PDF file includes:

Supplementary Text
Figs. S1 to S18
Tables S1 to S5
Legends for movies S1 to S7
References

Other Supplementary Material for this manuscript includes the following:

Movies S1 to S7

Supplemental Text

1 Constructing phase portrait of PAR behaviors

Feedback oscillations have been proposed as a mechanism to balance sensitivity and stability in cell polarity networks. To investigate whether CDK-1–coupled oscillations within the PAR network can achieve this balance, we developed a modeling framework that illustrates the system's behavior as feedback levels vary. Specifically, we constructed a phase portrait of polarity behaviors based on a prototypical reaction-diffusion model of PAR polarity (Fig. 3) [53-56, 116], which allowed us to analyze the system's dynamics as a landscape under different feedback strengths. Our approach was inspired by the work of Nandan and colleagues [57], who used a similar framework to describe the excitability and stability of the Cdc42 wave-pinning polarity model in yeast.

In the case of the Cdc42 wave-pinning model by Nandan et al. [57], the x-axis and y-axis of the graph are described by the amount of membrane-associated Cdc42 at the left of the cell (u_L) and at the right of the cell (u_R) respectively. This representation allowed all possible states of the system to be captured: high levels of homogeneous Cdc42 on the membrane would be on the top right of the graph, Cdc42 uniformly off the membrane would be at the bottom left, Cdc42 polarized towards the left of the cell would be at the bottom right, and Cdc42 polarized towards the right of the cell would be at the top left.

PAR polarity involves two polarity species, i.e. aPARs and pPARs, instead of one. Thus, to allow visualization of different polarity behaviors using this approach, we used a functional representation of the axes instead. Specifically, the x-axis and y-axis are defined by the concentration difference between aPARs and pPARs at the anterior membrane ($A_a - P_a$) and at the posterior membrane ($A_p - P_p$). This should allow us to capture all possible polarity configurations defined in previous work by Goehring et al. [53], which includes two polarized states and two unpolarized states. Here, aPAR homogenous high states would have high $A_a > P_a$ and $A_p > P_p$ and would thus be on the top right corner of the graph. pPAR homogenous high states would be at the bottom left for the same reason, as $P_a > A_a$ and $P_p > A_p$. Polarized states with aPARs high at the anterior and pPARs high at the posterior would be located at the bottom right ($A_a > P_a$ and $P_p > A_p$), while the opposite configuration of polarized state would be at the top left ($P_a > A_a$ and $A_p > P_p$).

We wanted to take advantage of this framework to analyze system sensitivity and stability due to its ability to capture a diverse range of polarity phases. In a sensitive system, cues should be able to direct polarity coherently across the landscape; in a stable system, polarized configurations should exist and be stably maintained.

1.1 A simplified ODE PAR model

A theoretical model of PAR polarization was previously described using the following set of partial differential equations (PDEs) with zero-flux Neumann boundary conditions:

$$
\begin{aligned}
\partial_t A &= D_A \partial_x^2 A + k_{on,A} A_{cyto} - k_{off,A} A - k_{AP} P^{\alpha} A \\
\partial_t P &= D_P \partial_x^2 P + k_{on,P} P_{cyto} - k_{off,P} P - k_{PA} A^{\beta} P
\end{aligned}
\tag{1}
$$

where k_{AP} and k_{PA} refers to pPAR to aPAR antagonistic feedback and aPAR to pPAR antagonistic feedback respectively, and α and β refer to non-linear antagonism terms. Assuming fast cytoplasmic diffusion and mass conservation, the equations governing the cytoplasmic pool can be described as:

$$
\begin{aligned}
A_{cyto} &= \rho_A - \psi \bar{A} \\
P_{cyto} &= \rho_P - \psi \bar{P}
\end{aligned}
\tag{2}
$$

where ρ_A and ρ_P describe total aPAR and pPAR concentrations respectively, ψ describes the surface-area-to-volume ratio, and $\bar{A}$ and $\bar{P}$ represent average aPAR and pPAR concentrations on the membrane respectively.

For convenience, we simplified this reaction-diffusion model by discretizing the system into two compartments, representing the whole of the anterior and posterior domains respectively. For instance, aPARs at the anterior half of the cell are represented as a single species A_a, and aPARs at the posterior half of the cell as A_p. For simplicity, we symmetrized the system, such that diffusion, on and off rates, and the total concentration of A and P proteins are identical. Finally, we also modified the diffusion terms accordingly, to represent exchange between the anterior and posterior compartments of the cell. Together, this gives us the following four ordinary differential equations (ODEs):

$$
\begin{aligned}
\frac{\mathrm{d}A_a}{\mathrm{d}t} &= \tilde{D}(A_p - A_a) + k_{on} A_{cyto} - k_{off} A_a - k_{AP} P_a^{\alpha} A_a \\
\frac{\mathrm{d}A_p}{\mathrm{d}t} &= \tilde{D}(A_a - A_p) + k_{on} A_{cyto} - k_{off} A_p - k_{AP} P_p^{\alpha} A_p \\
\frac{\mathrm{d}P_a}{\mathrm{d}t} &= \tilde{D}(P_p - P_a) + k_{on} P_{cyto} - k_{off} P_a - k_{PA} A_a^{\beta} P_a \\
\frac{\mathrm{d}P_p}{\mathrm{d}t} &= \tilde{D}(P_a - P_p) + k_{on} P_{cyto} - k_{off} P_p - k_{PA} A_p^{\beta} P_p
\end{aligned}
\tag{3}
$$

where $\tilde{D}$ are diffusion-like terms. Parameters are provided in Table S3.

Descriptions of the cytoplasmic pool of aPARs and pPARs are formally written as:

$$
\begin{aligned}
A_{cyto} &= \rho_A - \psi\left(\frac{A_a + A_p}{2}\right) \\
P_{cyto} &= \rho_P - \psi\left(\frac{P_a + P_p}{2}\right)
\end{aligned}
\tag{4}
$$

1.2 Phase diagram in parameter space varying antagonism terms in the ODE model

To verify that the simplified two-step discretization model exhibited qualitative features similar to the full PDE model described previously [53, 55], we calculated the parameter space of the ODE system varying the antagonism terms k_{AP} and k_{PA} as in Goehring et al. and Trong et al. (Fig. S7) [53, 54].

Homogeneous steady states were calculated analytically. First we remove the spatial component from equation (3) and (4) for simplification, to get:

$$\begin{aligned}\frac{\mathrm{d}A}{\mathrm{d}t} &= k_{on}A_{cyto} - k_{off}A - k_{AP}P^{\alpha}A \\ \frac{\mathrm{d}P}{\mathrm{d}t} &= k_{on}P_{cyto} - k_{off}P - k_{PA}A^{\beta}P \\ A_{cyto} &= \rho_A - \psi A \\ P_{cyto} &= \rho_P - \psi P\end{aligned} \tag{5}$$

In equation (5), we first solve for $\frac{\mathrm{d}P}{\mathrm{d}t} = 0$ and rearrange to get:

$$P = \frac{k_{on}\rho_P}{k_{on}\psi + k_{off} + k_{PA}A^{\beta}} \tag{6}$$

and substituted this expression of P into the first equation from (5), assuming $\frac{\mathrm{d}A}{\mathrm{d}t} = 0$, i.e.

$$0 = k_{on}A_{cyto} - k_{off}A - k_{AP}P^{\alpha}A \tag{7}$$

We then solved the values for A and P.

We identified 3 possible steady states as in Goehring et al. [53]: aPAR dominant regions ($A > P$), pPAR dominant regions ($P > A$), and regions that can support both aPAR and pPAR dominant regions as well as a third unstable steady state where $A = P$.

Regions where homogeneous aPAR or pPAR dominant regions can spontaneously polarize were further identified by performing linear stability analysis considering the following ansatz:

$$\begin{aligned}U(t) &= U_0 + \delta U(t) \\ U &\in \{A_a, A_p, P_a, P_p\}\end{aligned} \tag{8}$$

where $\delta U = (\delta A_a, \delta A_p, \delta P_a, \delta P_p)^T \mathrm{e}^{\lambda t}$, representing a small perturbation with a growth rate λ, and U_0 reflects steady state distributions of PAR proteins. Substituting this into equation (3) and linearizing, we get the following Jacobian:

$$J = \begin{pmatrix} \frac{k_{on}A\psi}{2} + k_{off}A + k_{AP}P_0^{\alpha} & \frac{k_{on}A\psi}{2} & k_{AP}\alpha {P_0}^{\alpha-1}A_0 & 0 \\ \frac{k_{on}A\psi}{2} & \frac{k_{on}A\psi}{2} + k_{off}A + k_{AP}{P_0}^{\alpha} & 0 & k_{AP}\alpha {P_0}^{\alpha-1}A_0 \\ k_{PA}\beta {A_0}^{\beta-1}P_0 & 0 & \frac{k_{on}P\psi}{2} + k_{off}P + k_{PA}{A_0}^{\beta} & \frac{k_{on}P\psi}{2} \\ 0 & k_{PA}\beta {A_0}^{\beta-1}P_0 & \frac{k_{on}P\psi}{2} & \frac{k_{on}P\psi}{2} + k_{off}P + k_{PA}{A_0}^{\beta} \end{pmatrix} \tag{9}$$

where instability occurs when the eigenvalues of J have at least one positive real part.

Finally, stably polarizable regions were numerically calculated by initializing the system with polarized distribution of aPARs and pPARs. Specifically, aPARs are initialized with two times the homogeneous steady state value at the anterior and zero at the posterior, and vice versa for pPARs. The simulation was considered capable of supporting polarity if $(A_a - P_a)$ and $(P_p - A_p)$ were both greater than 10%.

Importantly, we found the shape of the parameter space to be qualitatively similar to previous descriptions [53, 54], suggesting that the ODE simplification is reasonable.

1.3 Calculating system dynamics in the phase portrait

Having confirmed that the simplified ODE system behaves qualitatively similar to the full PDE model, we sought to calculate how the system "moves" towards the steady state points, given a position in the phase portrait. These movements were represented by the quiver arrows in the phase portrait (Fig. 3). While each point in the phase portrait is degenerate, e.g. $(A_a - P_a) = 0.5$ can be satisfied with both $A_a = 2, P_a = 1.5$ or $A_a = 0.5, P_a = 0$, we can calculate how movement takes place near steady state, providing us with information on how the system moves away from steady state initial conditions during symmetry breaking. How the system is initialized across the phase portrait is formally written as:

Initialization $(A_x - P_x)$	A_x value	P_x value
if $(A_x - P_x) > 2P_0$,	$(A_x - P_x)$	0
if $2P_0 \geq (A_x - P_x) \geq -2A_0$,	$A_0 + \frac{1}{2}(A_x - P_x)$	$P_0 - \frac{1}{2}(A_x - P_x)$
if $-2A_0 > (A_x - P_x)$,	0	-$(A_x - P_x)$

where $x \in \{a, p\}$, and $A_0 = P_0$, which represent the values of homogeneous high aPAR and pPAR steady states respectively. This formulation effectively minimizes deviations from the midpoint of the homogeneous steady states. The conditions in the first and third rows ensure that the A_x and P_x values do not fall below zero.

To ensure that this initialization is comparable for different feedback strengths, which would have different steady state values, we fixed A_0 and P_0 values for all simulations, based on simulations with $k_{AP} = k_{PA} = 0.1$. This tells us how the system would move towards the new steady state point(s) if the

feedback strength suddenly changes, providing intuition on the effects of oscillatory feedback during PAR polarization.

Initialized in this way, we next approximated how the system behaves at each point of the phase portrait by numerically simulating equations (3) and (4) for 750 seconds. The movement of the system for each point can then be estimated by simply comparing the start and end points of the simulations, with the magnitude represented as the Euclidean distance. These give rise to the directionality and length of the arrows in the phase portrait.

1.4 Estimating quasi-potential landscape

We estimated the quasi-potential landscape of the system by approximating equation (3) as stochastic differential equations (SDEs), using the Euler-Maruyama method. Formally:

$$\begin{gathered} U_x(t+\mathrm{d}t) \approx U_x(t) + (\frac{\mathrm{d}U_x}{\mathrm{d}t})\mathrm{d}t + \sigma\sqrt{\mathrm{d}t}\xi \\ U_x \in \{A_a, A_p, P_a, P_p\} \end{gathered} \tag{10}$$

where σ define the noise term and ξ is a vector of independent standard normal random variables (i.e. $\xi \sim \mathcal{N}(0,1)$).

Specifically, we performed stochastic simulations $1 * 10^7$ times for 3,000s, with a σ of 0.01, and recorded the final position of the simulation. The system is initialized as in section 1.3 at a random point across the phase portrait. We then reconstructed the quasi-potential landscape by first binning the final positions using a 2d histogram, and calculated quasi-potential (Q) as $Q = -\ln(\text{histogram density} + 1e-9)$.

1.5 Incorporating an aPAR-acting cue into the system

To incorporate a minimal aPAR-acting cue which mimics advective flows to the simplified reaction-diffusion model, we modified equation (3) as follows:

$$\begin{aligned} \frac{\mathrm{d}A_a}{\mathrm{d}t} &= \tilde{D}(A_a - A_p) + k_{on}A_{cyto} - k_{off}A_a - k_{AP}P_a^{\alpha}A_a + k_{cue}A_p \\ \frac{\mathrm{d}A_p}{\mathrm{d}t} &= \tilde{D}(A_p - A_a) + k_{on}A_{cyto} - k_{off}A_p - k_{AP}P_p^{\alpha}A_p - k_{cue}A_p \\ \frac{\mathrm{d}P_a}{\mathrm{d}t} &= \tilde{D}(P_a - P_p) + k_{on}P_{cyto} - k_{off}P_a - k_{PA}A_a^{\beta}P_a \\ \frac{\mathrm{d}P_p}{\mathrm{d}t} &= \tilde{D}(P_p - P_a) + k_{on}P_{cyto} - k_{off}P_p - k_{PA}A_p^{\beta}P_p \end{aligned} \tag{11}$$

where k_{cue} represents the strength of the cue. Note that the cue here does not act on pPARs, as symmetry breaking in *C. elegans* embryos is typically achieved by modulating the spatial activity or localization of aPARs [53, 24].

1.6 Simulating PAR polarization using the simplified model

The full PAR polarization process shown in Fig. 3 is achieved by first initiating the system as in section 1.3 with $(A_a - P_a)$ and $(A_p - P_p)$ values obtained from calculating homogeneous and polarized states. Equation (11) was then simulated for 1250s with $k_{cue} = 0.002s^{-1}$ representing the polarity establishment phase, followed by simulation of equation (3) for 1250s, representing polarity maintenance phase.

We also simulated PAR polarization with dynamically changing cues and feedback. To ensure that the cues and feedback change smoothly, we modified k_{cue}, k_{AP} and k_{PA} to depend on time, i.e. $k_{cue}(t)$, $k_{AP}(t)$ and $k_{PA}(t)$, using a tanh function. These equations can be written as:

$$\begin{aligned} k_{cue}(t) &= k_{cue}\frac{1}{2}(\tanh(\frac{t-t_{on}}{w_1}) - \tanh(\frac{t-t_{off}}{w_2})) \\ k_{fb}(t) &= k_{fb}(1-(1-\frac{1}{F})\frac{1}{2}(\tanh(\frac{t-t_{dec}}{w_3}) - \tanh(\frac{t-t_{inc}}{w_4}))) \\ fb &\in \{PA, AP\} \end{aligned} \tag{12}$$

Here, t_{on} and t_{off} define when the cue is turned on and off, t_{dec} and t_{inc} define when the feedback strength decreases and increases, w_1, w_2, w_3, w_4 defines the smoothness of the transition, and F defines the fold change in amplitude during each oscillation cycle.

1.7 Calculating transition points into the AP polarized basin

We wanted to calculate the transition point in the system toward the AP polarized basin (A high at the anterior P high at the posterior) during PAR polarization in the simplified model. This is complicated by the fact that our phase portrait projects a four-dimensional system, i.e. A_a, A_p, P_a, P_p into two, i.e. $(A_a - P_a)$, $(A_p - P_p)$. To account for this, we generated different combinations of values for A_a, A_p, P_a, P_p through a linear spacing of 50 points, ranging from 0 to $\psi\rho_U, U \in \{A, P\}$ and numerically solved for the steady state values. We then map the results and the $(A_a - P_a)$ and $(A_p - P_p)$ values back to the phase portrait. The transition point is defined by regions of space (approximated by a 2d histogram) where it is only possible to converge towards the AP polarized steady state.

1.8 Comparing results with a tractable one species polarity model

We compared our topological analysis of the PAR model with a single-species polarity model to determine whether similar results can be reproduced in a mathematically tractable framework that does not require dimensionality reduction (Fig. S9). Following Nandan et al. [57], we adopted the minimal wave-pinning model of Mori et al. [114], in which positive feedback (representing Cdc42 auto-activation) drives polarization. The model makes three key assumptions: (i) the protein interconverts between an active, membrane-bound form and an inactive, cytoplasmic form; (ii) diffusion is fast in the cytoplasm and slow on the membrane; and (iii) the total amount of protein is conserved.

The system is described by:

$$\begin{aligned}
\frac{\mathrm{d}X_a}{\mathrm{d}t} &= \tilde{D}\,(X_p - X_a) + k_{on}X_{cyto} - k_{off}X_a + \gamma\,X_{cyto}\frac{X_a^n}{K^n + X_a^n} \\
\frac{\mathrm{d}X_p}{\mathrm{d}t} &= \tilde{D}\,(X_a - X_p) + k_{on}X_{cyto} - k_{off}X_p + \gamma\,X_{cyto}\frac{X_p^n}{K^n + X_p^n}.
\end{aligned} \tag{13}$$

The well-mixed cytoplasmic pool satisfies:

$$X_{cyto} = \rho_X - \psi\,\frac{X_a + X_p}{2} \tag{14}$$

Parameters can be found in Table S4.

A localized polarity cue is modelled as an additional on-rate acting only in the anterior compartment as before [57, 114],

$$\begin{aligned}
\frac{\mathrm{d}X_a}{\mathrm{d}t} &= \tilde{D}\,(X_p - X_a) + k_{on}X_{cyto} - k_{off}X_a + \gamma\,X_{cyto}\frac{X_a^n}{K^n + X_a^n} + k_{cue}X_{cyto} \\
\frac{\mathrm{d}X_p}{\mathrm{d}t} &= \tilde{D}\,(X_a - X_p) + k_{on}X_{cyto} - k_{off}X_p + \gamma\,X_{cyto}\frac{X_p^n}{K^n + X_p^n}.
\end{aligned} \tag{15}$$

Because the model is two-dimensional, the phase-space quiver can be obtained directly from Eqs. (13)–(15), without the need for time-averaging to average out curling in higher dimensions.

The quasi-potential landscape was estimated from the Fokker–Planck equation using a modified solver built by Holubec et al. [117],

$$\frac{\partial P(X_a, X_p, t)}{\partial t} = -\frac{\partial}{\partial X_a}[f_a\,P] - \frac{\partial}{\partial X_p}[f_p\,P] + D\left(\frac{\partial^2 P}{\partial X_a^2} + \frac{\partial^2 P}{\partial X_p^2}\right), \tag{16}$$

with $f_a = \frac{\mathrm{d}X_a}{\mathrm{d}t}$ and $f_p = \frac{\mathrm{d}X_p}{\mathrm{d}t}$ from Eqs. (13) or (15), and D the (isotropic) noise strength, representing diffusing probabilities to allow stochastic state transitions. The quasi-potential is then $U = -\ln\!\left(P + 10^{-12}\right)$.

To locate the transition point, we simulated Eqs. (13)–(14) for 10^5 seconds over a grid of initial conditions, is at high feedback (γ) levels. A trajectory was classified as polarized when $X_a > 1.1\,X_p$; the boundary of this basin of attraction defines the transition point.

1.9 Simulating the full PDE model

We also corroborated our results using a full PDE model by simulating equations (1) and (2) with a custom built adaptive Runge-Kutta scheme in Python for calculating dynamics of the system [118], and using Euler's method with sufficiently small time steps if k_{AP} , k_{PA} and k_{cue} are dynamic. Here, we represented

A_a, P_a, A_p, P_p as follows:

$$U_a = \int_{0}^{L/2} U(x)\mathrm{d}x,\ U_p = \int_{L/2}^{L} U(x)\mathrm{d}x,\ U \in \{A, P\} \tag{17}$$

To incorporate the cue, equation 1 is rewritten as follows:

$$\begin{aligned}
\partial_t A &= D\partial_x^2 A + k_{on}A_{cyto} - k_{off}A - k_{AP}P^{\alpha}A + C_A(x) \\
\partial_t P &= D\partial_x^2 P + k_{on}P_{cyto} - k_{off}P - k_{PA}A^{\beta}P \\
C_A(x) &= \begin{cases} k_{cue} \int_{L/2}^{L} A(x)\mathrm{d}x, & \text{if } x < L/2 \\ -k_{cue}A(x), & \text{otherwise} \end{cases}
\end{aligned} \tag{18}$$

where $C_A(x)$ is used to approximate the way cues act in the ODE form (equation (12)), and k_{cue} simply defines the cue strength.

2 Constraining antagonism parameters to fit *C. elegans* embryos

Our simplified model suggests that oscillatory feedback enables temporal organization of either polarity-stable or cue-sensitive states, enabling polarization in various cellular contexts or with different cues. We next wanted to investigate whether this regulatory strategy could also be applied to early *C. elegans* embryos, using parameters specific to the system [53, 55]. Although most of the parameters have been previously determined, the antagonistic feedback parameters were arbitrarily chosen from regions within a parameter space that support both polarization and aPAR homogeneous states. Here, we took a fitting approach to better estimate the antagonism parameters [55].

2.1 Estimating rate parameters for the antagonism terms

To estimate the antagonism rates, we fitted k_{AP} and k_{PA} from equation (1) with data from Nocodazole and Latrunculin A treated embryos (Fig. 2) to estimate rate parameters in a purely reaction-diffusion case, as both microtubules and actomyosin cortex have been shown to stabilize polarity [22, 24]. Specifically, we used a differential evolution approach to fit the data over the course of 12 minutes, minimizing the root mean square error of the sum of differences between the experiments and simulations over time (Fig. S11). The loss function L is formally written as:

$$\mathrm{L}(k_{convA}, k_{convP}, k_{PA}, k_{AP}) = \frac{1}{T}\int_0^T \Big| \text{experiment}(t) - \text{simulation}\Big(t; k_{convA}, k_{convP}, k_{PA}, k_{AP}\Big) \Big| \,\mathrm{d}t \tag{19}$$

where k_{convA} and k_{convP} represents conversion from fluorescence intensity obtained by experimental measurements to match the simulations, which have normalized ρ_A and ρ_P values [53].

To estimate how oscillating PAR-1 and CHIN-1 membrane levels affect k_{AP}, we simply assumed that k_{AP} = membrane PAR-1 levels (Fig. S11).

2.2 Examining polarity behaviors of the fitted antagonism parameters using a phase diagram

The parameter space defined by k_{AP} and k_{PA} for the *C. elegans* model is calculated similarly to the simplified ODE model (Fig. S11). However, the Jacobian used for calculating the instable states and how the polarity states differ.

Briefly, the Jacobian used for linear stability analysis is identical to Trong et al. [54], written as:

$$J = -\begin{pmatrix} D_A k^2 + k_{off,A} + \psi k_{on,A}\delta_{k,0} + k_{AP}P_o^{\alpha} & \alpha k_{AP} A_o P_o^{\alpha-1} \\ \beta k_{PA} P_o A_o^{\beta-1} & D_P k^2 + k_{off,P} + \psi k_{on,P}\delta_{k,0} + k_{PA}A_o^{\beta} \end{pmatrix} \tag{20}$$

where $\delta_{k,0}$ represents the Kronecker-delta term.

Next, polarity regions are defined when the following conditions are satisfied: $\int_0^{0.1L} A(x)\mathrm{d}x > 1.1 \int_0^{0.1L} P(x)\mathrm{d}x$ and $\int_{0.9L}^{L} P(x)\mathrm{d}x > 1.1 \int_{0.9L}^{L} A(x)\mathrm{d}x$.

3 Constructing representative P1 and P2 models

Our modeling results from the simplified ODE system suggest that in the absence of oscillatory feedback, PAR polarization becomes compromised when initiated from either homogeneous pPAR-high states or reversed polarity states. Notably, PAR polarization observed in the P blastomeres P1 and P2 provides clear examples of each case, respectively (Fig. 4 and 5). Therefore, we sought to represent polarization in these cell types using the fitted antagonism parameters and to test the role of oscillatory feedback.

3.1 Developing an initial pPAR uniform model, roughly based on P1

PAR polarization in P1 involves the use of multiple polarity pathways (Fig. S12) [7-8]. Among these are early cleavage furrow directed flows, which advects aPARs towards the nascent cell contact during cytokinesis, leading to a corresponding accumulation of aPARs. This serves as the first symmetry breaking cue, polarizing pPARs towards the embryo posterior. As the contact site occupies roughly 40% of the embryo perimeter, we represented this in the model by incorporating an aPAR pool at the embryo anterior. While it is unclear how enriched aPARs are at the anterior quantitatively, we found that a large range of values satisfy the early pPAR polarization away from the contact. We arbitrarily chose 30% of aPAR homogenous steady state values to represent this enrichment.

Following an early enrichment of aPARs at the contact site by furrow flows, P1 cells experience a second wave of advection, beginning roughly 6-7 minutes after cell birth. To represent this second wave of flows, we modified equation (1) as follows:

$$\begin{aligned} \partial_t A &= D\partial_x^2 A + k_{on}A_{cyto} - k_{off}A - k_{AP}P^{\alpha}A + \partial_x(\upsilon A) \\ \partial_t P &= D\partial_x^2 P + k_{on}P_{cyto} - k_{off}P - k_{PA}A^{\beta}P \end{aligned} \tag{21}$$

where υ represents cortical flow velocity, identical to previous work [59]:

$$\upsilon = \frac{60-x}{74}e^{-\frac{(60-x)^2}{391}} - \frac{x}{1000}e^{-\frac{x^2}{100}} \tag{22}$$

Parameters can be found in Table S5.
Note that for simplicity, we considered these flows to act only on aPARs.

To incorporate oscillations into the model, we let k_{AP} depend on time, based on the relative PAR-1 membrane levels, i.e. $k_{AP}(t)$ = PAR-1 mem(t). We used PAR-1 membrane profiles from *par-3(-)* embryos, to isolate aPAR antagonism effects on PAR-1 membrane levels. Importantly, we rescaled the PAR-1 membrane levels over time to the cell cycle length of wildtype embryos as "P1" cells in *par-3(-)* embryos divide faster (Fig. S12).

We decided not to change the total dosage of the system (ρ_A and ρ_P), cell size (L) and surface area to volume ratio (ψ) relative to the zygote, to minimize changes to the original model.

3.2 Incorporation of WEE-1 perturbations

To represent WEE-1 inhibition in the model we shifted the PAR-1 membrane profile forwards in time to match experimental data, such that levels began to rise roughly 1.5 minutes after cell birth (Fig. S16).

3.3 Incorporation of optogenetic knocksideways of PAR-6

To represent optogenetic knocksideways of PAR-6 in the model, we let the total aPAR pool depend on time, i.e $\rho_A(t)$ (Fig. S17). We noticed that release of PAR-6 from the mitochondria following blue light release was rapid and had detectable effects within one minute. Thus, we simplified the release kinetics in the model by assuming that PAR-6 at the mitochondria is removed instantly after alleviating blue-light induced sequestration. We arbitrarily assumed that blue light-induced sequestration of PAR-6 reduces ρ_A by two thirds: the sequestration is unlikely to be complete, as we note that aPARs are still present on the membrane in the presence of blue light, but at significantly lower levels.

3.4 Developing a polarity reversal model, roughly based on P2

PAR polarization in P2 involves at least two cues: the first cue polarizes the cell in the incorrect direction towards the embryo posterior, followed by a second cue that polarizes the cell towards the embryo anterior

due to signals from the neighboring cell EMS (Fig. S18) [8-9]. This polarity reversal phenotype was particularly obvious in the PAR-2 microtubule binding mutants. It is likely that cortical flows act to also reinforce the asymmetry of P2 cells as well, but these were neglected as these flows act much later and are not well characterized.

Due to the fast-acting nature of the first cue, we suspected that it is the same furrow cue found in P1 cells. We thus represented this cue in the same way as P1 cells.

The second cue enriches pPARs at the EMS-P2 cell contact. Although it is not clear how this enrichment is achieved, it is less consistent with the direct recruitment of pPARs to the contact, as this enrichment is lost when PKC-3 activity is disrupted [56]. Instead, the phenotype is most consistent with a local reduction in PKC-3 activity at the contact. Thus, we assumed that EMS signalling, which acts through MES-1/SRC-1, causes a local reduction in k_{PA} at the cell contact, encompassing roughly 17.5% of the embryo. We incorporated this into the model by modifying equation (1) as follows:

$$\begin{aligned} \partial_t A &= D\partial_x^2 A + k_{on}A_{cyto} - k_{off}A - k_{AP}C_{EMScue}(x,t)P^{\alpha}A \\ \partial_t P &= D\partial_x^2 P + k_{on}P_{cyto} - k_{off}P - k_{PA}A^{\beta}P \end{aligned} \tag{23}$$

where C_{EMScue} defines the signaling cue that acts to locally reduce aPAR activity at the contact, formally written as:

$$C_{EMScue}(x,t) = \begin{cases} \dfrac{1}{k_{EMScue}}, & \text{if } x \leq 0.175L \text{ and } t \in [t_{EMScueon}, t_{EMScueoff}] \\ 1, & \text{otherwise} \end{cases} \tag{24}$$

where k_{EMScue} defines the reduction in antagonism strength at the contact, when the EMS signaling is active. We assume that the cue switches on 90 seconds after cell birth ($t_{EMScueon}$) and switches off 420 seconds after cell birth ($t_{EMScueoff}$) to match the observed PAR-2 distribution over the cell cycle. Notably, recent work has shown that SRC phosphorylation of PKC-3 reduces its membrane binding ability [119], which could provide a direct mechanism for reduction in this local antagonism.

As details of PAR polarization in P2 are poorly characterized, we used the same k_{AP} oscillation profile and parameters as P1 as a proof of principle, including the effects of WEE-1 inhibition. We noted that the polarity reversal phenotypes in the simulations were subtle under these conditions, matching experiments (data not shown). However, polarity reversal was clear in *par-2(MT-)* mutants, which renders PAR-2 more sensitive to PKC-3 phosphorylation. We found that when we increase the antagonism of aPAR to pPAR (k_{PA}) to reflect this, arbitrarily by 20%, we are able to clearly capture the polarity reversal phenotype.

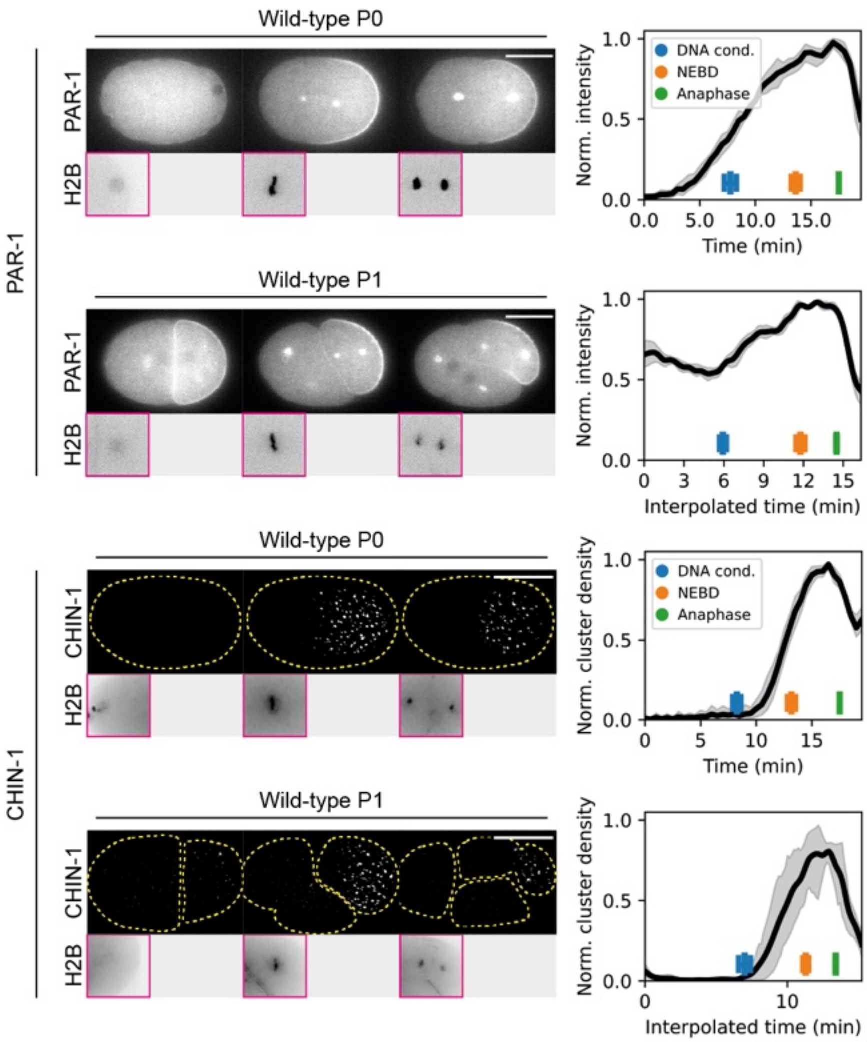

Fig. S1. PAR-1 and CHIN-1 membrane levels oscillate through the cell cycle of zygote and P1. Top, time series of midplane confocal images of embryos expressing PAR-1::GFP with mCherry::PAR-2 (not shown) and H2B::mCherry in a *par-3(+/-)* background (NWG0434, roller phenotype). Sample sizes: zygote (n=4), P1 (n=5). Bottom, time series of background subtracted cortical images of embryos expressing mNG::CHIN-1, H2B::mCherry and NMY-2::mKate2 (not shown) (NWG0528). Sample sizes: zygote (n=8), P1 (n=5). Due to the variable cell cycle length between birth of P1 to completion of cytokinesis, we normalized the cell cycle time of each embryo to the mean of all embryos, and thus temporal PAR-1 membrane profiles were interpolated to match this new normalized time (interpolated time). Measurements of PAR-1 membrane profiles and CHIN-1 cluster density were made based on the entire cell. A larger dynamic range is expected if the measurements were made based only on the posterior region. Quantifications of corresponding conditions are shown on the right. Mean and 95% confidence interval (bootstrapped) indicated. Scale bars, 20µm.

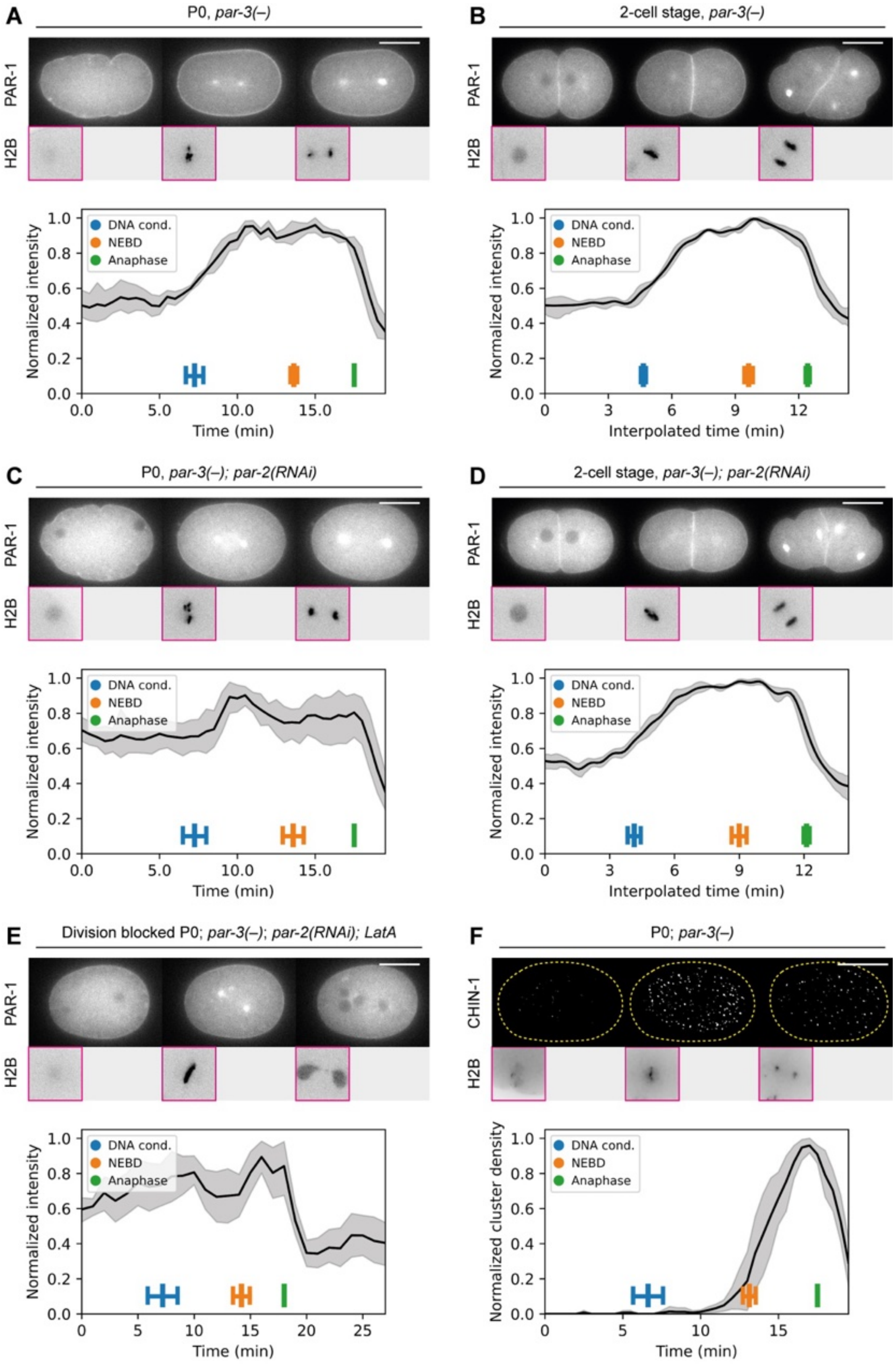

A
P0, par-3(–)
PAR-1
H2B
DNA cond.
NEBD
Anaphase
Normalized intensity
Time (min)
B
2-cell stage, par-3(–)
Interpolated time (min)
C
P0, par-3(–); par-2(RNAi)
D
2-cell stage, par-3(–); par-2(RNAi)
E
Division blocked P0; par-3(–); par-2(RNAi); LatA
F
P0; par-3(–)
CHIN-1
Normalized cluster density

Fig. S2. Additional data examining the dependencies of pPAR oscillations. (A-E) Time series of midplane confocal images of embryos expressing PAR-1::GFP with mCherry::PAR-2 (not shown) and H2B::mCherry in a *par-3(–)* background (NWG0434, roller phenotype), subject to either *ctrl(RNAi)* or *par-2(RNAi)*. The embryo on the bottom left was additionally treated with LatA to block cell division, allowing visualization of PAR-1 without confounding effects from furrow membranes during cell division. Sample sizes: zygote + *ctrl(RNAi)* (n=4), P1 + *ctrl(RNAi)* (n=5), zygote + *par-2(RNAi)* (n=6), P1 + *par-2(RNAi)* (n=5), zygote + *par-2(RNAi)* + 0.5µM LatA (n=5). **(F)** Time series of background subtracted cortical images of embryos expressing mNG::CHIN-1, H2B::mCherry, NMY-2::mKate2 (not shown) in a *par-3(–)* background (NWG0543) (n=4). Measurements of PAR-1 membrane profiles and CHIN-1 cluster density were made based on the entire cell. Quantifications of corresponding conditions are shown below each image. Mean and 95% confidence interval (bootstrapped) indicated. Scale bars, 20µm. Note *par-3(-)* background was used in all experiments to avoid the indirect influence of RNAi on aPAR feedback or polarization on pPAR membrane levels.

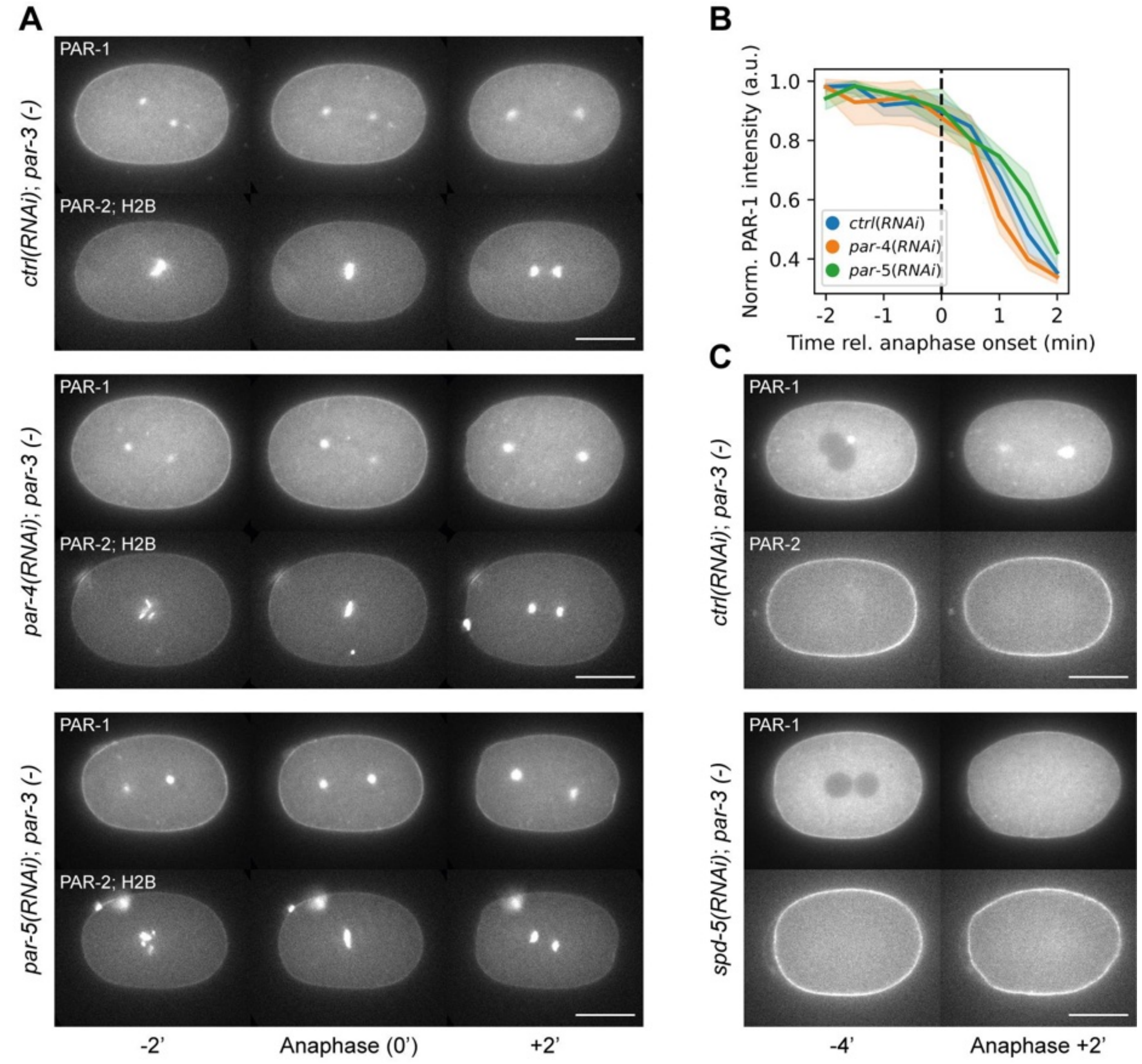

Fig. S3. Oscillations of PAR-1 membrane levels are observable when *par-4*, *par-5* and *spd-5* were depleted. (A) PAR-1 membrane oscillations do not require PAR-1 regulators PAR-4 and PAR-5, as membrane reduction during anaphase was still observed. Time series of midplane confocal images of embryos expressing PAR-1::GFP with mCherry::PAR-2 in a *par-3(–)* background (NWG0132), subject to either *ctrl(RNAi)* (n=5), *par-4(RNAi)* (n=3) or *par-5(RNAi)* (n=4). **(B)** Quantifications of average PAR-1 membrane levels across the whole embryo in conditions corresponding to **(A)**. **(C)** PAR-1 membrane reduction during anaphase was still observed when the centrosomes were disrupted, suggesting PAR-1 sequestration by the centrosomes does not play a key role. Same as in (A), comparing *ctrl(RNAi)* (n=2) and *spd-5(RNAi)* (n=2). Note *par-3(-)* background was used in all experiments to avoid the indirect influence of RNAi on aPAR feedback or polarization on pPAR membrane levels.

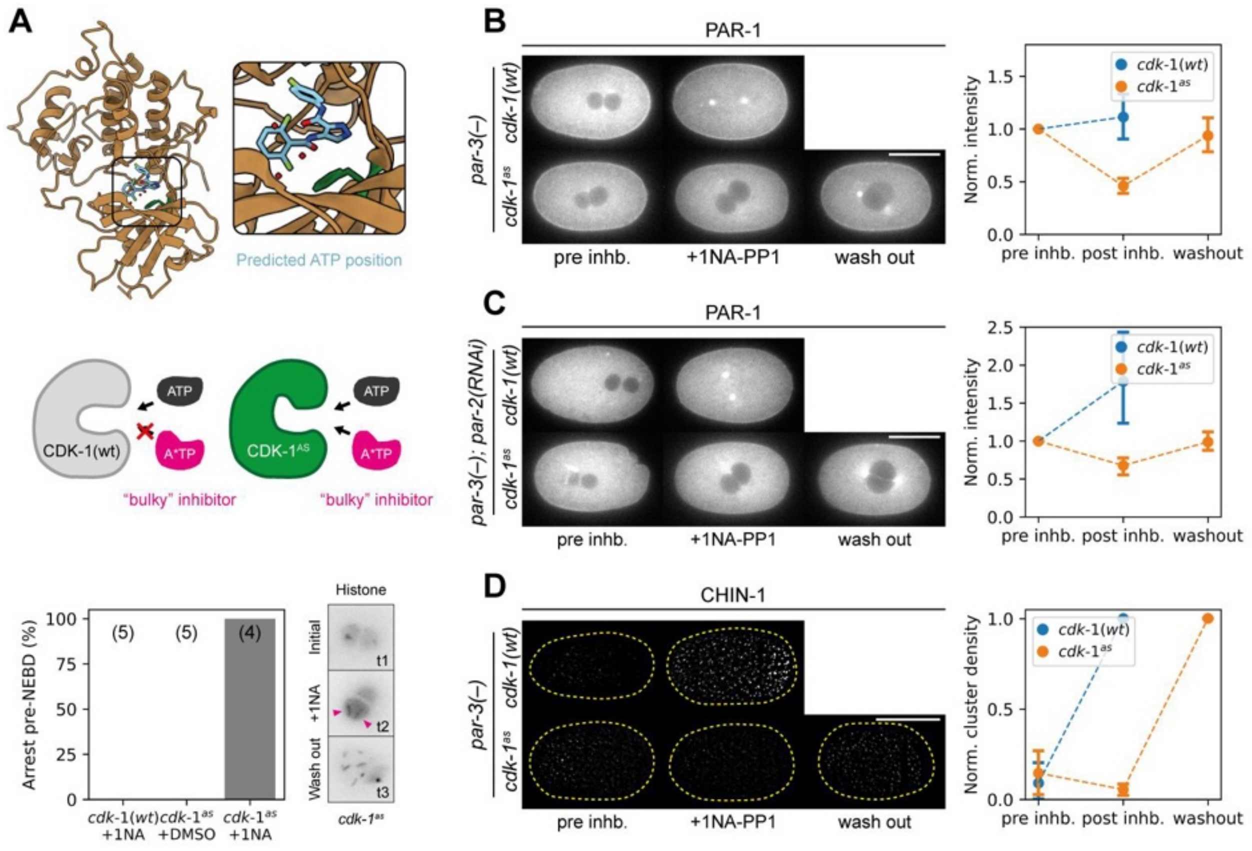

Fig. S4. Inhibition of CDK-1AS and its effect on PAR-1 and CHIN-1 membrane levels in *par-3(-)* embryos. (A) Design and testing of an analog-sensitive *cdk-1* allele (*cdk-1as*). Top, AlphaFold structure of *C. elegans* CDK-1 kinase domain superimposed with solved human CDK-1 kinase domain bound to a competitive ATP inhibitor (light blue), allows identification of the ATP binding site in *C. elegans* CDK-1. Predicted gatekeeper site in green. Middle, schematic of *cdk-1as* inhibition. Wild-type CDK-1 kinase domains (grey) readily accept ATP (dark gray) but not bulky non-hydrolysable ATP analogs (magenta), such as 1NA-PP1. CDK-1AS kinase domains (green) can accept ATP for normal functioning but also be inhibited by bulky non-hydrolysable ATP analogs, which acts as competitive inhibitors towards ATP. This is due to a mutation at the gatekeeper site to a smaller amino acid residue, which "opens up" the ATP binding pocket of the kinase. Bottom, quantification of pre-NEBD arrest when *cdk-1(wt)* or *cdk-1as* embryos were treated with DMSO or 50μM 1NA-PP1. *cdk-1(wt)* + 1NA-PP1 serves as a drug control while *cdk-1as* + DMSO serves as an allele control. Midsection confocal images of histone following CDK-1AS inhibition and wash out are shown on the bottom right, indicating prophase arrest (t2; magenta arrowheads) and reentry into the cell cycle (NEBD; t3), respectively (NWG0545; n=2). **(B)** Time series of midplane confocal images of embryos expressing PAR-1::GFP and mCherry::PAR-2 (not shown) in a *par-3(–)* with *cdk-1(wt)* (NWG0132) (n=5) or *cdk-1as* (NWG0443) (n=8) background treated with 20μM 1NA-PP1 after PNM and washed out after ~10-20 minutes. Right, quantification of normalized average PAR-1 membrane levels across the cell after treating with 1NA-PP1 and drug wash out. **(C)** Same as **(B)** but with *par-2(RNAi)*. Sample sizes: *cdk-1(wt)* (n=5), *cdk-1as* (n=8). **(D)** Same as **(B)** but quantifying normalized background subtracted CHIN-1 cluster levels. Sample sizes: *cdk-1(wt)* (NWG0467; n=4) or *cdk-1as* (NWG0474; n=5). Mean and 95% confidence interval (bootstrapped) indicated. Scale bars, 20μm.

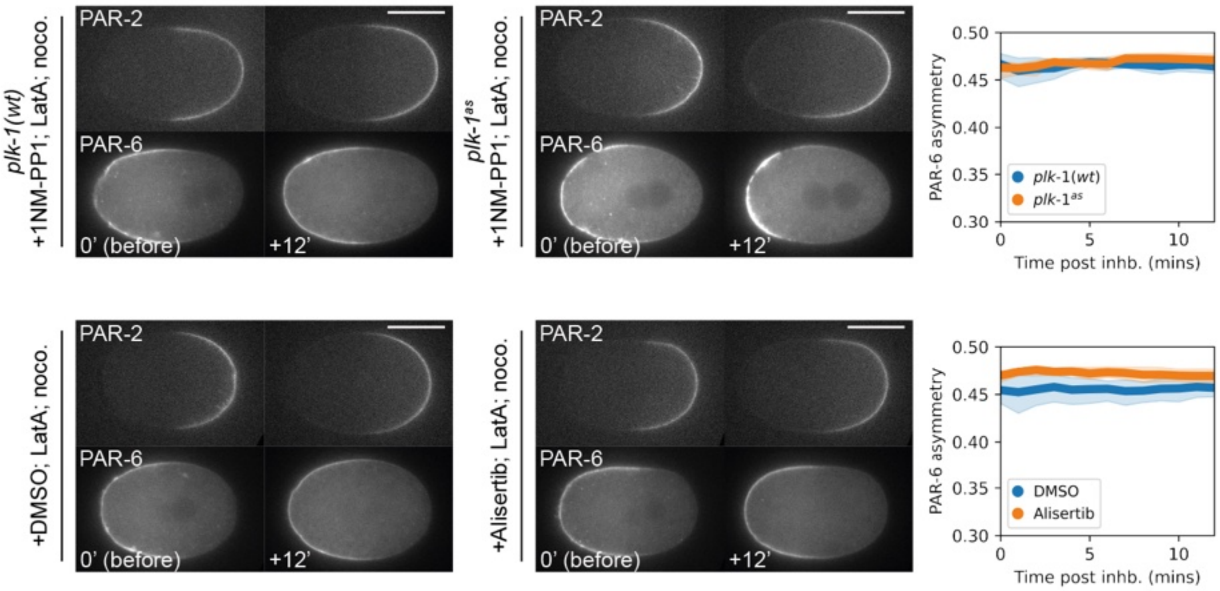

Fig. S5. Inhibition of AIR-1 and PLK-1 alongside cytoskeletal disruption does not lead to visible PAR-6 invasion into PAR-2 occupied domains. CDK-1 inhibition leads to PAR-6 invasion into PAR-2 occupied membranes and is likely not dependent on changes associated with the cytoskeleton, AIR-1 or PLK-1 activity. Midplane confocal images of embryos expressing mCherry::PAR-2 and PAR-6::mNG in different backgrounds, acutely treated with drugs after pronuclear meeting (PNM): top, *plk-1(wt)* (NWG0268; n=7) or *plk-1as* (NWG0441; n=7) background (*120*) treated with 20µM 1NM-PP1, 0.5µM LatA, and 1µg/ml nocodazole; bottom, wild-type background (NWG0268) treated with 0.5µM LatA, 1µg/ml nocodazole, and DMSO (vehicle control; n=5) or alisertib (AIR-1 inhibitor; n=7) (*121*). Open white arrowheads indicate ectopic posterior localisation of PAR-6. Right, quantification of average PAR-6 membrane levels at the embryo posterior after drug treatment. Mean and 95% confidence interval (bootstrapped) indicated. Scale bars, 20µm.

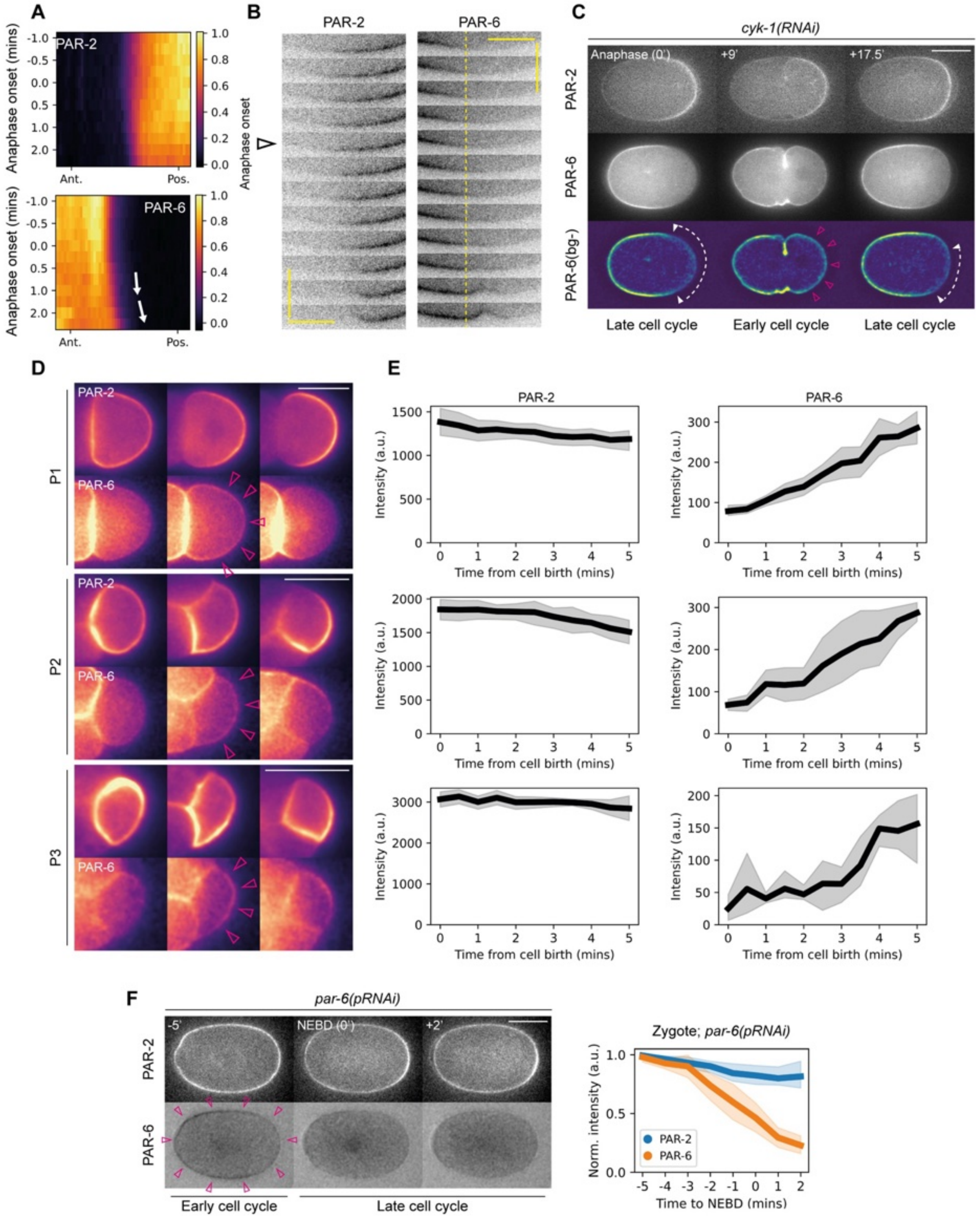

A
PAR-2
PAR-6
Anaphase onset (mins)
Ant.
Pos.
B
PAR-2
PAR-6
Anaphase onset
C
cyk-1(RNAi)
Anaphase (0')
+9'
+17.5'
PAR-2
PAR-6
PAR-6(bg-)
Late cell cycle
Early cell cycle
Late cell cycle
D
P1
P2
P3
PAR-2
PAR-6
E
PAR-2
PAR-6
Intensity (a.u.)
Time from cell birth (mins)
F
par-6(pRNAi)
-5'
NEBD (0')
+2'
PAR-2
PAR-6
Early cell cycle
Late cell cycle
Zygote; par-6(pRNAi)
Norm. intensity (a.u.)
Time to NEBD (mins)

Fig. S6. Invasion of PAR-6 into PAR-2 domains is correlated with cell cycle stage, and is observable across P blastomeres. **(A)** Quantification of average PAR-2 and PAR-6 membrane distributions during anaphase onset. White arrows indicate posterior invasion of PAR-6 during anaphase onset, as cells begin to transition to low cell cycle activity. **(B)** Corresponding kymograph corresponding to **(A)**, indicating a midplane cross section for a region close to the site of furrow formation. Vertical scale bar 30 seconds, horizontal scale bar, 10μm. Dotted yellow line indicates PAR-6 begins to invade into the posterior soon after anaphase onset (Open black arrowhead). **(C)** Time series of midsection confocal images of an embryo expressing mCherry::PAR-2 and PAR-6::mNG (NWG0268) in *cyk-1(RNAi)* conditions (n=3), which allows the zygote to progress through the cell cycle but not cell division. Arrowheads with dotted lines indicate clearance of PAR-6 from PAR-2 occupied membranes late in the cell cycle, magenta open arrowheads indicate loading of PAR-6 onto PAR-2 occupied membranes. Background subtraction of PAR-6 (PAR-6(bg-), see Methods) was performed to improve visibility of phenotype. **(D)** Time series of midsection confocal images of embryos expressing mNG::PAR-2 and PAR-6::mScarlet-I (NWG0623) in P1, P2 and P3 blastomeres (n=6). Magenta open arrowheads show aPAR loading onto the membrane even in the presence of pPARs early in the cell cycle. **(E)** Quantification of PAR membrane loading and distribution in P blastomeres. Note that we have used PAR-6::mNG (LP216) and GFP::PAR-2 (KK1273) for quantification instead - combining the use of a green fluorophore with SAIBR yielded better images. Left, average PAR-2 membrane concentration across the embryo early in the cell cycle of P1 (n=7), P2 (n=6) and P3 (n=6). Right, average PAR-6 membrane concentration across the embryo, outside of cell-cell contact sites, early in the cell cycle of P1 (n=6), P2 (n=4), and P3 (n=3). **(F)** Left, time series of midsection confocal images of an embryo expressing mCherry::PAR-2 and PAR-6::mNG (NWG0268) in *par-6* partial RNAi (*par-6(pRNAi))* conditions (n=4). Note that PAR-6 overlaps with PAR-2 (magenta open arrowhead) until right before NEBD, where PAR-6 membrane levels begin to decrease. Scale bar, 20μm. Right, quantification of PAR-2 and PAR-6 membrane levels for the corresponding conditions. Mean and 95% confidence interval (bootstrapped) indicated. Scale bars, 20μm.

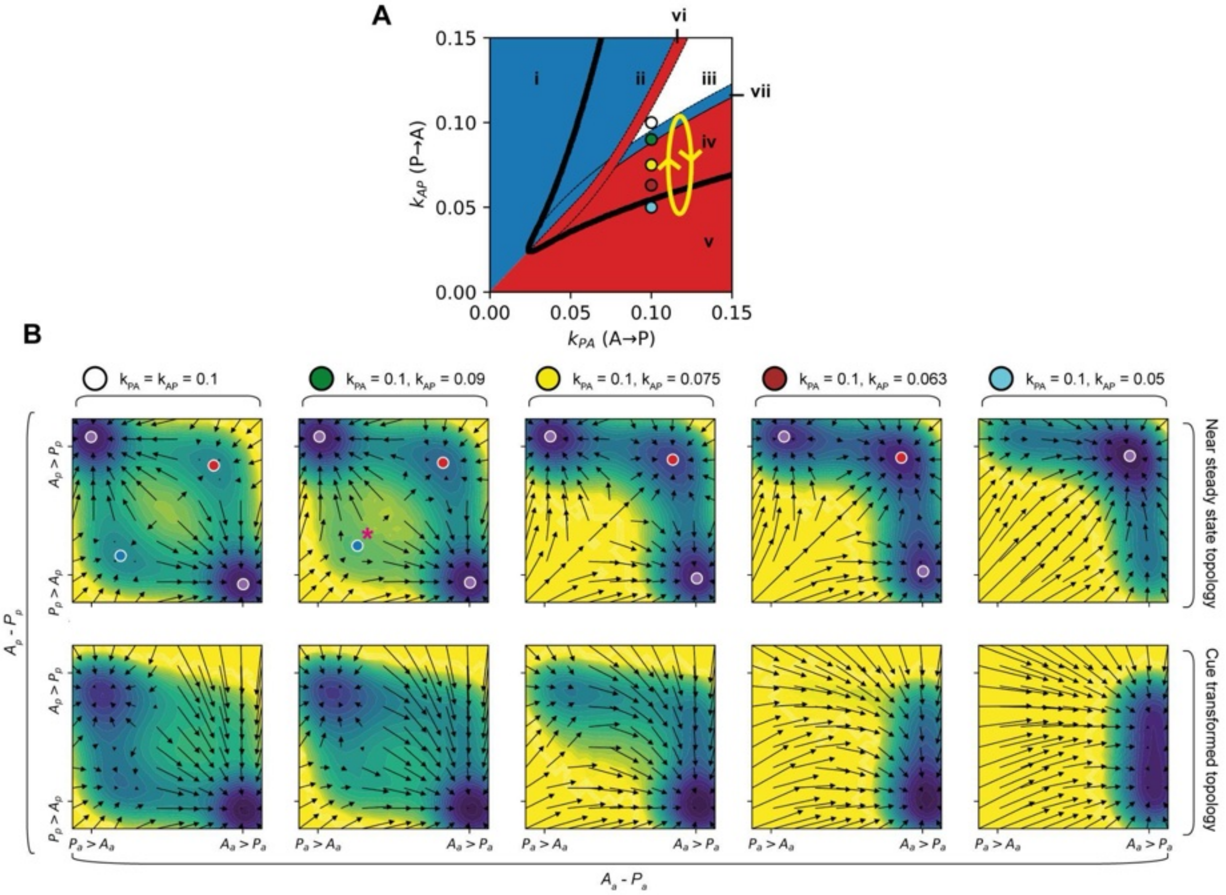

Fig. S7. Landscape of a simplified 2-step discretized PAR model with oscillating k_{AP} (P→A) feedback. (A) Parameter-space topology of a 2-step discretized PAR model (see Methods). Blue region (i, ii) supports pPAR dominant homogeneous states. The red region (iv, v) supports aPAR dominant homogenous states. White region (iii) supports both aPAR and pPAR dominant homogenous states, depending on the initial state. Dotted red region (vi) indicates region that is unstable and can undergo spontaneous symmetry breaking if beginning from aPAR initial states. Dotted blue region (vii) indicates the same, but beginning from pPAR initial states. Solid black lines indicate regions permissible to stable polarization (ii, iii, iv, vi, vii). This topology is similar to previously described works (*53*, *54*). Colored circular points with black outlines indicate points that were sampled and further examined using a concentration difference landscape in **(B)**, representing what the system experiences when it undergoes k_{AP} (P→A) oscillations. **(B)** Examination of system behavior at changing k_{AP} levels using a phase plane of concentration differences, between aPAR and pPAR at the anterior (A_a - P_a) for the x-axis, and between aPAR and pPAR at the posterior (A_p - P_p) for the y-axis. Top, near steady state topology of the system (see Methods). Dotted circles represent steady state points for either polarized states (purple), homogenous aPAR high states (red) or homogenous pPAR high states (blue). Magenta asterisk represents an unstable steady state. Bottom, topology of the system after transformation with an aPAR acting cue.

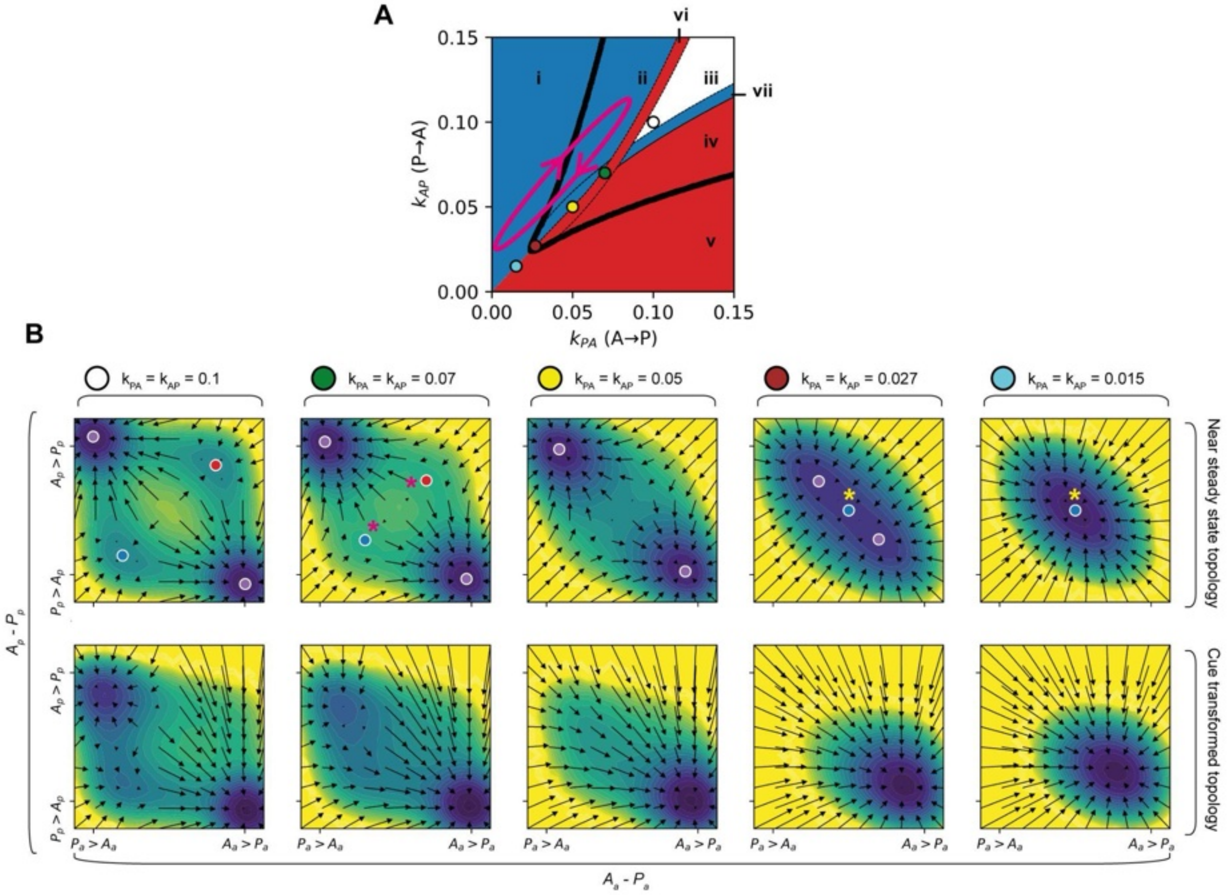

Fig. S8. Landscape of a simplified 2-step discretized PAR model, oscillating both *kAP* (P→A) and *kPA* (A→P) feedback simultaneously. (A-B) Similar to Fig. **S7**, but for oscillating both *kAP* and *kPA* simultaneously. Magenta asterisk represents unstable steady states. Yellow asterisk represents the stable point of the system which is homogenous high for both pPARs and pPARs, with both species overlapping on the membrane.

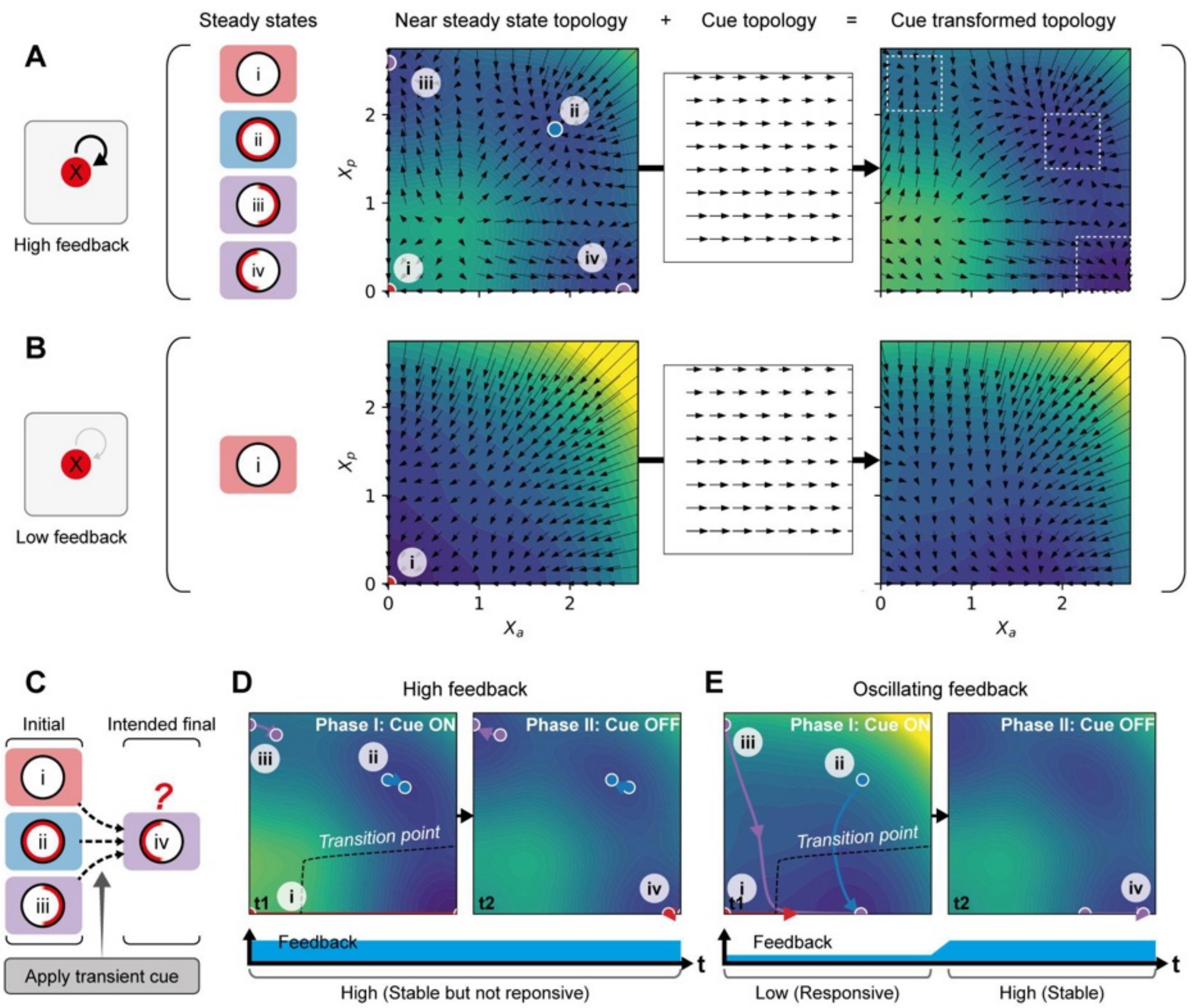
A
B
C
D
E
Steady states
Near steady state topology
+
Cue topology
=
Cue transformed topology
High feedback
Low feedback
i
ii
iii
iv
X_p
X_a
Initial
Intended final
?
Apply transient cue
High feedback
Phase I: Cue ON
Phase II: Cue OFF
Transition point
t1
t2
Feedback
t
High (Stable but not reponsive)
Oscillating feedback
Low (Responsive)
High (Stable)

Fig. S9. Oscillating feedback facilitates robust, cue-responsive polarization from diverse initial states in a mathematically tractable one species polarity model. (A) Left, schematic showing a simplified one-species polarity model based on the wave-pinning model developed by Mori et al. (*114*). Here, rather than using double negative feedback between aPARs and pPARs to polarize, the system relies on positive feedback within the polarity species *X* for the local recruitment of *X* from the cytoplasm by existing membrane-associated *X*. Within specific parameter ranges, as noted in Trong et al. (*54*), 4 possible steady states exist as with the PAR model, (i) uniform *X* low, (ii) uniform *X* high, (iii) polarised with *X* high at posterior but low at anterior, and (iv) polarised with *X* high at anterior but low at posterior. These states can all be captured in a phase space varying the concentration of *X* in the anterior, X_a, or X in the posterior, X_p. Cue is modelled as a local increase in on rate at the anterior of the cell, similar to previous work (*57*, *114*). Quivers represent movement across the system state space, and the colors represent the quasi-potential of the system, calculated using the Fokker-Planck equation (see Modelling Supplement). **(B)** Same as **(A),** but for low levels of positive feedback. **(C)** Schematic illustrating the full simulation of polarization related to **(D)**, intending to polarize systems from state i, ii and iii to state iv using the same cue described above. **(D)** Oscillatory feedback allows stable yet adaptable polarization from all system states in the landscape. Left, in the constant high feedback condition, only points around state i are able to respond reliably to cues and move beyond the transition point for polarization towards state iv. Right, in the oscillatory feedback condition, temporary low feedback facilitates cue-responsiveness towards the transition point for all initial states, before increasing feedback locks in the stable polarized state. Dotted black lines represent the transition point of the system, converging towards the basin of attraction representing state iv.

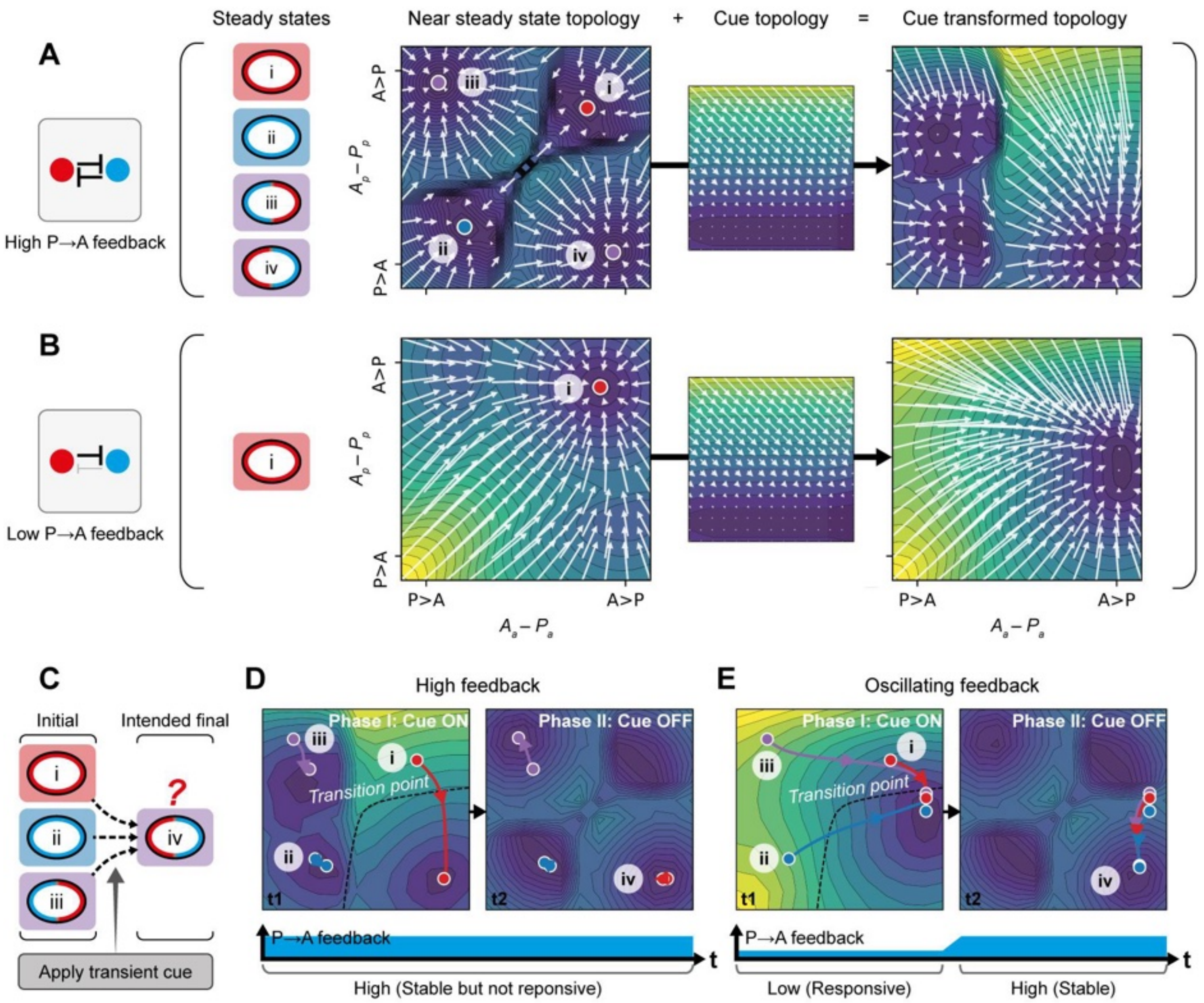

Fig. S10. Oscillating feedback facilitates robust, cue-responsive polarization from diverse initial states in a full PDE PAR model. (A-E) Identical to Figure 3 but using a system described with partial differential equations instead. Note that the colors of the contours here do not represent the quasi-potential of the system but indicate the velocity of the arrows instead.

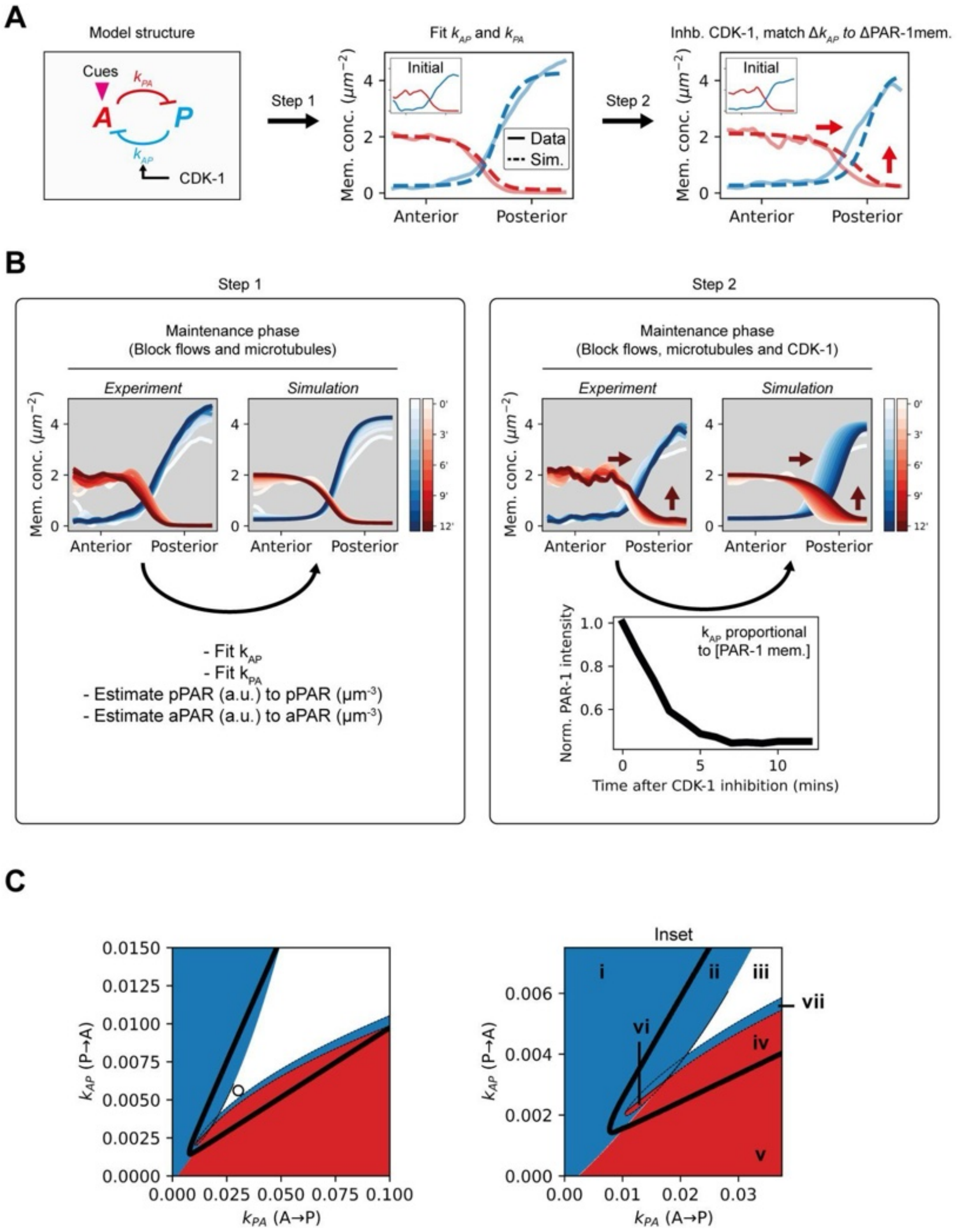
A
Model structure
Cues
A
P
CDK-1
Step 1
Fit k_{AP} and k_{PA}
Initial
Data
Sim.
Mem. conc. (μm^{-2})
Anterior
Posterior
Step 2
Inhb. CDK-1, match Δk_{AP} to ΔPAR-1mem.
B
Step 1
Maintenance phase
(Block flows and microtubules)
Experiment
Simulation
- Fit k_{AP}
- Fit k_{PA}
- Estimate pPAR (a.u.) to pPAR (μm^{-3})
- Estimate aPAR (a.u.) to aPAR (μm^{-3})
Step 2
Maintenance phase
(Block flows, microtubules and CDK-1)
k_{AP} proportional to [PAR-1 mem.]
Norm. PAR-1 intensity
Time after CDK-1 inhibition (mins)
C
Inset
k_{AP} (P→A)
k_{PA} (A→P)
i
ii
iii
iv
v
vi
vii

Fig. S11. Estimation of *C. elegans* k_{AP} (P→A) and k_{PA} (A→P) feedback via fitting with experiments. (A) Schematic illustrating the strategy used for estimating dynamic antagonistic feedback rates in changing CDK-1 activity. Step 1, antagonistic feedback was first obtained by fitting a simplified model structure with measured diffusion/on/off rates (see Supplemental Text) to data roughly around pronuclear centration, when CDK-1 activity is thought to be high. Step 2, estimating how pPAR to aPAR antagonistic feedback (k_{AP}) changes during CDK-1 inhibition. We approximated changing k_{AP} activity as changes in PAR-1 membrane levels following CDK-1 inhibition and saw that the PAR-2 and PAR-6 membrane profiles fit well with the simulations. **(B)** Comparison of simulations and data when fitting Step 1 and Step 2 shown in **(A)**. Changing colors of membrane line profiles reflect time relative to drug addiction. **(C)** Parameter-space topology of fitted parameters of the PAR model. Blue region (i, ii) supports pPAR dominant homogeneous states. Red region (iv, v) supports aPAR dominant homogenous states. White region (iii) supports both aPAR and pPAR dominant homogenous states, depending on the initial state. Dotted red region (vi) indicates region that can undergo spontaneous symmetry breaking if beginning from aPAR initial states. Dotted blue region (vii) indicates the same, but beginning from pPAR initial states. Solid black lines indicate regions permissible to stable polarization. White dotted circle represents where the fitted parameters lie.

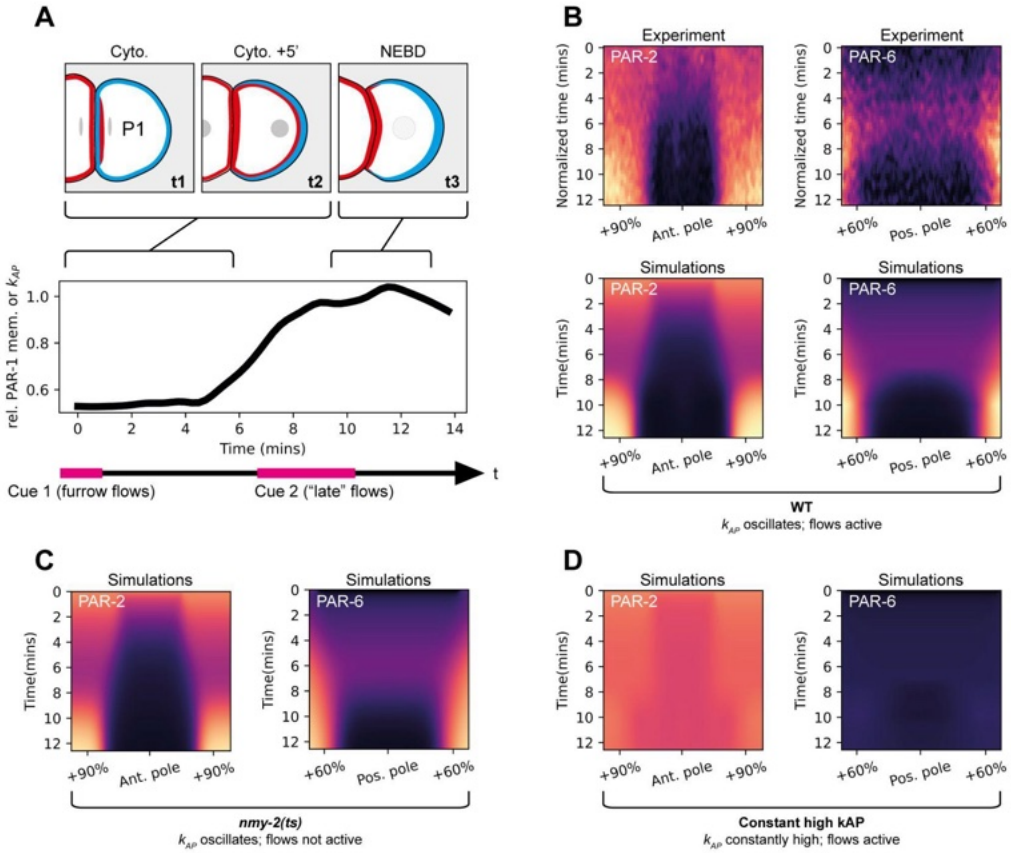

Fig. S12. Simulations of a modified PAR model representing P1 polarization. (A) Top, schematic illustrating P1 polarization pattern through the cell cycle. Bottom, estimated k_{AP} used for simulations, which is defined by relative PAR-1 membrane levels through the cell cycle. **(B)** Top, average spatiotemporal profiles of PAR-2 and PAR-6 during P1 polarization of wild-type embryos, data from Ng et al., 2023 (*8*). Bottom, simulations of a P1-specific model closely resemble the experimental data. **(C)** Simulations of a P1-specific model shows that "late" flows observed in P1 are not required for polarization, matching previous experiments (*7*, *8*). **(D)** Simulations of a P1-specific model shows that oscillatory feedback in P1 is important for polarization, as a system with constant high feedback cannot polarize.

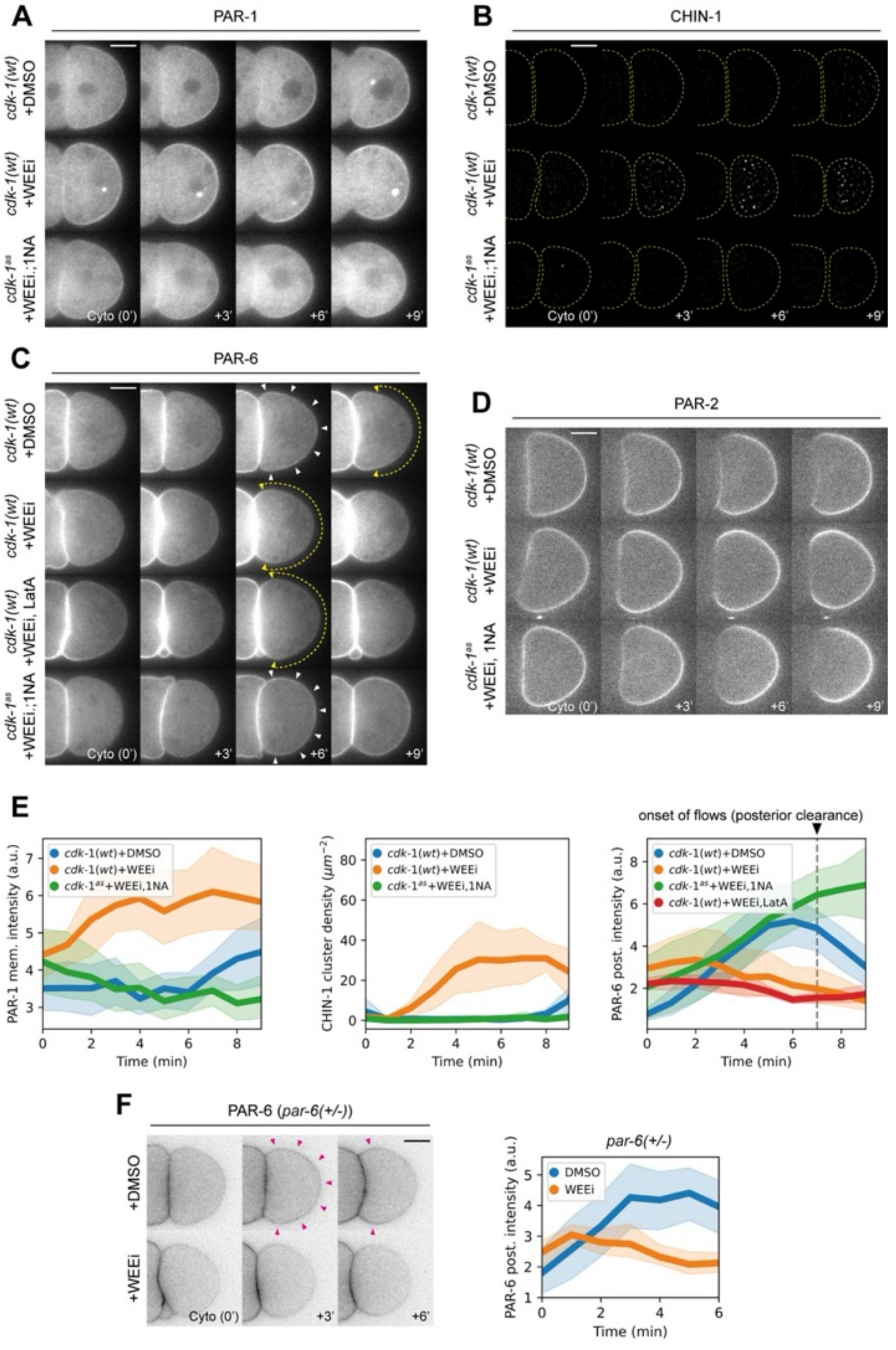

A
PAR-1
cdk-1(wt) +DMSO
cdk-1(wt) +WEEi
cdk-1as +WEEi;1NA
Cyto (0')
+3'
+6'
+9'
B
CHIN-1
C
PAR-6
cdk-1(wt) +WEEi, LatA
D
PAR-2
E
PAR-1 mem. intensity (a.u.)
CHIN-1 cluster density (μm⁻²)
PAR-6 post. intensity (a.u.)
Time (min)
onset of flows (posterior clearance)
cdk-1(wt)+DMSO
cdk-1(wt)+WEEi
cdk-1as+WEEi,1NA
cdk-1(wt)+WEEi,LatA
F
PAR-6 (par-6(+/-))
+DMSO
+WEEi
par-6(+/-)
DMSO
WEEi

Fig. S13. Effects of WEE-1 inhibition on PAR proteins during P1 polarization. (A) WEE-1 inhibition accelerates PAR-1 membrane loading. Time series of midplane confocal images of embryos expressing PAR-1::GFP and mCherry::PAR-2 (not shown) in either a *cdk-1(wt)* (NWG0332) or *cdk-1as* (NWG0566) background, acutely treated with DMSO, 20µM WEEi (PD0166825) or 20µM 1NA-PP1. Sample sizes: *cdk-1(wt)* + DMSO (n=5), *cdk-1(wt)* + WEEi (n=6), *cdk-1as* + WEEi + 1NA-PP1 (n=8). **(B)** WEE-1 inhibition accelerates CHIN-1 membrane loading. Time series of background subtracted CHIN-1 cortical images in embryos expressing mNG::CHIN-1 in either a *cdk-1(wt)* (NWG0451) or *cdk-1as* (NWG518) background, acutely treated with DMSO, 20µM WEEi or 20µM 1NA-PP1. Sample sizes: *cdk-1(wt)* + DMSO (n=5), *cdk-1(wt)* + WEEi (n=6), *cdk-1as* + WEEi + 1NA-PP1 (n=4). **(C)** WEE-1 inhibition reduces PAR-6 membrane loading onto the posterior membrane. Same setup as (A) but for PAR-6. Embryos expressing PAR-6::mNG were used, in either a *cdk-1(wt)* (LP216) or *cdk-1as* (NWG0559) background, acutely treated with DMSO, 20µM WEEi, 20µM 1NA-PP1 or 0.5 µM LatA. Sample sizes: *cdk-1(wt)* + DMSO (n=7), *cdk-1(wt)* + WEEi (n=8), *cdk-1(wt)* + WEEi + LatA (n=4), or *cdk-1as* + WEEi + 1NA-PP1 (n=5). **(D)** WEE-1 inhibition has modest effects on PAR-2 polarization. Same experiments as (A) but showing mCherry::PAR-2 instead. **(E)** Quantification of average membrane levels for conditions corresponding to **(A-C)**. Note that for PAR-6, only the posterior membrane outside of the contact site was considered for quantification. **(F)** Left, a time series of midsection confocal images of embryos expressing PAR-6::mNG in *par-6* heterozygous conditions (*par-6::mNG/–*) (NWG0141 x LP216), acutely treated with either DMSO (n=10) or 20µM WEEi (n=8). Magenta arrowheads indicate significantly higher levels of PAR-6 posterior membrane loading in DMSO treated embryos compared to WEEi treated embryos. Right, the corresponding quantification of average PAR-6 membrane levels at the posterior region of P1, outside of the contact site, after cell birth. Mean and 95% confidence interval (bootstrapped) indicated. Scale bars, 10µm.

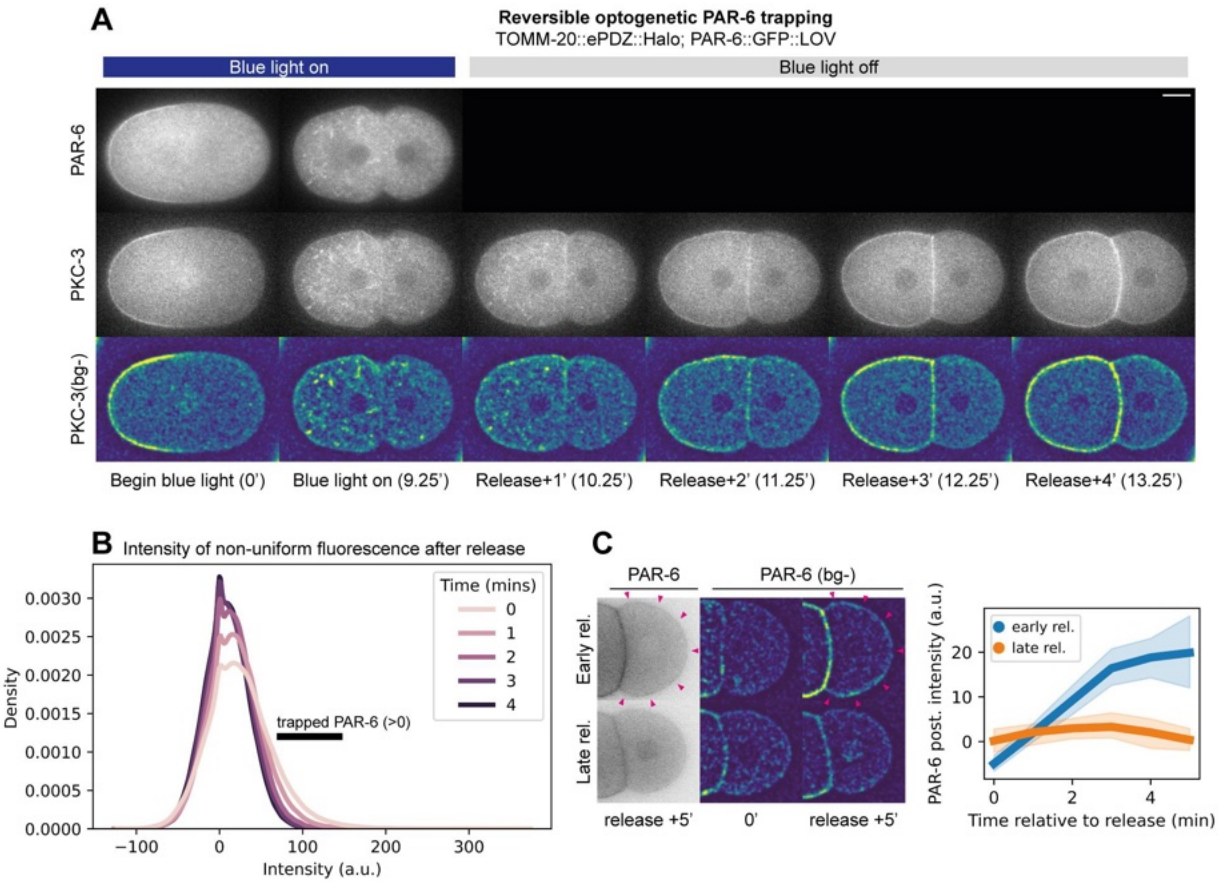

Fig. S14. Dynamics of reversible optogenetic knocksideways of PAR-6. (A) Time series of midplane confocal images of a representative embryo expressing PAR-6::GFP::LOV, TOMM-20::ePDZ::Halo and mScarlet-I::PKC-3 (NWG0630) subjected to blue light illumination and removal. Note that upon blue light illumination, PAR-6 membrane levels reduce and become sequestered into cytoplasmic structures (presumably mitochondria). This process is reversible by relieving blue light illumination. **(B)** Quantification of PAR-6 release dynamics following removal of blue-light illumination. A histogram of the fluorescence intensity distribution in the AB cytoplasm after background subtraction is shown. Uniformly distributed PAR-6::GFP::LOV that is not sequestered in mitochondria is treated as background and thus exhibits a fluorescence distribution centered around 0. In contrast, PAR-6 that has been sequestered on mitochondria appears as puncta, distinct from cytoplasmic PAR-6, and yields fluorescence intensities greater than 0. Within approximately 2 minutes, the fluorescence distribution collapses to one centered around 0, indicating that PAR-6 is released from mitochondria rapidly. **(C)** Left, a time series of midsection confocal images of embryos expressing PAR-6::GFP::LOV, TOMM-20::ePDZ::Halo and mScarlet-I::PKC-3 (NWG0630), with mitochondria-trapped PAR-6 release early (~3mins) (n=6) or late (~7.5mins) (n=6) after cell birth. Magenta arrowheads indicate significantly higher levels of PAR-6 posterior membrane loading in early release (~3 mins after cell birth) embryos compared to late release (~7 mins after cell birth) embryos. Right, the corresponding quantification of average PAR-6 membrane levels at the posterior region of P1, outside of the contact site, after cell birth.

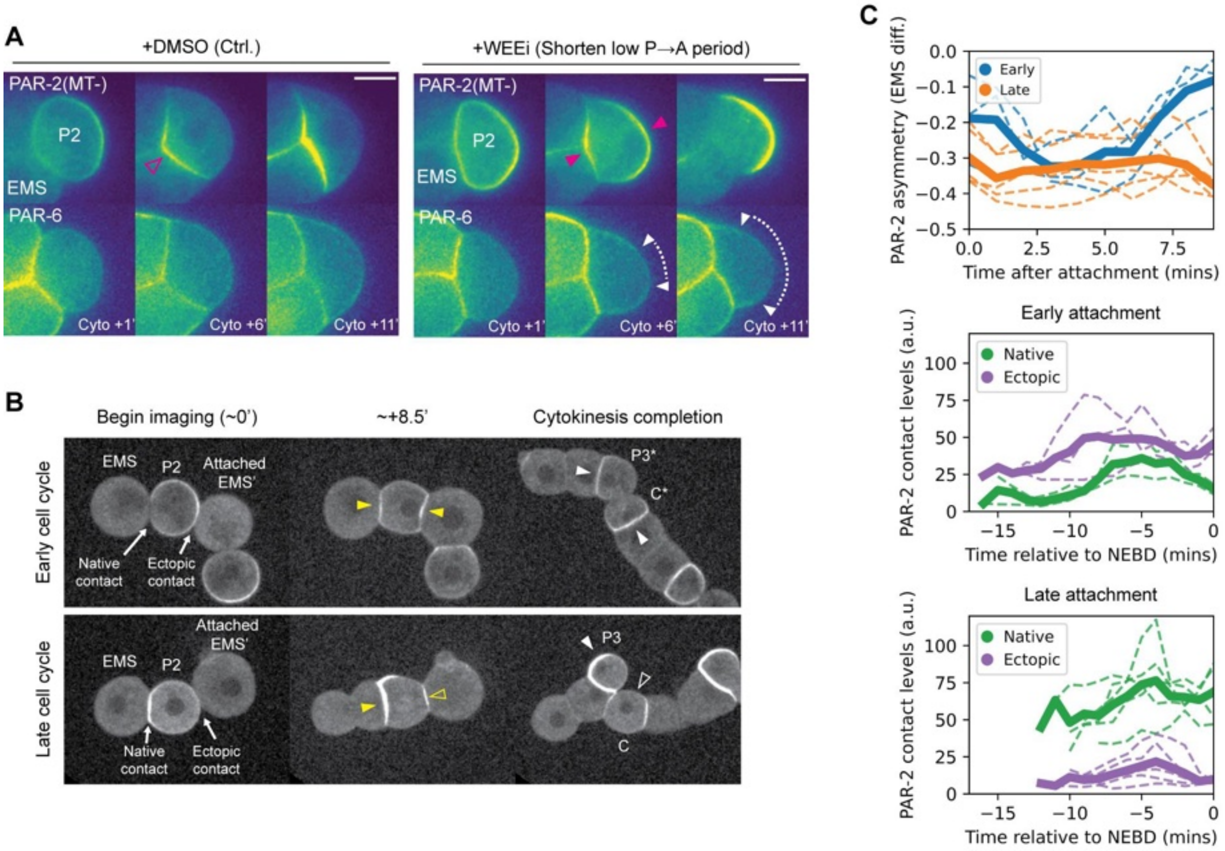

Fig. S15. Additional information on cue-sensitivity of P2 to EMS signalling when decoupled with wild-type cell cycle progression. (A) Schematic showing PAR-2(MT-) and PAR-6 behavior when P2 blastomeres were treated with the WEE-1 inhibitor. Same samples as shown in Fig. **5C**, but showing PAR-6::mScarlet-I as well. Magenta arrowheads indicate differences in the number of polarity domains in DMSO or WEEi treated embryos, when PAR-2 is recruited to the contact, presumably by the onset of signaling from EMS. White arrowheads with dotted lines indicate posterior PAR-6 clearance by the ectopic domain in WEEi treated conditions. **(B)** Uncropped images of embryo attachment experiments traced until after cell division. Yellow closed arrowheads indicate the presence of PAR-2(MT-) domains, while yellow open arrowheads indicate a second smaller PAR-2(MT-) domain. Note that PAR-2 asymmetry is greater between the P2 daughter cells (P3 and C) of late attachment embryos than early. **(C)** Quantification of conditions corresponding to **(C)**. Mean (bold lines) and results of individual experiments (dotted lines) shown. Scale bars, 20μm.

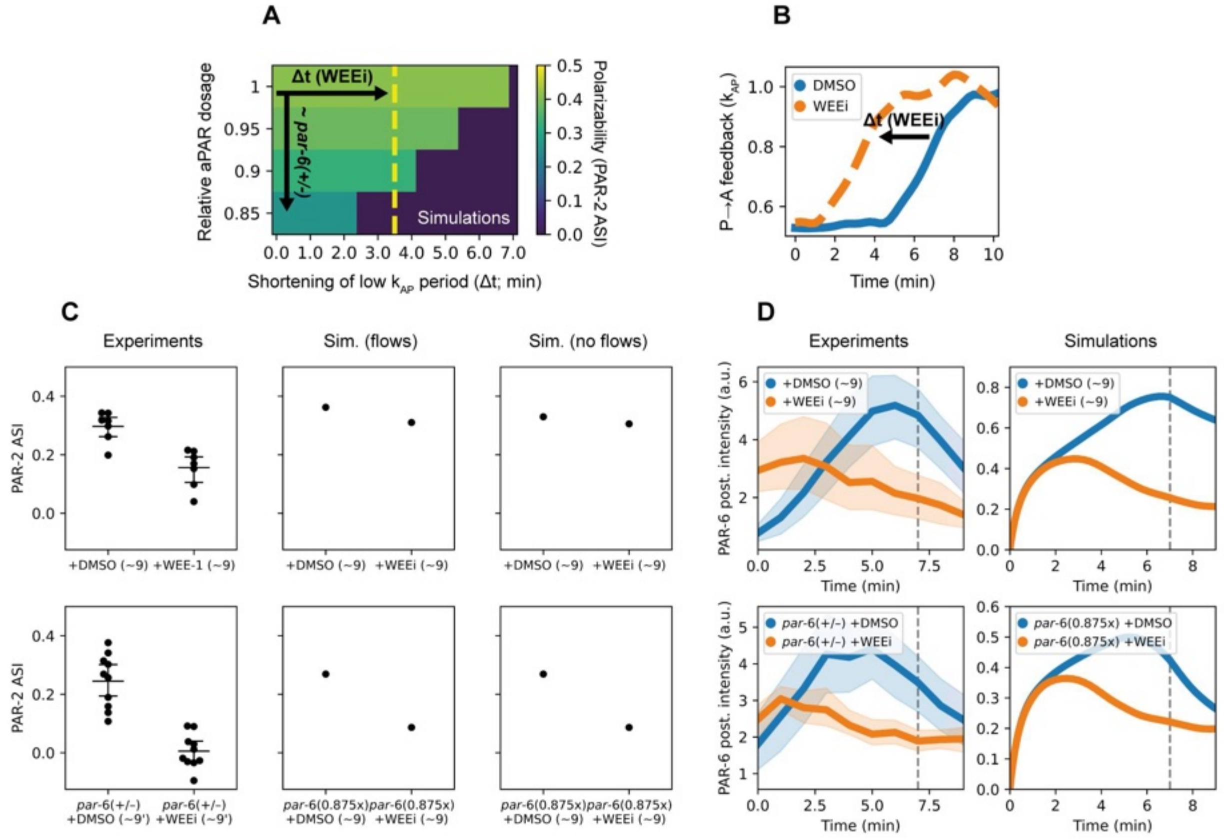

Fig. S16. Simulation outcomes resemble P1 polarization results from WEE-1 inhibition experiments. (A) Simulations of P1 polarizability when the period of low k_{AP} is shortened and in changing aPAR levels. Yellow dotted line indicates the estimated shortening of the low k_{AP} period when P1 blastomeres are treated with a WEE-1 inhibitor (PD0166825 or simply WEEi; see right and Fig. **S13**). **(B)** Predicted dynamics of pPAR to aPAR antagonistic feedback when WEE-1 is inhibited compared to wild type (see Fig. **S13**). **(C)** A comparison between experimental results and theory for PAR-2 asymmetry (ASI) ~9 mins after cytokinesis completion/ birth of cell, when WEE-1 is inhibited. The simulations were performed in the presence or absence of "late" flows described in Fig. **S12A**. We chose to compare PAR-6 heterozygotes with 0.875x total dosage in the models, as we noted that polarity was already disrupted at this level of dosage when combined with a shortened low feedback period **(A)**. **(D)** A comparison between experimental results and theory for PAR-6 membrane levels following WEE-1 inhibition. Dotted lines indicate onset of "late" flows, which advects aPARs towards the embryo anterior. Mean and 95% confidence interval (bootstrapped) indicated.

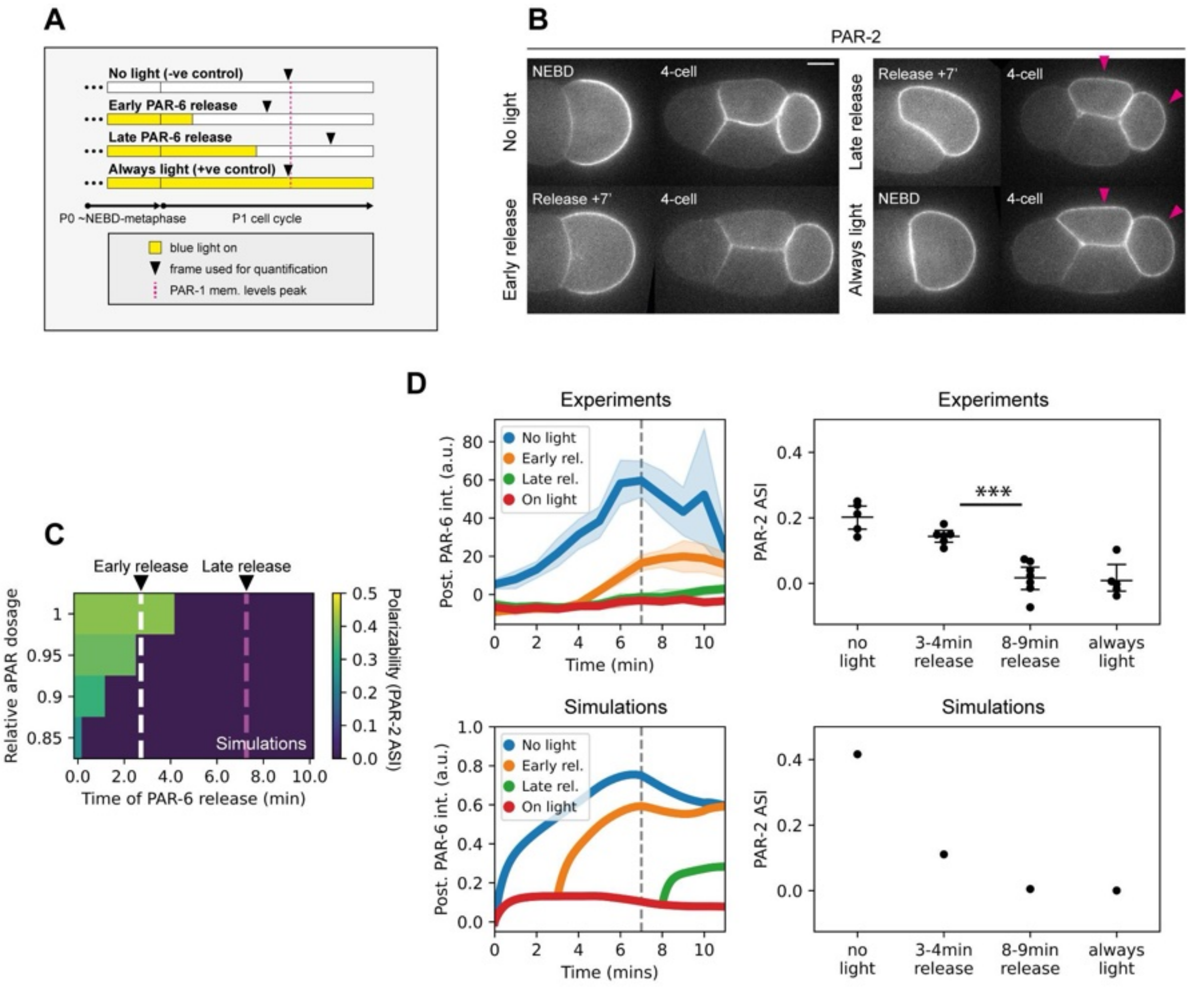

Fig. S17. Simulation outcomes resemble P1 polarization results from optogenetic knock-sideways experiments. (A) Schematic timeline for optogenetic knock-sideways experiments for PAR-6. Embryos either never or always illuminated with blue light serve as negative and positive controls respectively. **(B)** Left, time series of midsection confocal images of 2- and 4-cell stage embryos expressing PAR-6::GFP::LOV, TOMM-20::ePDZ::Halo and mScarlet-I::PAR-2 (NWG0597) (*122*, *123*) subjected to experimental conditions corresponding to (C). Sample sizes: no light (n=5), early release (n=6), late release (n=7), always light (n=5). Magenta arrowheads indicate symmetric PAR-2 inheritance in 4-cell stage embryos. Right, quantifications of PAR-2 asymmetry index (ASI) for the corresponding conditions. Early and late release confocal images are reproduced from Fig. **4G** to facilitate comparison with the negative (no light) and positive controls (always light). **(C)** Top, simulations predict that P1 polarization should be sensitive to activation of polarity at different times, which can be achieved by "releasing" aPARs at different times. Bottom, simulations of P1 polarizability at different times of aPAR release and in changing aPAR levels. White and magenta dotted lines indicate release times which allow polarization or do not, respectively, for wild-type aPAR levels, which is the timings we chose for experimentation. **(D)** Left, comparison of posterior PAR-6 membrane profiles in P1 (outside of contact region) between simulations and experimental results. Dotted lines indicate onset of "late" flows, which advects aPARs towards the embryo anterior. Right, comparison of PAR-2 asymmetry (ASI) between experimental results and theory.

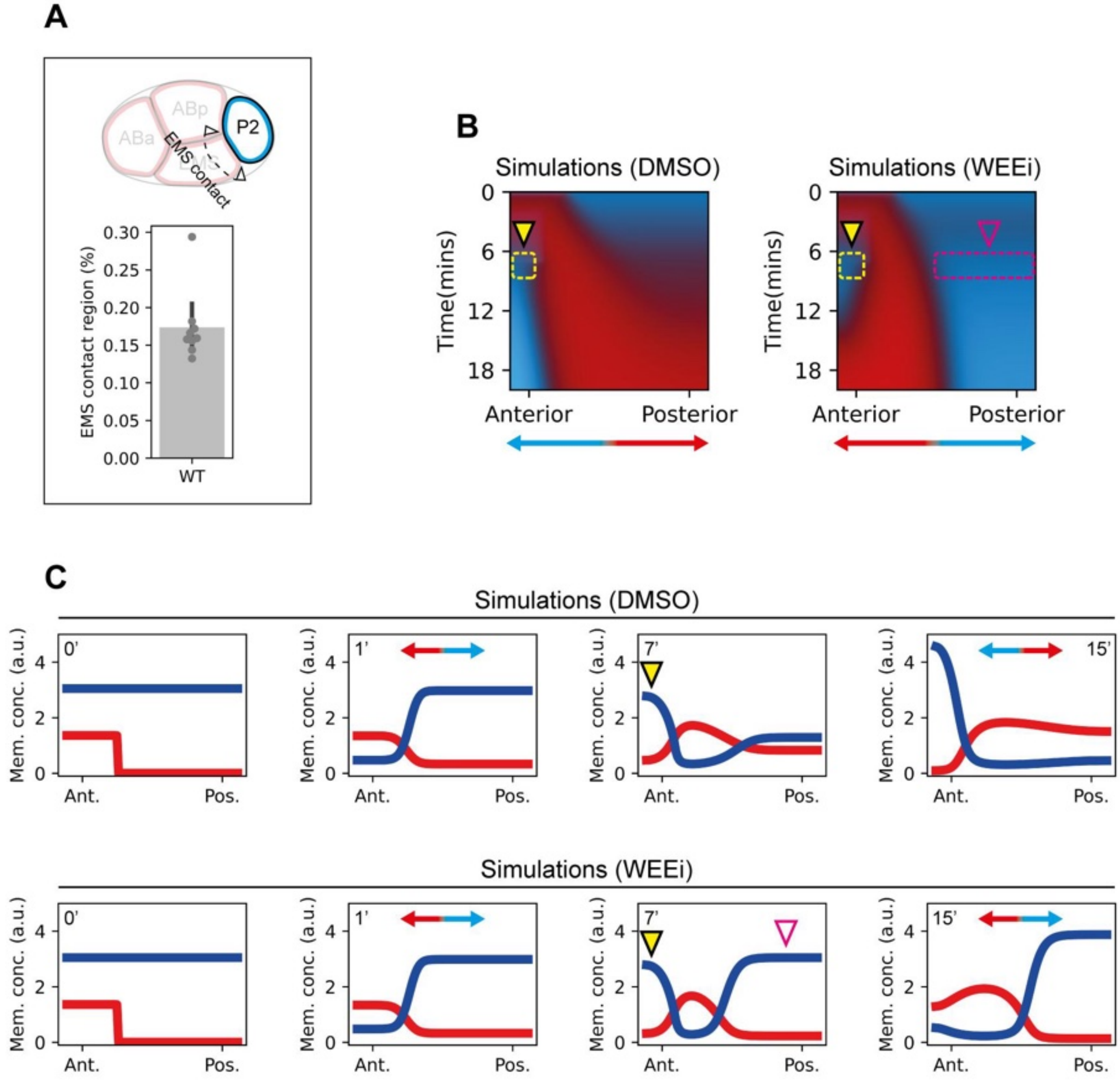

Fig. S18. Simulation outcomes resemble P2 polarity reversal results from WEE-1 inhibition experiments. (A) Quantification of the fraction of EMS-P2 contact region, which is used to then simulate the fractional domain size of the EMS signalling cue. **(B)** Spatiotemporal membrane profiles of PAR-2(MT-) simulations in P2, subjected to DMSO or WEEi, which changes when pPAR to aPAR antagonistic feedback levels increase. PAR-2(MT-) mutation is captured by increasing pPAR to aPAR antagonistic feedback rates. Yellow arrowhead indicates the "correct" developmental cue originating from MES-1/SRC-1 signaling from EMS, magenta open arrowhead indicates ectopic polarity domain formed by furrow accumulation of PAR-3 at EMS contact. The blue arrow indicates orientation of the final pPAR domain. **(C)** Same as in **(B)** but showing membrane profiles of pPARs (blue) and aPARs (red) at indicated time points. The double-sided arrow graded from red to blue indicates the orientation of the polarity axis.

Table S1. Regents and Resources

REAGENT or RESOURCE	SOURCE	IDENTIFIER
Bacteria		
E. coli: OP50: E. coli B, uracil auxotroph	CGC	WB Strain: OP50
E. coli: HT115(DE3): F-, mcrA, mcrB, IN(rrnD-rrnE)1, rnc14:: Tn10(DE3 lysogen: lavUV5 promoter-T7 polymerase).	CGC	WB Strain: HT115(DE3)
Chemicals, Peptides, and Recombinant Proteins		
Chemically defined lipid concentrate	ThermoFisher	11905031
PP1 Analog, 1NA-PP1	Merck	529579
PP1 Analog II, 1NM-PP1	Merck	529581
Nocodazole	Merck	M1404
Latrunculin A	Enzo	BML-T119-0100
PD0166285		
Alisertib (*124*)	Selleckchem	1028486-01-2
Alt-R™ S.p. Cas9 Nuclease V3	IDT	1081058
Alt-R® CRISPR-Cas9 tracrRNA	IDT	1072532
Experimental Models: Organisms/Strains		
C. elegans: EGD334: *egxSi100 II; plk-1(egx3[C52V, L115G]); unc-119(ed3) III*	Han et al. (*120*)	
C. elegans: KK1254: *par-2(it315[mCherry::par-2]) III*	Ken Kempheus	WB Strain: KK1254
C. elegans: KK1273: *par-2(it328[GFP::par-2]) III*	CGC/Ken Kempheus	WB Strain: KK1273
C. elegans: LP216: *par-6(cp45[par-6::mNeonGreen::3xFlag + LoxP unc-119(+) LoxP]) I; unc-119(ed3) III*	Dickinson et al. 2017 (*28*)	WB Strain: LP216
C. elegans: LP637: *par-2(cp329[mNG::par-2]) III*	Dickinson et al. 2017 (*28*)	WB Strain: LP637
C. elegans: N2: wild type	CGC	WB Strain: N2
C. elegans: NWG0042: *par-2(it315[mCherry::par-2]) III; par-1(it324[par-1::gfp::par-1 exon 11a]) V*	This paper	
C. elegans: NWG0132: *par-2(it315[mCherry::par-2]) III; lon-1(e185) par-3(it71) /qC1[dpy-19(e1259) glp-1(q339) qIs26] III; par-1(it324[par-1::gfp::par-1 exon 11a]) V*	This paper	
C. elegans: NWG0141: *par-6(tm1425)/In(ile-1 Y18D10A.2 In(dnj-27 dkf-1)) [unc-75(Pmyo-2::Venus)]) I*	Rodrigues et al. 2024 (*11*)	
C. elegans: NWG0192: *par-2(crk30[par-2(R183-5A)::gfp]*KK1273)*	Ng et al. 2023 *(8)*	
C. elegans: NWG0268: *par-6(cp45[par-6::mNeonGreen::3xFlag + LoxP unc-119(+) LoxP]) I; par-2(it315[mCherry::par-2]) III; unc-119(ed3) III?*	Ng et al. 2023 *(8)*	WB Strain: NWG0268
C. elegans: NWG0323: *par-6(tm1425)/In(ile-1 Y18D10A.2 In(dnj-27 dkf-1)) [unc-75(Pmyo-2::Venus)]) I; par-2(it328[GFP::par-2]) III*	Rodrigues et al. 2024 (*11*)	
C. elegans: NWG0332: *par-2 (it315[mCherry::par-2]) III; par-1(ax4206) V*	Ng et al. 2023 (*8*)	
C. elegans: NWG0365: *cdk-1(crk101[cdk-1(F98A)]) III*	This paper	
C. elegans: NWG0434: *par-2(it315[mCherry::par-2]) III; lon-1(e185) par-3(it71)/ qC1[dpy-19(e1259) glp-1(q339) qIs26] III; unc-119(ed3) III (?); itIs37 IV; stIs10226 (?); par-1(it324[par-1::gfp::par-1 exon 11a]) V*	This paper	
C. elegans: NWG0441: *par-6(cp45[par-6::mNeonGreen::3xFlag + LoxP unc-119(+) LoxP]) I; par-2(it315[mCherry::par-2]) III; plk-1(egx3[C52V,*	This paper	

L115G]) III; unc-119(ed3) III?		
C. elegans: NWG0443: *par-6(cp45[par-6::mNeonGreen::3xFlag + LoxP unc-119(+) LoxP]) I; par-2(it315[mCherry::par-2]) III; cdk-1(crk101[cdk-1(F98A)]); unc-119(ed3) III?*	This paper	
C. elegans: NWG0451: *chin-1(crk146[mNG::CHIN-1]) III*	This paper	
C. elegans: NWG0455: *chin-1(crk146[mNG::CHIN-1]) III; unc-119(ed3) III (?); itIs37 IV; stIs10226 (?)*	This paper	
C. elegans: NWG0467: chin-1(crk146[mNG::CHIN-1]) III; lon-1(e185) par-3(it71)/ qC1[dpy-19(e1259) glp-1(q339) qIs26] III	This paper	
C. elegans: NWG0468: *chin-1(crk146[mNG::CHIN-1]) III; lon-1(e185) par-3(it71)/ qC1[dpy-19(e1259) glp-1(q339) qIs26] III; unc-119(ed3) III (?); itIs37 IV; stIs10226 (?)*	This paper	
C. elegans: NWG0471: *par-2(it315[mCherry::par-2]) III; lon-1(e185) par-3(it71) cdk-1(crk160[cdk-1(F98A)])/ qC1[dpy-19(e1259) glp-1(q339) qIs26] III; par-1(it324[par-1::gfp::par-1 exon 11a]) V*	This paper	
C. elegans: NWG0474: *chin-1(crk146[mNG::CHIN-1]) III; lon-1(e185) par-3(it71) cdk-1(crk160[cdk-1(F98A)])/ qC1[dpy-19(e1259) glp-1(q339) qIs26] III*	This paper	
C. elegans: NWG0509: *nmy-2(crk181[nmy-2(L981P)]) I; par-6(cp45[par-6::mNeonGreen::3xFlag + LoxP unc-119(+) LoxP]) I; par-2(it315[mCherry::par-2]) III; unc-119(ed3) III?*	This paper	
C. elegans: NWG0518: *chin-1(crk146[mNG::CHIN-1]) III; cdk-1(crk101[cdk-1(F98A)]) III*	This paper	
C. elegans: NWG0520: *par-2(it315[mCherry::par-2]) III; cdk-1(crk101[cdk-1(F98A)]) III; par-1(it324[par-1::gfp::par-1 exon 11a]) V*	This paper	
C. elegans: NWG0528: *nmy-2(cp69[nmy-2::mkate2 + LoxP]) I; chin-1(crk146[mNG::CHIN-1]) III; unc-119(ed3) III (?); itIs37 IV; stIs10226 (?)*	This paper	
C. elegans: NWG0543: *nmy-2(cp69[nmy-2::mkate2 + LoxP]) I; chin-1(crk146[mNG::CHIN-1]) III; lon-1(e185) par-3(it71)/ qC1[dpy-19(e1259) glp-1(q339) qIs26] III; unc-119(ed3) III (?); itIs37 IV; stIs10226 (?)*	This paper	
C. elegans: NWG0545: *chin-1(crk146[mNG::CHIN-1]) III; cdk-1(crk101[cdk-1(F98A)]) III; unc-119(ed3) III (?); itIs37 IV; stIs10226 (?)*	This paper	
C. elegans: NWG0559: *par-6(cp45[par-6::mNeonGreen::3xFlag + LoxP unc-119(+) LoxP]) I; cdk-1(crk101[cdk-1(F98A)]) III; unc-119(ed3) III?*	This paper	
C. elegans: NWG0567: *par-2(it315[mCherry::par-2]) III; cdk-1(crk101[cdk-1(F98A)]) III; par-1(ax4206) V*	This paper	
C. elegans: NWG0584: *par-6(he322 [par-6::gfp(smu-1 introns)::glo-lov]) I; utdSi44[mex-5p::tomm-20::glo-epdz::glo-halo::tbb2(3'UTR)] II*	This paper	
C. elegans: NWG0597: *par-6 (he322 [par-6::gfp(smu-1 introns)::glo-lov])/ In(ile-1 Y18D10A.2 In(dnj-27 dkf-1)) [unc-75(Pmyo-2::Venus)]) I; utdSi44 [mex-5p::tomm-20::glo-epdz::glo-halo::tbb2(3'UTR)] II; par-2(djd7 [mSC::PAR-2]) III*	This paper	
C. elegans: NWG0623: *par-6(djd4 [PAR-6::mScarlet-I::Myc]) I; par-2(cp329[mNG-C1^PAR-2]) III*	This paper	
C. elegans: NWG0629: *par-6 (he322 [par-6::gfp(smu-1 introns)::glo-lov]) I; mSc::aPKC pkc-3(djd15[mSc::Myc::aPKC]) II; utdSi44 [mex-5p::tomm-20::glo-epdz::glo-halo::tbb2(3'UTR)] II*	This paper	
C. elegans: NWG0639: *par-2(crk30[par-2(R183-5A)::gfp]*KK1273)/ sC1(s2023) [dpy-1(s2170) umnIs21] III; par-6(djd4[PAR-6::mScarlet-I::Myc]) I*	This paper	
C. elegans: SV2109: *par-6(he322 [par-6::gfp(smu-1 introns)::glo-lov]) I; RuIs57(Ppie-1::Tub::GFP) V*	Fielmich et al. 2018 (*123*)	
C. elegans: TBD298: *utdSi44[mex-5p::tomm-20::glo-epdz::glo-halo::tbb2(3'UTR)] II; unc-119(ed3) III; utdSi43[mex5p::PH::glo-mtagbfp2::co-lov::tbb-2(3'UTR)] V*	De Henau et al. 2020 (*122*)	
C. elegans: UTX11: *par-6(djd4 [PAR-6::mScarlet-I::Myc]) I*	Dan Dickinson	
C. elegans: UTX31: *par-2(djd7 [mSC::PAR-2]) III*	Dan Dickinson	
Oligonucleotides		
CDK-1(F98A) sgRNA #1:	IDT DNA	

5' – /AltR1/rCrG rGrUrC rArUrU rArUrG rCrArG rGrArG rArArC rGrUrU rUrUrA rGrArG rCrUrA rUrGrC rU/AltR2/ – 3'		
CDK-1(F98A) sgRNA #2:<br>5' – /AltR1/rArU rCrGrU rUrUrC rArArG rUrCrG rArArA rGrArC rGrUrU rUrUrA rGrArG rCrUrA rUrGrC rU/AltR2/ – 3'	IDT DNA	
CDK-1(F98A) repair template (**PstI restriction site**):<br>5'– ATAGGGTGTT CCATCAACGG CTGTGCGAGA GATCAGCTTG CTCAAAGAGC TGCAGCATCC GAATGTTGTT GGATTGGAAG CGGTCATTAT GCAGGAGAAC CGACTTTTCC TGATCGCCGA ATTCTTGTCT TTCGACTTGA AACGATACAT GGATCAGTTG GGAAAAGATG AATACCTTCC GCTCGA –3'	IDT DNA	
CDK-1(F98A) FWD ODN genotyping primer:<br>5'–CAACAATCCTTCTCAGCGCG–3'	IDT DNA	
CDK-1(F98A) REV ODN genotyping primer:<br>5'–GGTGTGCCGAGAACTCTGAA–3'	IDT DNA	
NMY-2(L981P) sgRNA #1:<br>5' – /AltR1/rUrU rUrCrG rArGrC rArArC rArArU rUrUrC rUrGrA rGrUrU rUrUrA rGrArG rCrUrA rUrGrC rU/AltR2/ – 3'	IDT DNA	
NMY-2(L981P) sgRNA #2:<br>5' – /AltR1/rUrU rCrArA rUrUrG rArArU rCrUrC rGrGrU rUrGrA rGrUrU rUrUrA rGrArG rCrUrA rUrGrC rU/AltR2/ – 3'	IDT DNA	
NMY-2(L981P) repair template (**MspI restriction site**):<br>5'– CGAAAATTGA CGGAGATGGT TAGACATCTC GAAGAGAATC TTGAAGATGA AGAAAGAAGC AGACAGAAAT TGTTGCCTGA AAAAAATTCA ATTGAATCCC GGTTGAAAGA ACTGGAAGCA CAAGGACTCG AGCTTGAAGA TTCTGGAAAC AAG –3'	IDT DNA	
NMY-2(L981P) FWD ODN genotyping primer:<br>5'–ATGAGACTGCGTGAATGGCA–3'	IDT DNA	
NMY-2(L981P) REV ODN genotyping primer:<br>5'–GACTCTTTGCGCATCAGCTG–3'	IDT DNA	
mNG::CHIN-1 sgRNA #1:<br>5' – mU*mU*mU* rGrCrA rGrGrU rArUrG rGrArA rGrArC rGrArG rUrUrU rUrArG rArGrC rUrArG rArArA rUrArG rCrArA rGrUrU rArArA rArUrA rArGrG rCrUrA rGrUrC rCrGrU rUrArU rCrArA rCrUrU rGrArA rArArA rGrUrG rGrCrA rCrCrG rArGrU rCrGrG rUrGrC mU*mU*mU* rU – 3'	IDT DNA	
mNG::CHIN-1 sgRNA #2:<br>5' – mU*mU*mG* rCrArG rGrUrA rUrGrG rArArG rArCrG rArCrG rUrUrU rUrArG rArGrC rUrArG rArArA rUrArG rCrArA rGrUrU rArArA rArUrA rArGrG rCrUrA rGrUrC rCrGrU rUrArU rCrArA rCrUrU rGrArA rArArA rGrUrG rGrCrA rCrCrG rArGrU rCrGrG rUrGrC mU*mU*mU* rU – 3'	IDT DNA	
mNG::CHIN-1 left homology primer (for Dickinson Lab mNG plasmid):<br>5' – GATTTTCTTC AGAATTTTAT TTATTTTCTA AGAAATTAGC TCAACTTTGC TCATTTTTCT CCAAATTTCT TCGATTTTTT TGCATTTTCA GTTAAAAAAT CAATAAAAAT CGAATTTTTG CAGGTATGGT CAGCAAAGGC GAGGAAGACA – 3'	IDT DNA	
mNG::CHIN-1 right homology primer (for Dickinson Lab mNG plasmid):<br>5' – GGAAATTGGA AAATTTGAGA TTTTAGCTTT TCGGATATTT TTAAAGCTTC CAAAACTTGT TGAGCTTGAA AAAAATGACT	IDT DNA	

TACCCGAGCT CCCAGGAGGT CCGTCGTCTT CCATCTTGTA CAGCTCGTCC ATTCCCATAA – 3’		
mNG::CHIN-1 internal left primer (for Dickinson Lab mNG plasmid): 5’ – ATGGTCAGCAAAGGCGAGGAAGACA – 3’	IDT DNA	
mNG::CHIN-1 internal right primer (for Dickinson Lab mNG plasmid): 5’ – CTTGTACAGCTCGTCCATTCCCAT – 3’	IDT DNA	
mNG::CHIN-1 FWD ODN genotyping primer: 5’ – TCTCGATCGCTGGCACTTTT – 3’	IDT DNA	
mNG::CHIN-1 REV ODN genotyping primer: 5’ – ATTCGACTCCCGCACCAAAT – 3’	IDT DNA	
Recombinant DNA		
Ahringer Feeding RNAi: *perm-1*	Source BioScience	WB Clone: sjj_T01H3.4
Ahringer Feeding RNAi: *ptr-2*	Source BioScience	WB Clone: sjj_C32E8.8
Feeding RNAi: *ctrl*	Rodriguez et al. 2017 (*21*)	N/A
Ahringer Feeding RNAi: *par-2*	Source BioScience	WB Clone: sjj_F58B6.3
Ahringer Feeding RNAi: *par-6*	Source BioScience	WB Clone: sjj_T26E3.3
Ahringer Feeding RNAi: *cyk-1*	Source BioScience	WB Clone: sjj_F11H8.4
Ahringer Feeding RNAi: *par-4*	Source BioScience	WB Clone: sjj2_Y59A8B.14
Ahringer Feeding RNAi: *par-5*	Source BioScience	WB Clone: sjj_M117.2
Ahringer Feeding RNAi: *spd-5*	Source BioScience	WB Clone: sjj_F56A3.4
Software and Algorithms		
Fiji	https://imagej.net/software/fiji/	RRID:SCR_002285
Metamorph	Molecular Devices	RRID:SCR_002368
Spectral Autofluorescence Image Correction By Regression (SAIBR)	https://github.com/goehringlab/saibr_fiji_plugin	N/A
Python	https://www.python.org/	3.8.8; RRID:SCR_008394
TensorFlow	https://www.tensorflow.org/	
Others		
Polybead Microspheres 20.00µm	Polysciences	18329-5
Polybead Microspheres 18.8µm	Polysciences	18329

Table S2. Experimental Conditions

Figure	Experiment	Drug details	Wash in timings	Wash out timings
**1C**, S2	Block cell division	Latrunculin A (0.5 µM)	Dissected embryos in buffer containing drug	N/A
**1E,F**, S4	Reversibly inhibiting CDK-1	1NA-PP1 (20 µM); 20 µM was used instead of 50 µM due to the shorter timescales required for the drug to wash out. We observed no difference in the reliability of cell cycle arrest.	Washed in drug after pronuclear meeting	Washed out drug 10-20 minutes after cells arrest
**2A**, S4	Inhibit CDK-1 activity	1NA-PP1 (50 µM)	Dissected embryos in buffer containing drug	N/A
**2C**	Inhibit CDK-1 activity and perturb cytoskeleton	1NA-PP1 (50 µM); Latrunculin A (0.5 µM); Nocodazole (1 µg/ml)	Washed in drug after pronuclear meeting	N/A
**4D**, S12	Inhibiting WEE-1	PD0166285 (20 µM)	Washed in drug during zygotic cytokinesis	N/A
**5**, S14	Inhibiting WEE-1	PD0166285 (20 µM)	Washed in drug late during P1 cytokinesis (~2 mins before completion of cell division)	N/A
S5B	Inhibit PLK-1 activity and perturb cytoskeleton	1NM-PP1 (20 µM); Latrunculin A (0.5 µM); Nocodazole (1 µg/ml)	Washed in drug after pronuclear meeting	N/A
S5C	Inhibit AIR-1 activity and perturb cytoskeleton	Alisertib (20 µM); Latrunculin A (0.5 µM); Nocodazole (1 µg/ml)	Washed in drug after pronuclear meeting	N/A
S12	Inhibiting WEE-1 and CDK-1 simultaneously	PD0166285 (20 µM); 1NA-PP1 (20 µM)	Washed in drug during zygotic cytokinesis	N/A
S12	Inhibiting WEE-1 and actomyosin cortex	PD0166285 (20 µM); Latrunculin A (0.5 µM)	Washed in PD0166285 during zygotic cytokinesis, followed by Latrunculin A 3 minutes after birth of P1.	N/A

Table S3. Parameters used for simplified PAR model

Parameter	Species	
	A (aPAR)	P (pPAR)
L	50 µm	
ψ	0.5 μm^{-1}	
$\rho_A = \rho_P$	1 μm^{-3}	
D	0.05 $\mu m^2\ s^{-1}$	
k_{off}	0.005 s^{-1}	
k_{on}	0.006 $\mu m\ s^{-1}$	
$\alpha = \beta$	2	
$k_{PA} = k_{AP}$	0.1 $\mu m^4\ s^{-1}$	
k_{cue}	0.002	

Table S4. Parameters for one-species polarity model based on the wave-pinning model

Parameter	Species (X only)
L	10 μm
ψ	1 μm^{-1}
ρ_X	3.5 μm^{-3}
D	0.01 $\mu m^2\ s^{-1}$
k_{off}	1 s^{-1}
k_{on}	0.001 $\mu m\ s^{-1}$
n	4
γ	1.2 $\mu m\ s^{-1}$
K	1 μm^{-2}

Parameters are modified to capture 4 different polarity states instead of 3 in the original model by Mori et al. (*114*).

Table S5. Parameters for *C. elegans*-specific PAR model

Parameter	Species	
	A (aPAR)	P (pPAR)
L	67.3 µm	
ψ	0.174 µm^{-1}	
ρ_A / ρ_P	ρ_A = 1.56 µm^{-3}	ρ_P = 1 µm^{-3}
D	0.28 µm^2 s^{-1}	0.15 µm^2 s^{-1}
k_{off}	5.4·10^{-3} s^{-1}	7.3·10^{-3} s^{-1}
k_{on}	8.58·10^{-3} µm s^{-1}	4.74·10^{-2} µm s^{-1}
α / β	2	
k_{PA}/ k_{AP}	k_{PA} = 3.03·10^{-2} µm^4 s^{-1}	k_{AP} = 5.61·10^{-3} µm^4 s^{-1}

All parameters used here are taken from (*53*) except from k_{PA} and k_{AP}, which were fitted with data from **Fig. 2C**.

Supplementary Movie Legends

Movie S1. PAR-1 membrane dynamics respond to changing CDK-1 activity. Midsection confocal video of an embryo expressing endogenous PAR-1::GFP in a *cdk-1as* background, acutely treated with 1NA-PP1, followed by wash out of the drug.

Movie S2. CHIN-1 membrane dynamics respond to changing CDK-1 activity. HiLo video of an embryo expressing endogenous mNG::CHIN-1 in a *cdk-1as* background, acutely treated with 1NA-PP1, followed by wash out of the drug.

Movie S3. PAR-2 and PAR-6 membrane dynamics after inhibition of CDK-1 and disruption of cytoskeleton. Midsection confocal video of an embryo expressing endogenous mCherry::PAR-2 and PAR-6::mNG in a *cdk-1as* background, acutely treated with 1NA-PP1, Latrunculin A and Nocodazole.

Movie S4. Cue-induced polarization dynamics in a simplified PAR model with constant high feedback. Simulation output reflecting cue-induced polarization beginning from (i) homogenous aPAR high state (red circle), (ii) homogenous pPAR high state (blue circle), and (iii) PA-polarized state with aPARs high at posterior and pPARs high at anterior (posterior circle), towards (iv) AP-polarized state with aPARs high at anterior and pPARs high at posterior. Note that only homogenous aPAR high states can be effectively directed towards the AP-polarized state.

Movie S5. Cue-induced polarization dynamics in a simplified PAR model with oscillatory pPAR→aPAR feedback. Simulation output reflecting cue-induced polarization beginning from (i) homogenous aPAR high state (red circle), (ii) homogenous pPAR high state (blue circle), and (iii) PA-polarized state with aPARs high at posterior and pPARs high at anterior (posterior circle), towards (iv) AP-polarized state with aPARs high at anterior and pPARs high at posterior. Compared to the constant high feedback system, all states can be effectively directed towards the AP-polarized state.

Movie S6. PKC-3 dynamics during optogenetic knocksideways of PAR-6. Midsection confocal movie of an embryo expressing PAR-6::GFP::LOV, TOMM-20::ePDZ::Halo and mScarlet-I::PKC-3 subject to acute blue light illumination and removal. Note that PAR-6 and PKC-3 appear to aggregate in the cytoplasm upon illumination, suggesting sequestration to the mitochondria.

Movie S7. Spatiotemporal dynamics of PAR-2 in P2 under WEE-1 inhibition. Midsection confocal movie of an embryo expressing GFP::PAR-2(MT-; microtubule binding defective mutant) treated with either DMSO or PD0166825 (WEE-1 inhibitor; WEEi).

REFERENCES

1. H. El-Samad, Biological feedback control—Respect the loops. *Cell Syst.* **12**, 477–487 (2021).

2. M. Freeman, Feedback control of intercellular signalling in development. *Nature* **408**, 313–319 (2000).

3. C. F. Lang, E. Munro, The PAR proteins: From molecular circuits to dynamic self-stabilizing cell polarity. *Development* **144**, 3405–3416 (2017).

4. C. E. Buckley, D. St Johnston, Apical–basal polarity and the control of epithelial form and function. *Nat. Rev. Mol. Cell Biol.* **23**, 559–577 (2022).

5. B. Goldstein, I. G. Macara, The PAR proteins: Fundamental players in animal cell polarization. *Dev. Cell* **13**, 609–622 (2007).

6. L. Rose, P. Gönczy, Polarity establishment, asymmetric division and segregation of fate determinants in early *C. elegans* embryos. *WormBook* **2014**, 1–43 (2014).

7. L. A. Koch, L. S. Rose, Multiple pathways for reestablishing PAR polarity in *C. elegans* embryo. *Dev. Biol.* **500**, 40–54 (2023).

8. K. Ng, N. Hirani, T. Bland, J. Borrego-Pinto, S. Wagner, M. Kreysing, N. W. Goehring, Cleavage furrow-directed cortical flows bias PAR polarization pathways to link cell polarity to cell division. *Curr. Biol.* **33**, 4298–4311.e6 (2023).

9. Y. Arata, J.-Y. Lee, B. Goldstein, H. Sawa, Extracellular control of PAR protein localization during asymmetric cell division in the *C. elegans* embryo. *Development* **137**, 3337–3345 (2010).

10. E. Schierenberg, Reversal of cellular polarity and early cell-cell interaction in the embryo of *Caenorhabditis elegans*. *Dev. Biol.* **122**, 452–463 (1987).

11. N. T. Rodrigues, T. Bland, K. Ng, N. Hirani, N. W. Goehring, Quantitative perturbation-phenotype maps reveal nonlinear responses underlying robustness of PAR-dependent asymmetric cell division. *PLoS Biol.* **22**, e3002437 (2024).

12. B. Etemad-Moghadam, S. Guo, K. J. Kemphues, Asymmetrically distributed PAR-3 protein contributes to cell polarity and spindle alignment in early *C. elegans* embryos. *Cell* **83**, 743–752 (1995).

13. M. Gotta, M. C. Abraham, J. Ahringer, CDC-42 controls early cell polarity and spindle orientation in *C. elegans*. *Curr. Biol.* **11**, 482–488 (2001).

14. K. T. Kumfer, S. J. Cook, J. M. Squirrell, K. W. Eliceiri, N. Peel, K. F. O'Connell, J. G. White, CGEF-1 and CHIN-1 regulate CDC-42 activity during asymmetric division in the *Caenorhabditis elegans* embryo. *Mol. Biol. Cell* **21**, 266–277 (2010).

15. Y. Tabuse, Y. Izumi, F. Piano, K. J. Kemphues, J. Miwa, S. Ohno, Atypical protein kinase C cooperates with PAR-3 to establish embryonic polarity in *Caenorhabditis elegans*. *Development* **125**, 3607–3614 (1998).

16. J. L. Watts, D. G. Morton, J. Bestman, K. J. Kemphues, The *C. elegans* par-4 gene encodes a putative serine-threonine kinase required for establishing embryonic asymmetry. *Development* **127**, 1467–1475 (2000).

17. A. Beatty, D. Morton, K. Kemphues, The *C. elegans* homolog of *Drosophila* Lethal giant larvae functions redundantly with PAR-2 to maintain polarity in the early embryo. *Development* **137**, 3995–4004 (2010).

18. L. Boyd, S. Guo, D. Levitan, D. T. Stinchcomb, K. J. Kemphues, PAR-2 is asymmetrically distributed and promotes association of P granules and PAR-1 with the cortex in *C. elegans* embryos. *Development* **122**, 3075–3084 (1996).

19. S. Guo, K. J. Kemphues, par-1, A gene required for establishing polarity in *C. elegans* embryos, encodes a putative Ser/Thr kinase that is asymmetrically distributed. *Cell* **81**, 611–620 (1995).

20. C. Hoege, A.-T. Constantinescu, A. Schwager, N. W. Goehring, P. Kumar, A. A. Hyman, LGL can partition the cortex of one-cell *Caenorhabditis elegans* embryos into two domains. *Curr. Biol.* **20**, 1296–1303 (2010).

21. J. Rodriguez, F. Peglion, J. Martin, L. Hubatsch, J. Reich, N. Hirani, A. G. Gubieda, J. Roffey, A. R. Fernandes, D. S. Johnston, aPKC cycles between functionally distinct PAR protein assemblies to drive cell polarity. *Dev. Cell* **42**, 400–415.e9 (2017).

22. A. Sailer, A. Anneken, Y. Li, S. Lee, E. Munro, Dynamic opposition of clustered proteins stabilizes cortical polarity in the *C. elegans* zygote. *Dev. Cell* **35**, 131–142 (2015).

23. R. Benton, D. St Johnston, Drosophila PAR-1 and 14-3-3 inhibit Bazooka/PAR-3 to establish complementary cortical domains in polarized cells. *Cell* **115**, 691–704 (2003).

24. F. Motegi, S. Zonies, Y. Hao, A. A. Cuenca, E. Griffin, G. Seydoux, Microtubules induce self-organization of polarized PAR domains in *Caenorhabditis elegans* zygotes. *Nat. Cell Biol.* **13**, 1361–1367 (2011).

25. A. Noatynska, N. Tavernier, M. Gotta, L. Pintard, Coordinating cell polarity and cell cycle progression: What can we learn from flies and worms? *Open Biol.* **3**, 130083 (2013).

26. S. Doerr, K. Ragkousi, Cell polarity oscillations in mitotic epithelia. *Curr. Opin. Genet. Dev.* **57**, 47–53 (2019).

27. J. D. Reich, L. Hubatsch, R. Illukkumbura, F. Peglion, T. Bland, N. Hirani, N. W. Goehring, Regulated activation of the PAR polarity network ensures a timely and specific response to spatial cues. *Curr. Biol.* **29**, 1911–1923.e5 (2019).

28. D. J. Dickinson, F. Schwager, L. Pintard, M. Gotta, B. Goldstein, A single-cell biochemistry approach reveals PAR complex dynamics during cell polarization. *Dev. Cell* **42**, 416–434.e11 (2017).

29. E. Munro, J. Nance, J. R. Priess, Cortical flows powered by asymmetrical contraction transport PAR proteins to establish and maintain anterior-posterior polarity in the early *C. elegans* embryo. *Dev. Cell* **7**, 413–424 (2004).

30. A. W. Folkmann, G. Seydoux, Spatial regulation of the polarity kinase PAR-1 by parallel inhibitory mechanisms. *Development* **146**, dev171116 (2019).

31. D. G. Morton, D. C. Shakes, S. Nugent, D. Dichoso, W. Wang, A. Golden, K. J. Kemphues, The *Caenorhabditis elegans* par-5 gene encodes a 14-3-3 protein required for cellular asymmetry in the early embryo. *Dev. Biol.* **241**, 47–58 (2002).

32. R. Ramanujam, Z. Han, Z. Zhang, P. Kanchanawong, F. Motegi, Establishment of the PAR-1 cortical gradient by the aPKC-PRBH circuit. *Nat. Chem. Biol.* **14**, 917–927 (2018).

33. Y. Wu, E. E. Griffin, Regulation of cell polarity by PAR-1/MARK kinase. *Curr. Top. Dev. Biol.* **123**, 365–397 (2017).

34. W. Bruinsma, M. Aprelia, I. García-Santisteban, J. Kool, Y. J. Xu, R. H. Medema, Inhibition of Polo-like kinase 1 during the DNA damage response is mediated through loss of Aurora A recruitment by Bora. *Oncogene* **36**, 1840–1848 (2017).

35. O. Gavet, J. Pines, Progressive activation of CyclinB1-Cdk1 coordinates entry to mitosis. *Dev. Cell* **18**, 533–543 (2010).

36. L. Macůrek, A. Lindqvist, D. Lim, M. A. Lampson, R. Klompmaker, R. Freire, C. Clouin, S. S. Taylor, M. B. Yaffe, R. H. Medema, Polo-like kinase-1 is activated by aurora A to promote checkpoint recovery. *Nature* **455**, 119–123 (2008).

37. A. C. Bishop, J. A. Ubersax, D. T. Petsch, D. P. Matheos, N. S. Gray, J. Blethrow, E. Shimizu, J. Z. Tsien, P. G. Schultz, M. D. Rose, A chemical switch for inhibitor-sensitive alleles of any protein kinase. *Nature* **407**, 395–401 (2000).

38. M. S. Lopez, J. I. Kliegman, K. M. Shokat, The logic and design of analog-sensitive kinases and their small molecule inhibitors. *Methods Enzymol.* **548**, 189–213 (2014).

39. N. Loyer, E. K. Hogg, H. G. Shaw, A. Pasztor, D. H. Murray, G. M. Findlay, J. Januschke, A CDK1 phosphorylation site on *Drosophila* PAR-3 regulates neuroblast polarisation and sensory organ formation. *Elife* **13**, e97902 (2024).

40. W. Michowski, J. M. Chick, C. Chu, A. Kolodziejczyk, Y. Wang, J. M. Suski, B. Abraham, L. Anders, D. Day, L. M. Dunkl, Cdk1 controls global epigenetic landscape in embryonic stem cells. *Mol. Cell* **78**, 459–476.e13 (2020).

41. R. P. Emptage, M. A. Lemmon, K. M. Ferguson, Molecular determinants of KA1 domain-mediated autoinhibition and phospholipid activation of MARK1 kinase. *Biochem. J.* **474**, 385–398 (2017).

42. R. P. Emptage, M. A. Lemmon, K. M. Ferguson, R. Marmorstein, Structural basis for MARK1 kinase autoinhibition by its KA1 domain. *Structure* **26**, 1137–1143.e3 (2018).

43. B. Canagarajah, F. C. Leskow, J. Y. S. Ho, H. Mischak, L. F. Saidi, M. G. Kazanietz, J. H. Hurley, Structural mechanism for lipid activation of the Rac-specific GAP, β2-chimaerin. *Cell* **119**, 407–418 (2004).

44. K. Klinkert, N. Levernier, P. Gross, C. Gentili, L. von Tobel, M. Pierron, C. Busso, S. Herrman, S. W. Grill, K. Kruse, Aurora A depletion reveals centrosome-independent polarization mechanism in *Caenorhabditis elegans*. *Elife* **8**, e44552 (2019).

45. N. I. Manzi, B. N. de Jesus, Y. Shi, D. J. Dickinson, Temporally distinct roles of Aurora A in polarization of the *C. elegans* zygote. *Development* **151**, dev202479 (2024).

46. E. M. Munro, PAR proteins and the cytoskeleton: A marriage of equals. *Curr. Opin. Cell Biol.* **18**, 86–94 (2006).

47. J. Nance, J. A. Zallen, Elaborating polarity: PAR proteins and the cytoskeleton. *Development* **138**, 799–809 (2011).

48. P. Zhao, X. Teng, S. N. Tantirimudalige, M. Nishikawa, T. Wohland, Y. Toyama, F. Motegi, Aurora-A breaks symmetry in contractile actomyosin networks independently of its role in centrosome maturation. *Dev. Cell* **48**, 631–645.e6 (2019).

49. C. Schenk, H. Bringmann, A. A. Hyman, C. R. Cowan, Cortical domain correction repositions the polarity boundary to match the cytokinesis furrow in *C. elegans* embryos. *Development* **137**, 1743–1753 (2010).

50. J. Liu, L. L. Maduzia, M. Shirayama, C. C. Mello, NMY-2 maintains cellular asymmetry and cell boundaries, and promotes a SRC-dependent asymmetric cell division. *Dev. Biol.* **339**, 366–373 (2010).

51. S. R. Naganathan, S. Fürthauer, J. Rodriguez, B. T. Fievet, F. Jülicher, J. Ahringer, C. V. Cannistraci, S. W. Grill, Morphogenetic degeneracies in the actomyosin cortex. *Elife* **7**, e37677 (2018).

52. K. A. Swan, A. F. Severson, J. C. Carter, P. R. Martin, H. Schnabel, R. Schnabel, B. Bowerman, cyk-1: A *C. elegans* FH gene required for a late step in embryonic cytokinesis. *J. Cell Sci.* **111**, 2017–2027 (1998).

53. N. W. Goehring, P. K. Trong, J. S. Bois, D. Chowdhury, E. M. Nicola, A. A. Hyman, S. W. Grill, Polarization of PAR proteins by advective triggering of a pattern-forming system. *Science* **334**, 1137–1141 (2011).

54. P. K. Trong, E. M. Nicola, N. W. Goehring, K. V. Kumar, S. W. Grill, Parameter-space topology of models for cell polarity. *New J. Phys.* **16**, 065009 (2014).

55. P. Gross, K. V. Kumar, N. W. Goehring, J. S. Bois, C. Hoege, F. Jülicher, S. W. Grill, Guiding self-organized pattern formation in cell polarity establishment. *Nat. Phys.* **15**, 293–300 (2019).

56. L. Hubatsch, F. Peglion, J. D. Reich, N. T. Rodrigues, N. Hirani, R. Illukkumbura, N. W. Goehring, A cell-size threshold limits cell polarity and asymmetric division potential. *Nat. Phys.* **15**, 1078–1085 (2019).

57. A. Nandan, A. Koseska, Non-asymptotic transients away from steady states determine cellular responsiveness to dynamic spatial-temporal signals. *PLoS Comput. Biol.* **19**, e1011388 (2023).

58. Y. Chang, D. J. Dickinson, A particle size threshold governs diffusion and segregation of PAR-3 during cell polarization. *Cell Rep.* **39**, 110652 (2022).

59. R. Illukkumbura, N. Hirani, J. Borrego-Pinto, T. Bland, K. Ng, L. Hubatsch, J. McQuade, R. G. Endres, N. W. Goehring, Design principles for selective polarization of PAR proteins by cortical flows. *J. Cell Biol.* **222**, e202209111 (2023).

60. Y. Arata, M. Hiroshima, C.-G. Pack, R. Ramanujam, F. Motegi, K. Nakazato, Y. Shindo, P. W. Wiseman, H. Sawa, T. J. Kobayashi, Cortical polarity of the RING protein PAR-2 is maintained by exchange rate kinetics at the cortical-cytoplasmic boundary. *Cell Rep.* **16**, 2156–2168 (2016).

61. T. Bland, N. Hirani, D. C. Briggs, R. Rossetto, K. Ng, I. A. Taylor, N. Q. McDonald, D. Zwicker, N. W. Goehring, Optimized PAR-2 RING dimerization mediates cooperative and selective membrane binding for robust cell polarity. *EMBO J.* **43**, 3214–3239 (2024).

62. C. Featherstone, P. Russell, Fission yeast $_{p}107^{wee1}$ mitotic inhibitor is a tyrosine/serine kinase. *Nature* **349**, 808–811 (1991).

63. K. Lundgren, N. Walworth, R. Booher, M. Dembski, M. Kirschner, D. Beach, mik1 and wee1 cooperate in the inhibitory tyrosine phosphorylation of cdc2. *Cell* **64**, 1111–1122 (1991).

64. W. M. Michael, Cyclin CYB-3 controls both S-phase and mitosis and is asymmetrically distributed in the early *C. elegans* embryo. *Development* **143**, 3119–3127 (2016).

65. P. Russell, P. Nurse, Negative regulation of mitosis by wee1+, a gene encoding a protein kinase homolog. *Cell* **49**, 559–567 (1987).

66. A. Milas, M. Jagrić, J. Martinčić, I. M. Tolić, "Optogenetic reversible knocksideways, laser ablation, and photoactivation on the mitotic spindle in human cells" in *Methods in Cell Biology* (Elsevier, 2018), vol. 145, pp. 191–215.

67. M. S. Robinson, D. A. Sahlender, S. D. Foster, Rapid inactivation of proteins by rapamycin-induced rerouting to mitochondria. *Dev. Cell* **18**, 324–331 (2010).

68. A. S. Howell, D. J. Lew, Morphogenesis and the cell cycle. *Genetics* **190**, 51–77 (2012).

69. A. Treuner-Lange, L. Søgaard-Andersen, Regulation of cell polarity in bacteria. *J. Cell Biol.* **206**, 7–17 (2014).

70. D. Devenport, D. Oristian, E. Heller, E. Fuchs, Mitotic internalization of planar cell polarity proteins preserves tissue polarity. *Nat. Cell Biol.* **13**, 893–902 (2011).

71. R. Shrestha, K. A. Little, J. V. Tamayo, W. Li, D. H. Perlman, D. Devenport, Mitotic control of planar cell polarity by polo-like kinase 1. *Dev. Cell* **33**, 522–534 (2015).

72. M. Tio, G. Udolph, X. Yang, W. Chia, cdc2 links the *Drosophila* cell cycle and asymmetric division machineries. *Nature* **409**, 1063–1067 (2001).

73. M. M. McLellan, B. L. Aerne, J. J. Banerjee Dhoul, M. V. Holder, T. Auchynnikava, N. Tapon, Meru co-ordinates spindle orientation with cell polarity and cell cycle progression. *EMBO J.* **44**, 2949–2975 (2025).

74. S. Z. Swartz, T. H. Tan, M. Perillo, N. Fakhri, G. M. Wessel, A. H. Wikramanayake, I. M. Cheeseman, Polarized Dishevelled dissolution and reassembly drives embryonic axis specification in sea star oocytes. *Curr. Biol.* **31**, 5633–5641.e4 (2021).

75. K. Ragkousi, K. Marr, S. McKinney, L. Ellington, M. C. Gibson, Cell-cycle-coupled oscillations in apical polarity and intercellular contact maintain order in embryonic epithelia. *Curr. Biol.* **27**, 1381–1386 (2017).

76. G. Jeyanathan, M. M. Cao, M. Pellikka, S. Robinson, V. Ghorayeb, P. Talukder, U. Tepass, Mitotic polarity oscillation promotes epithelial tumor progression. bioRxiv 2025.02.06.636979 [Preprint] (2025). https://doi.org/10.1101/2025.02.06.636979.

77. L. N. Deutz, S. Sarıkaya, D. J. Dickinson, Membrane extraction in native lipid nanodiscs reveals dynamic regulation of Cdc42 complexes during cell polarization. *Biophys. J.* **124**, 876–890 (2023).

78. J. Packer, A. G. Gubieda, A. Brooks, L. N. Deutz, I. Squires, S. Ellison, C. Schneider, S. R. Naganathan, A. J. Wollman, D. J. Dickinson, Atypical protein kinase C promotes its own asymmetric localisation by phosphorylating Cdc42 in the *C. elegans* zygote. bioRxiv 2023.10.27.563985 [Preprint] (2024). https://doi.org/10.1101/2023.10.27.563985.

79. F. Wirtz-Peitz, T. Nishimura, J. A. Knoblich, Linking cell cycle to asymmetric division: Aurora-A phosphorylates the Par complex to regulate Numb localization. *Cell* **135**, 161–173 (2008).

80. M. Das, T. Drake, D. J. Wiley, P. Buchwald, D. Vavylonis, F. Verde, Oscillatory dynamics of Cdc42 GTPase in the control of polarized growth. *Science* **337**, 239–243 (2012).

81. W. R. Holmes, J. Park, A. Levchenko, L. Edelstein-Keshet, A mathematical model coupling polarity signaling to cell adhesion explains diverse cell migration patterns. *PLoS Comput. Biol.* **13**, e1005524 (2017).

82. H. Meinhardt, Orientation of chemotactic cells and growth cones: Models and mechanisms. *J. Cell Sci.* **112**, 2867–2874 (1999).

83. H. Meinhardt, A. Gierer, Applications of a theory of biological pattern formation based on lateral inhibition. *J. Cell Sci.* **15**, 321–346 (1974).

84. L. Plazen, J. A. Rahbani, C. M. Brown, A. Khadra, Polarity and mixed-mode oscillations may underlie different patterns of cellular migration. *Sci. Rep.* **13**, 4223 (2023).

85. A. Jilkine, L. Edelstein-Keshet, A comparison of mathematical models for polarization of single eukaryotic cells in response to guided cues. *PLoS Comput. Biol.* **7**, e1001121 (2011).

86. J. P. Town, O. D. Weiner, Local negative feedback of Rac activity at the leading edge underlies a pilot pseudopod-like program for amoeboid cell guidance. *PLoS Biol.* **21**, e3002307 (2023).

87. N. Loyer, J. Januschke, Where does asymmetry come from? Illustrating principles of polarity and asymmetry establishment in Drosophila neuroblasts. *Curr. Opin. Cell Biol.* **62**, 70–77 (2020).

88. C. H. Oon, K. E. Prehoda, Asymmetric recruitment and actin-dependent cortical flows drive the neuroblast polarity cycle. *Elife* **8**, e45815 (2019).

89. C. H. Oon, K. E. Prehoda, Phases of cortical actomyosin dynamics coupled to the neuroblast polarity cycle. *Elife* **10**, e66574 (2021).

90. G. P. Bell, G. C. Fletcher, R. Brain, B. J. Thompson, Aurora kinases phosphorylate Lgl to induce mitotic spindle orientation in *Drosophila epithelia*. *Curr. Biol.* **25**, 61–68 (2015).

91. C. A. Carvalho, S. Moreira, G. Ventura, C. E. Sunkel, E. Morais-de-Sá, Aurora A triggers Lgl cortical release during symmetric division to control planar spindle orientation. *Curr. Biol.* **25**, 53–60 (2015).

92. S. Le Bras, R. Le Borgne, Epithelial cell division—Multiplying without losing touch. *J. Cell Sci.* **127**, 5127–5137 (2014).

93. S. Moreira, M. Osswald, G. Ventura, M. Gonçalves, C. E. Sunkel, E. Morais-de-Sá, PP1-mediated dephosphorylation of Lgl controls apical-basal polarity. *Cell Rep.* **26**, 293–301.e7 (2019).

94. M. Osswald, E. Morais-de-Sa, Dealing with apical–basal polarity and intercellular junctions: A multidimensional challenge for epithelial cell division. *Curr. Opin. Cell Biol.* **60**, 75–83 (2019).

95. K. E. Miller, P. J. Kang, H.-O. Park, Regulation of Cdc42 for polarized growth in budding yeast. *Microb. Cell* **7**, 175–189 (2020).

96. K. D. Moran, H. Kang, A. V. Araujo, T. R. Zyla, K. Saito, D. Tsygankov, D. J. Lew, Cell-cycle control of cell polarity in yeast. *J. Cell Biol.* **218**, 171–189 (2019).

97. K. Witte, D. Strickland, M. Glotzer, Cell cycle entry triggers a switch between two modes of Cdc42 activation during yeast polarization. *Elife* **6**, e26722 (2017).

98. U. Kadiyala, D. Sprinzak, N. A. Monk, S. E. Taylor, B. Verd, K. F. Sonnen, L. Moon, A. H. Roeder, R. Perez-Carrasco, P. Formosa-Jordan, From genes to patterns: Five key dynamical systems concepts to decode developmental regulatory mechanisms. *Development* **152**, dev204617 (2025).

99. D. J. Cislo, M. J. Delás, J. Briscoe, E. D. Siggia, Reconstructing Waddington's landscape from data. bioRxiv 2025.08.11.669575 [Preprint] (2025). https://doi.org/10.1101/2025.08.11.669575.

100. M. Sáez, J. Briscoe, D. A. Rand, Dynamical landscapes of cell fate decisions. *Interface Focus* **12**, 20220002 (2022).

101. J. Rombouts, M. L. Zhao, A. Aulehla, A. Erzberger, System size and boundaries determine the patterning dynamics of attracting active particles. arXiv:2509.08533 [nlin.PS] (2025).

102. F. Corson, E. D. Siggia, Gene-free methodology for cell fate dynamics during development. *Elife* **6**, e30743 (2017).

103. T. Stiernagle, Maintenance of *C. elegans*. *WormBook* **2006**, 1–11 (2006).

104. J. A. Arribere, R. T. Bell, B. X. Fu, K. L. Artiles, P. S. Hartman, A. Z. Fire, Efficient marker-free recovery of custom genetic modifications with CRISPR/Cas9 in *Caenorhabditis elegans*. *Genetics* **198**, 837–846 (2014).

105. G. A. Dokshin, K. S. Ghanta, K. M. Piscopo, C. C. Mello, Robust genome editing with short single-stranded and long, partially single-stranded DNA donors in *Caenorhabditis elegans*. *Genetics* **210**, 781–787 (2018).

106. R. S. Kamath, J. Ahringer, Genome-wide RNAi screening in *Caenorhabditis elegans*. *Methods* **30**, 313–321 (2003).

107. N. W. Goehring, C. Hoege, S. W. Grill, A. A. Hyman, PAR proteins diffuse freely across the anterior–posterior boundary in polarized *C. elegans* embryos. *J. Cell Biol.* **193**, 583–594 (2011).

108. L. G. Edgar, B. Goldstein, "Culture and manipulation of embryonic cells" in *Methods in Cell Biology* (Elsevier, 2012), vol. 107, pp. 151–175.

109. C. R. Hsu, R. Xiong, K. Sugioka, In vitro reconstitution of spatial cell contact patterns with isolated *Caenorhabditis elegans* embryo blastomeres and adhesive polystyrene beads. *J. Vis. Exp.* **153**, e60422 (2019).

110. C. A. Shelton, B. Bowerman, Time-dependent responses to glp-1-mediated inductions in early *C. elegans* embryos. *Development* **122**, 2043–2050 (1996).

111. M. Sandler, A. Howard, M. Zhu, A. Zhmoginov, L.-C. Chen, "Mobilenetv2: Inverted residuals and linear bottlenecks," in *Proceedings of the IEEE Conference on Computer Vision and Pattern Recognition* (IEEE, 2018), pp. 4510–4520.

112. N. T. L. Rodrigues, T. Bland, J. Borrego-Pinto, K. Ng, N. Hirani, Y. Gu, S. Foo, N. W. Goehring, SAIBR: a simple, platform-independent method for spectral autofluorescence correction. *Development* **149**, dev200545 (2022).

113. K. Ng, T. Bland, N. Hirani, N. W. Goehring, An analog sensitive allele permits rapid and reversible chemical inhibition of PKC-3 activity in *C. elegans*. *MicroPubl. Biol.* **2022**, 10.17912/micropub.biology.000610 (2022).

114. Y. Mori, A. Jilkine, L. Edelstein-Keshet, Wave-pinning and cell polarity from a bistable reaction-diffusion system. *Biophys. J.* **94**, 3684–3697 (2008).

115. M. P. Dalwadi, P. Pearce, Universal dynamics of biological pattern formation in spatio-temporal morphogen variations. *Proc. R. Soc. London Ser. A Math. Phys. Eng. Sci.* **479**, 20220829 (2023).

116. S. Blanchoud, C. Busso, F. Naef, P. Goenczy, Quantitative analysis and modeling probe polarity establishment in *C. elegans* embryos. *Biophys. J.* **108**, 799–809 (2015).

117. V. Holubec, K. Kroy, S. Steffenoni, Physically consistent numerical solver for time-dependent Fokker-Planck equations. *Phys. Rev. E* **99**, 032117 (2019).

118. J. R. Dormand, P. J. Prince, A family of embedded Runge-Kutta formulae. *J. Comput. Appl. Math.* **6**, 19–26 (1980).

119. M. Cobbaut, N. Q. McDonald, P. J. Parker, Control of atypical PKCι membrane dissociation by tyrosine phosphorylation within a PB1-C1 interdomain interface. *J. Biol. Chem.* **299**, 104847 (2023).

120. B. Han, K. R. Antkowiak, X. Fan, M. Rutigliano, S. P. Ryder, E. E. Griffin, Polo-like kinase couples cytoplasmic protein gradients in the *C. elegans* zygote. *Curr. Biol.* **28**, 60–69.e8 (2018).

121. T. B. Sells, R. Chau, J. A. Ecsedy, R. E. Gershman, K. Hoar, J. Huck, D. A. Janowick, V. J. Kadambi, P. J. LeRoy, M. Stirling, S. G. Stroud, T. J. Vos, G. S. Weatherhead, D. R. Wysong, M. Zhang, S. K. Balani, J. B. Bolen, M. G. Manfredi, C. F. Claiborne, MLN8054 and Alisertib (MLN8237): Discovery of selective oral Aurora A inhibitors. *ACS Med. Chem. Lett.* **6**, 630–634 (2015).

122. S. De Henau, M. Pagès-Gallego, W.-J. Pannekoek, T. B. Dansen, Mitochondria-derived H_2O_2 promotes symmetry breaking of the *C. elegans* zygote. *Dev. Cell* **53**, 263–271.e6 (2020).

123. L.-E. Fielmich, R. Schmidt, D. J. Dickinson, B. Goldstein, A. Akhmanova, S. Van Den Heuvel, Optogenetic dissection of mitotic spindle positioning in vivo. *Elife* **7**, e38198 (2018).

124. M. G. Manfredi, J. A. Ecsedy, A. Chakravarty, L. Silverman, M. Zhang, K. M. Hoar, S. G. Stroud, W. Chen, V. Shinde, J. J. Huck, Characterization of Alisertib (MLN8237), an investigational small-molecule inhibitor of aurora A kinase using novel in vivo pharmacodynamic assays. *Clin. Cancer Res.* **17**, 7614–7624 (2011).
